# Application of a New Dual Localization-Affinity Purification Tag Reveals Novel Aspects of Protein Kinase Biology in *Aspergillus nidulans*

**DOI:** 10.1371/journal.pone.0090911

**Published:** 2014-03-05

**Authors:** Colin P. De Souza, Shahr B. Hashmi, Aysha H. Osmani, Stephen A. Osmani

**Affiliations:** Department of Molecular Genetics, The Ohio State University, Columbus, Ohio, United States of America; University of Wisconsin - Madison, United States of America

## Abstract

Filamentous fungi occupy critical environmental niches and have numerous beneficial industrial applications but devastating effects as pathogens and agents of food spoilage. As regulators of essentially all biological processes protein kinases have been intensively studied but how they regulate the often unique biology of filamentous fungi is not completely understood. Significant understanding of filamentous fungal biology has come from the study of the model organism *Aspergillus nidulans* using a combination of molecular genetics, biochemistry, cell biology and genomic approaches. Here we describe dual localization-affinity purification (DLAP) tags enabling endogenous N or C-terminal protein tagging for localization and biochemical studies in *A. nidulans*. To establish DLAP tag utility we endogenously tagged 17 protein kinases for analysis by live cell imaging and affinity purification. Proteomic analysis of purifications by mass spectrometry confirmed association of the CotA and NimX^Cdk1^ kinases with known binding partners and verified a predicted interaction of the SldA^Bub1/R1^ spindle assembly checkpoint kinase with SldB^Bub3^. We demonstrate that the single TOR kinase of *A. nidulans* locates to vacuoles and vesicles, suggesting that the function of endomembranes as major TOR cellular hubs is conserved in filamentous fungi. Comparative analysis revealed 7 kinases with mitotic specific locations including An-Cdc7 which unexpectedly located to mitotic spindle pole bodies (SPBs), the first such localization described for this family of DNA replication kinases. We show that the SepH septation kinase locates to SPBs specifically in the basal region of apical cells in a biphasic manner during mitosis and again during septation. This results in gradients of SepH between G1 SPBs which shift along hyphae as each septum forms. We propose that SepH regulates the septation initiation network (SIN) specifically at SPBs in the basal region of G1 cells and that localized gradients of SIN activity promote asymmetric septation.

## Introduction

Filamentous fungi have enormous ecological, medical, agricultural and industrial impact and understanding their often unique cell biology is of great importance [1]. Some members of genus Aspergillus have major economical benefits in the production of citric acid, sake and soy sauce while other species, particularly *Aspergillus fumigatus*, are opportunistic pathogens having the ability to cause systemic Aspergillosis in immuno-compromised patients [2]. *Aspergillus nidulans* is a powerful model genetic system and one of an impressive and growing number of sequenced filamentous fungal genomes including 19 species of Aspergilli (http://www.aspgd.org/) [3], [4]. Improved annotation of the *A. nidulans* genome based on RNA-seq data together with advances in transcriptome analysis, endogenous gene targeting and the availability of gene deletion constructs for over 93% of genes have set the stage for further functional genomics [5]–[9]. This enhanced gene targeting has also facilitated rapid endogenous targeting of affinity purification tags or fluorescent proteins for proteomic studies allowing the mapping of protein interaction networks and defining subcellular protein localizations. Such approaches have significantly advanced the understanding of fungal biology [10]–[22].

Differential regulation of asymmetric septation in between nuclei along filamentous fungal hyphae contributes greatly to their often unique cell biology. Multinucleate *A. nidulans* hyphae arise from the growth of uninucleate conidiospores because septation is initially suppressed during the first few cell cycles [23], [24]. Septation then becomes coupled with the cell cycles of multinucleate tip cells which undergo parasynchronous mitosis but do not form septa between each nucleus. Although many genes regulating septation have been identified [5], [24]–[32] how septation is differentially regulated to occur asymmetrically along the length of hyphae is not understood.

Protein kinases are involved in the regulation of virtually all eukaryotic biological processes through the reversible phosphorylation of their substrates and have thus been the subject of intense study [5], [33]–[39]. Recently the kinome of *A. nidulans* has been analyzed using functional genomics to generate and phenotypically analyze deletion mutants of all 128 protein kinases [5]. Kinases are regulated at the level of cellular protein levels, association with regulatory proteins, post-translational modifications and subcellular localization [40]–[44]. Thus proteomic approaches to define interacting proteins, posttranslational modifications and changes in the cellular levels or localization of kinases are useful to understand kinase biology. Study of kinase biology in *A. nidulans* has provided insights into both universally conserved kinase functions as well as fungal specific functions. For example the multifunctional NIMA kinase is the founding member of the NIMA related kinase (NEK) family and is essential for mitotic entry in *A. nidulans* and *Magnaporthe grisea* [21], [45]–[47]. Several of the 11 human NEKs perform similar mitotic functions to NIMA [48], [49].

Protein localization is often indicative of function and this is perhaps most clearly illustrated by the location of mitotic kinases. For example during mitotic progression mammalian Aurora B kinases sequentially locate to, and have functions at, chromosomal arms, centromeres, the spindle midzone, the equatorial cortex and the midbody [40], [50], [51]. While Aurora B does not apparently have an interphase function other mitotic kinases locate to interphase structures where they have functions distinct from their mitotic functions [42]. For example, during interphase mammalian Mps1 kinases locate to centrosomes and regulate their duplication but during mitosis they locate to kinetochores where they are required to activate the spindle assembly checkpoint (SAC) [42], [52]. The SAC helps ensure faithful mitosis by delaying anaphase onset until all kinetochores are correctly attached to the spindle and recruitment of SAC proteins to mitotic kinetochores is a hallmark of SAC activation [53], [54]. Moreover, the human Prp4 kinase was unexpectedly found to locate to mitotic kinetochores and subsequently shown to function in the SAC although it was previously thought to function only in RNA processing [55]. Thus changes in the location of kinases from interphase to mitosis are often predictive of their function.

*A. nidulans* offers many advantages for proteomic studies defining protein interactions and subcellular localizations. Aiding biochemical analysis is the ability to produce an enormous number of genetically identical conidia (asexual spores) for the rapid generation of biomass which can easily be harvested by filtration [16]. Methods are in place for single step affinity purification of proteins endogenously tagged with the 15 amino acid S-tag using S-protein beads for downstream mass spectrometry analysis to identify associated proteins, define post-translational modifications and complete biochemical assays [16], [21], [56]–[58]. *A. nidulans* is also compatible with live cell imaging of proteins endogenously tagged with fluorescent markers and attaches to glass coverslips helping facilitate imaging over long time periods of polarized growth [59]. To streamline proteomic analysis in *A. nidulans* we describe here dual localization-affinity purification (DLAP) tags comprised of GFP in tandem with the S-tag for endogenous C-terminal or N-terminal protein tagging. To establish the utility of DLAP tags and advance the understanding of fungal kinase biology, we have endogenously tagged, localized and affinity purified 17 *A. nidulans* protein kinases and completed mass spectrometry analysis for 10 affinity purified kinases. Analysis of this data demonstrated that DLAP tagged versions of the CotA, SldA^Bub1/R1^ and NimX^Cdk1^ kinases each specifically co-purify associated proteins. Comparative analysis of interphase and mitotic localizations indicated that 13 kinases changed location from interphase to mitosis and 7 kinases located to mitotic structures. Our data suggest functions for the An-Cdc7 DNA replication kinase at mitotic SPBs, the TorA kinase at endomembranes and provide novel insights into how SepH kinase location to SPBs differentially regulates septation generating asymmetric cell divisions along the length of hyphae.

## Results and Discussion

**Figure 1:**
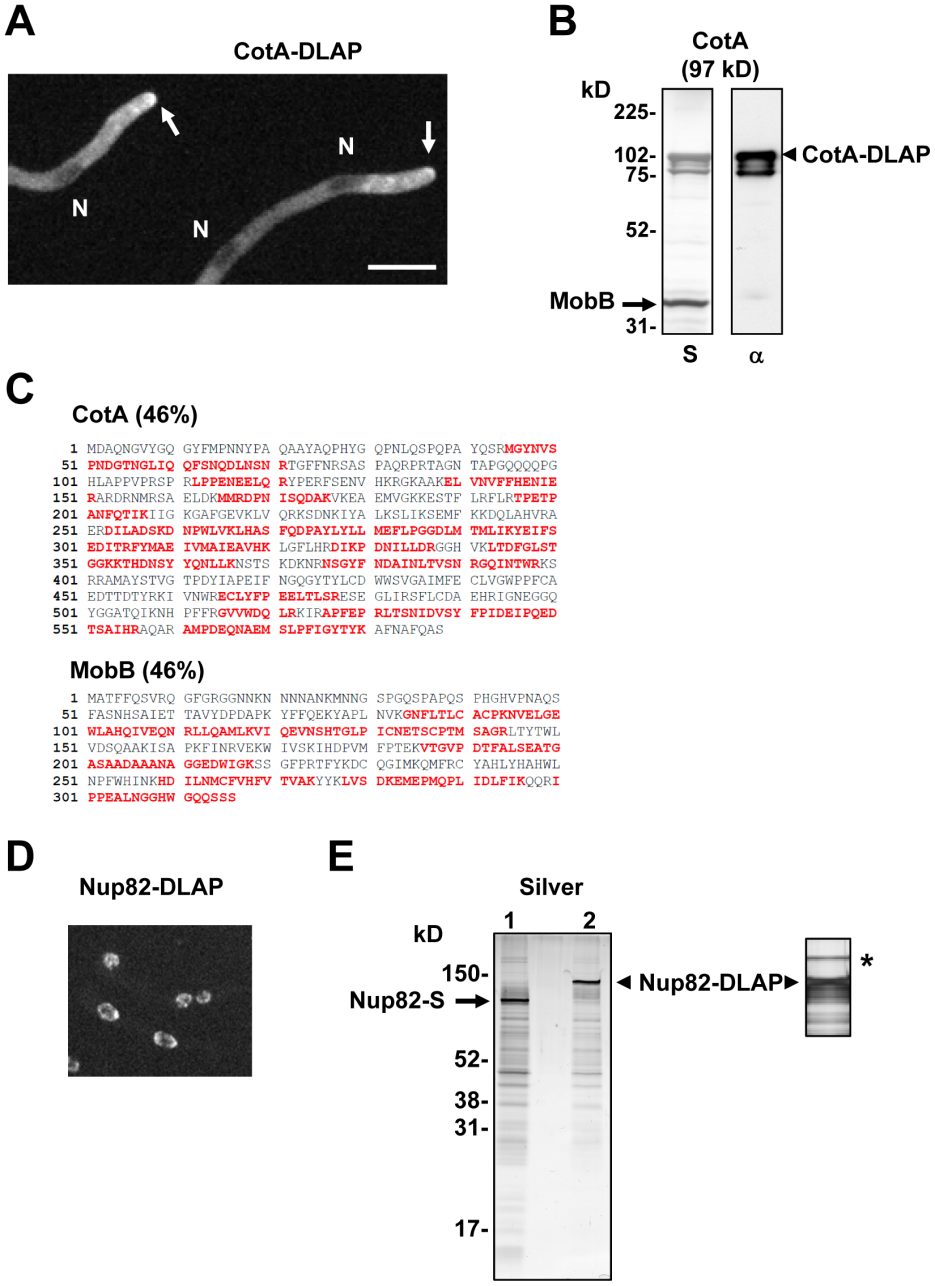
The DLAP tag (GFP-S-Tag) facilitates the localization and affinity purification of endogenously tagged proteins. **A** Micrograph showing that Cot-DLAP locates to the cytoplasm and is enriched at growing cell tips (arrows). N = nuclei, Bar ∼10 µm. **B** Silver stained SDS-PAGE gel (S) and anti-GFP western blot (α) of a single step CotA-DLAP affinity purification using S-protein beads. **C** Sequence of CotA and MobB (AN1370) with peptides identified by LC-MS/MS analysis shown in red. **D** Micrograph showing Nup82-DLAP locates to the nuclear periphery. **E** Silver stained SDS-PAGE gel of single step affinity purifications of Nup82-S-tag (lane 1) and Nup82-DLAP (lane 2). The asterisk indicates a higher molecular weight band corresponding to the electrophoretic mobility of the Nup82 binding partner Nup159 visible following a longer period of silver staining of a another Nup82-DLAP purification.

We recently developed a protocol for single step affinity purification of *A. nidulans* proteins endogenously tagged with the S-tag [16]. To additionally allow localization of proteins to be affinity purified we generated a dual localization-affinity purification (DLAP) tag (GFP-S-tag::*pyrG* ^Af^) for endogenous C-terminal tagging using standard *A. nidulans* primers [16], [22]. This tag includes 10 amino acid spacers separating the C-terminus of the tagged protein and the GFP and also in between the GFP and the S-tag to help provide functionality of the GFP, the S-tag and the tagged protein (Supplemental file S2). To test DLAP functionality we endogenously tagged the essential CotA kinase which localizes to the cytoplasm and cell tips and interacts with its binding partner MobB in a yeast 2-hybrid assay [60]. A CotA-DLAP::*pyrG^Af^* tagging construct generated by standard 3-way fusion PCR was transformed into a Δ*nkuA^Ku70^* recipient strain in order to facilitate a high frequency of homologous gene replacement (Figure S1 in Supplementary File S1) [7], [22]. Successful integration of the DLAP tag at the C-terminal was confirmed by diagnostic PCR using primers situated external to the targeting construct. Live cell confocal imaging indicated that CotA-DLAP displayed its expected cytoplasmic localization and was enriched at growing cell tips (Figure 1A). We next carried out affinity purification of CotA-DLAP using S-protein agarose beads and recently described methodologies [16]. The main feature of this purification protocol is that lysates are prepared from lyophilized cells. To ensure samples contained cells in a range of developmental stages, we prepared lysates from cells which had begun asexual reproduction in static liquid Petri dish cultures. Examination of the CotA-DLAP purification by silver staining of SDS-PAGE gels revealed two prominent bands, one corresponding to the 97 kD CotA-DLAP fusion and one with a mobility consistent with the 35 kD MobB protein (Figure 1B). To confirm that MobB co-purified with CotA, the purified sample was prepared for LC-MS/MS by running an SDS-PAGE gel until the proteins had just entered the separating gel and excising the bands as a single gel slice for analysis [16]. LC-MS/MS analysis identified CotA with 46% sequence coverage as well as 9 peptides with 46% sequence coverage for MobB (AN1370). Together this indicates that CotA-DLAP allows the correct localization of CotA and facilitates affinity purification of the CotA-MobB protein complex.

To directly compare affinity purifications using either the DLAP tag or the S-tag alone we endogenously tagged the essential Nup82 nuclear pore complex (NPC) protein which localizes to the nuclear periphery and forms a complex with Nup159 [13] (AO and SAO unpublished). Live cell confocal imaging indicated that Nup82-DLAP displayed its expected localization to the nuclear periphery (Figure 1D). Affinity purification using S-protein agarose beads purified both Nup82-DLAP and Nup82-S-tag with similar efficiency and the Nup82-DLAP affinity purification contained a band corresponding with the predicted size of Nup159 (Figure 1E). Thus proteins endogenously tagged with either the DLAP or the S-tag alone can be similarly purified using S-protein agarose beads. However the DLAP tag additionally allows localization of tagged proteins and offers the potential of an addition purification step using GFP-based affinity purification methodologies.

### Protein Kinase Localization and Proteomics using the DLAP Tag

We selected 16 additional kinases to C-terminally DLAP tag for localization and proteomic analysis. NimX^Cdk1^, UvsB^ATR^, CmkA, SepH, SldA^Bub1/R1^, and SudD^Rio1^, were tagged at experimentally verified C-terminals [25], [61]–[66]. The ChkC, Aurora, IreA, BckA, An-Prp4, TorA, An-Cdc7 and An-Cdk7 kinases were tagged at conserved predicted C-terminals which have recently been confirmed by RNA-Seq data (http://www.aspgd.org/) [3], [9] and in the case of Aurora also by 3′ RACE-PCR (CDS and SAO unpublished). As alignment of the predicted CkiA^Hrr25^ C-terminal with orthologous Aspergillus kinases was ambiguous, we performed 3′-RACE PCR which defined the C-terminus as GLGRQWYYEA*. For each of these kinases specific C-terminal DLAP constructs were generated, transformed into the same recipient strain and site specific transformants identified as above. In total 15 of the 17 C-terminally DLAP tagged kinases were functional as they complemented the phenotypes caused by deletion of the respective kinase genes (Table S1 in Supplementary File S1) [5]. However, C-terminal DLAP tagged versions of the UvsB^ATR^ and TorA PIKK kinases were non-functional as the tagged kinase alleles phenocopied the null alleles (Table S1 and Figure S2C in Supplementary File S1). We thus constructed a DLAP cassette (S-tag-GFP) (Supplemental file S3) for endogenous N-terminal protein tagging via a five way fusion PCR regime and N-terminally tagged UvsB^ATR^ and TorA (Figure S2B and C in Supplementary File S1) [11]. Verified DLAP-UvsB^ATR^ strains did not display the genotoxic stress sensitivity of *uvsB^ATR^* nulls and DLAP-TorA strains did not display the lethal phenotype of the *torA* nulls although they displayed a minor growth defect (Figure S2C in Supplementary File S1). Thus, unlike the C-terminally tagged proteins, N-terminally tagged versions of UvsB^ATR^ and TorA are functional.

**Figure 2:**
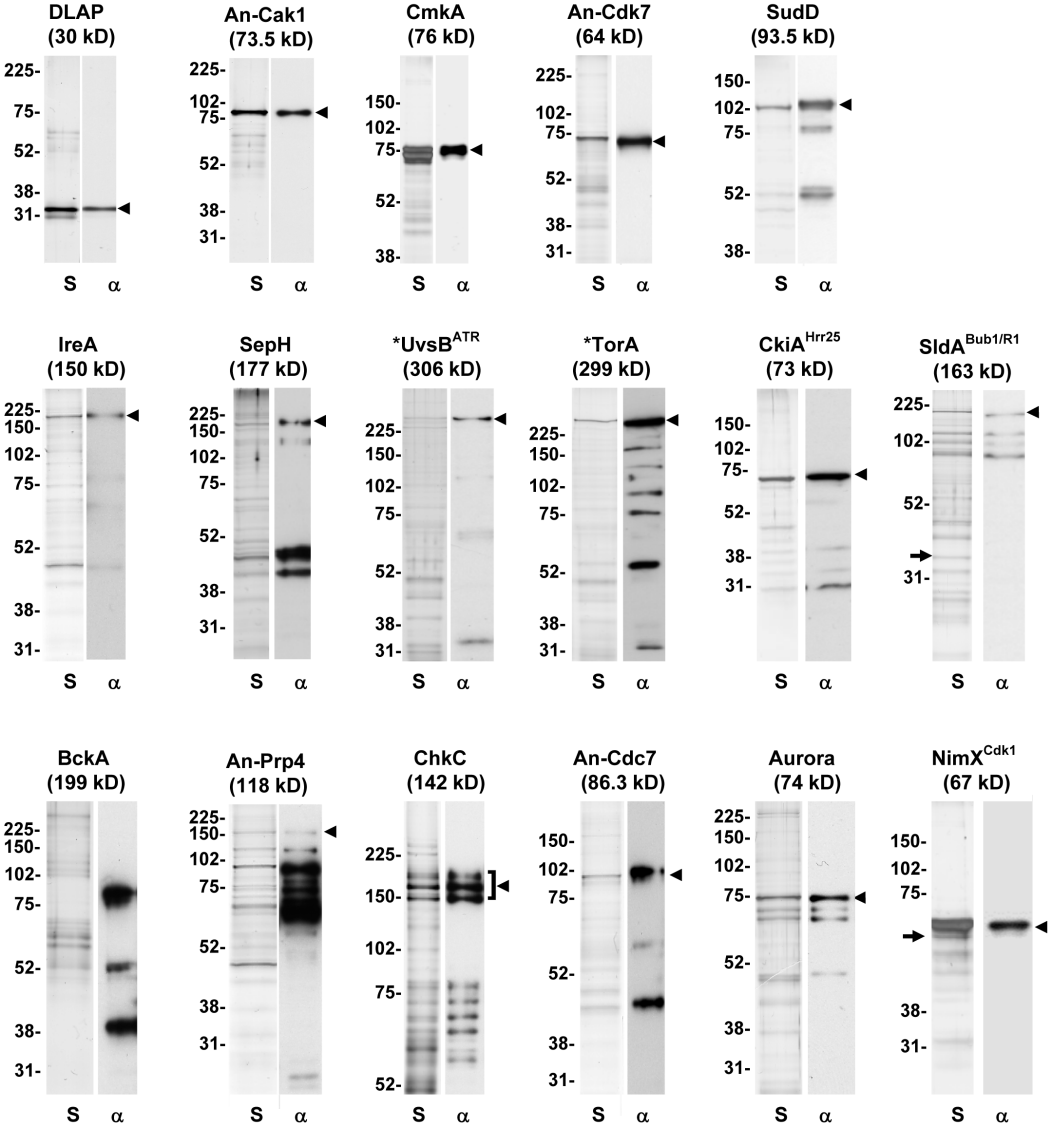
Single step affinity purifications of endogenously DLAP tagged kinases. Shown are silver stained SDS-PAGE gels (S) and anti-GFP western blots (α) of the indicated affinity purified kinases and a DLAP control. Molecular weights indicated in parentheses are for the respective DLAP tagged kinase. Arrowheads indicate the full length DLAP tagged kinase. Arrows indicate silver staining bands corresponding to the predicted molecular weight of co-purifying proteins identified by LC-MS/MS. * = N-terminally tagged.

Each of the DLAP tagged kinase strains were next analyzed by live cell confocal microscopy to define their localization and affinity purifications carried out to purify the respective kinase and associated proteins. As a control DLAP expressed on its own was similarly analyzed. Following affinity purification approximately 10% of each purified sample was run on SDS-PAGE gels for silver staining and Western blotting using an anti-GFP antibody. Analysis of the DLAP control purification revealed a strong band at the expected molecular weight with only a few contaminating proteins visible by silver staining. Comparison of the silver stained gels and anti-GFP Western blots revealed each kinase affinity purification contained the respective DLAP tagged kinase although in some cases significant degradation products were present (Figure 2). Such degradation occurred during the purification procedure despite the presence of protease inhibitors and might reflect endogenous proteolysis taking place in the older parts of the conidiating cultures employed. To further test the robustness of the DLAP tag, 10 kinase purifications were analyzed by LC-MS/MS to determine sequence coverage of the respective purified kinase and identify associated proteins. The control DLAP construct purification was similarly analyzed. Sequence coverage obtained for each of the 10 purified kinases ranged from 15% for the lowly abundant SepH to 71% for the abundant NimX^Cdk1^ kinase (Table 1). Comparative analysis of proteins identified by LC-MS/MS in each kinase purification and the DLAP control was used to determine specific co-purifying proteins. For example, MobB, which co-purified with CotA as described above, was not identified in the other 9 kinase purifications or in the DLAP control, indicating that this interaction is specific. This comparison also allowed common contaminants present in multiple purifications or in the DLAP control to be removed from further analysis. The results for the kinase localization and proteomic studies are summarized in Table 1 and described in more detail below.

**Table 1:** Summary of the kinase localization and proteomic studies.

Tagged protein	Tag location	Interphase location	Mitotic location	LC-MS/MS hits	Name	peptides	% Coverage
An-Cak1	SSSKKSTTAV*	Throughout cell	ND	**AN0699**	An-Cak1	14	51
CmkA	EREARERAHS*	Cytoplasmic	Throughout cell	**AN2412**	CmkA	17	58
NimX^Cdk1^	SGRARRNGFH*	Nuclear enriched, G2 SPBs	SPBs and nucleus (nuclear	**AN4182**	NimX^Cdk1^	19	71
			envelope associated foci	AN3648	Cyclin B	7	21
			during SAC arrest)	AN3795	PucA	1@	1
SepH	LTQFEAERGS*	Cytoplasmic foci, SPBs	Cytoplasmic foci, SPBs	**AN4385**	SepH	14	15
CkiA^hrr25^	GLGRQWYYEA* &	Nuclear enriched, septa	SPBs, septa, cytoplasmic foci	**AN4563**	CkiA^hrr25^	17	50
CotA	YKAFNAFQAS*	Cytoplasmic, tip enriched	Throughout cell, tip enriched	**AN5529**	CotA	22	46
				AN1370	MobB	9	46
An-Aurora	GSGASKDGKV*	Cytoplasmic	The mitotic apparatus	**AN5815**	An-Aurora	17	53
SudD^Rio1^	LVSSSSRKRK*	Cytoplasmic	Throughout cell	**AN6363**	SudD	26	47
ChkC	PVRRNAISKE*	Nuclear (Variable)	Nuclear	**AN7563**	ChkC	16	25
SldA^Bub1/R1^	FAEKKKRLEK*	Not detected	Kinetochores	**AN3946**	SldA^Bub1/R1^	22	32
				AN2439	SldB^Bub3^	6	22
An-IreA	RFKRYFTPLE*	ER-like	ND	ND			
BckA	YAKIRPVLEN*	Cytoplasmic, weakly enriched at septa	Throughout cell	ND			
An-Prp4	KH PFILRPKA*	Nuclear, nucleolar excluded	Dispersed	ND			
UvsB^ATR^	*MGMSDWASVE	Not detected	ND	ND			
TorA	*MAQAGPITDV	Vacuoles, cytoplasmic foci	ND	ND			
An-Cdc7	DGDDDEVDMV*	Nuclear enriched	SPBs	ND			
An-Cdk7	RQLDFGAIKG*	Nuclear enriched, nucleolar excluded	Dispersed	ND			
DLAP (Control)		Throughout cell	ND	DLAP		7	35

*The position of the in frame fusion with the DLAP tag.

AN#s in bold indicate the tagged kinase.

ND = not determined.

& for An-CkiA^Hrr25^ the C-terminus was determined by 3′ RACE PCR.

@ An-Puc1 peptides were present in an independent NimXcdk1-DLAP purification.

#### DLAP-tagged An-Cak1 and UvsB^ATR^ do not display defined cellular locations

**Figure 3:**
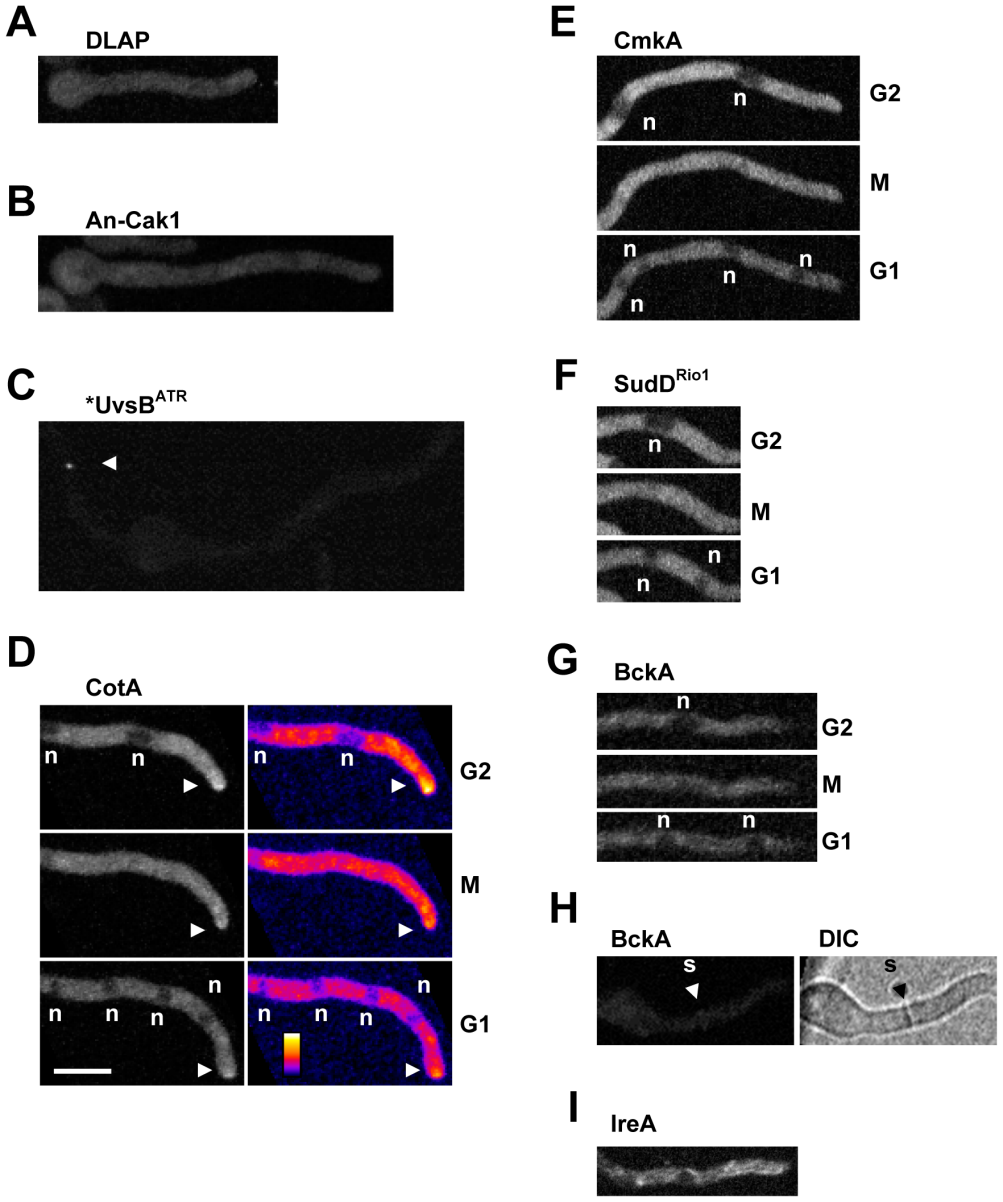
Kinases with a cytoplasmic localization. **A** A DLAP construct does not locate to any cellular compartment. **B** An-Cak1-DLAP locates throughout the cell. **C** DLAP-UvsB^ATR^ displays no obvious location apart from rare foci (arrowhead). **D** CotA-DLAP is cytoplasmic and is enriched at cell tips (arrowheads) but enters nuclei during mitosis. False coloring more clearly depicts its location to the cell tip. **E–G** CmkA-DLAP, BckA-DLAP and SudD^Rio1^-DLAP are cytoplasmic during interphase but enter nuclei during nuclear division. **H** BckA-DLAP locates weakly to septa. **I** IreA-DLAP locates to a cytoplasmic structure consistent with its predicted location to the ER. n = nuclei, Bar ∼10 µm.

Apart from An-Cak1, which is predicted to activate cyclin dependent kinases, and the UvsB^ATR^ DNA damage response kinase [5], [61], [67], all kinases displayed a distinct localization. An-Cak1-DLAP localized throughout cells in a manner similar to the DLAP control (Figure 3B) and unlike *S. cerevisiae* Cak1 was not obviously excluded from nuclei [68]. Apart from rare foci, we were unable to detect DLAP-UvsB^ATR^ above background levels (Figure 3C) even though we were able to affinity purify the full length protein (Figure 2). Given that human ATR locates to nuclear foci following genotoxic stress [69] we examined UvsB^ATR^ location following the addition of either the DNA replication inhibitor hydroxyurea, or the DNA alkylating agent DEO. However, even under these conditions of genotoxic stress, we were unable to detect a distinct UvsB^ATR^ localization in 6 independent transformants (data not shown). We note that localization studies of the UvsB^ATR^ orthologue Mec1p in *S. cerevisiae* were also unsuccessful [68], [70]. One possibility is that misfolding of GFP when fused with UvsB^ATR^ compromises its fluorescence.

### Cytoplasmic Kinases

Endogenously DLAP tagged versions of the CmkA, SudD^Rio1^ and BckA kinases each localized throughout the cytoplasm but were excluded from regions evenly spaced along the germtubes, as was CotA, in a manner suggesting such shadows represent nuclei (Figure 3D–G). Consistent with this, the regions negative for GFP fluorescence synchronously doubled in number in a manner consistent with the synchronous mitotic nuclear divisions of *A. nidulans*. During the period of nuclear division, CmkA, SudD^Rio1^, BckA and CotA each entered nuclei before again being excluded to the cytoplasm following nuclear division (Figure 3D–G). The equilibration of these kinases across the nuclear envelope during mitotic entry is due to the partial disassembly of NPCs which increases the permeability of the nuclear envelope and inactivates nuclear transport during mitosis [12], [13], [71] Similar behavior has been reported for other cytoplasmic proteins [72]. The cytoplasmic location of CmkA, SudD^Rio1^ and BckA is consistent with the location of their respective orthologues in *S. cerevisiae* and *S. pombe* [68], [73], [74]. In *S. pombe* the BckA orthologue, Mpk1, additionally locates weakly to septa [75]. Although we did not initially observe BckA at septa, careful inspection revealed that it was occasionally weakly enriched at septa above its cytoplasmic levels suggesting that this location is conserved in *A. nidulans* (Figure 3H).

The DLAP tagged version of the essential IreA kinase, which is predicted to regulate the unfolded protein response [5], displayed a cytoplasmic localization with a structured appearance (Figure 3I). Given that IreA encodes an N-terminal hydrophobic signal sequence and an ER luminal domain similar to other Ire1 kinases [76], it is likely that IreA locates to the ER. Notably however, unlike other *A. nidulans* ER proteins [12], [77], IreA was not obviously located in the outer nuclear membrane which is contiguous with the ER.

#### The A. nidulans TorA kinase locates to vesicles and vacuoles

**Figure 4:**
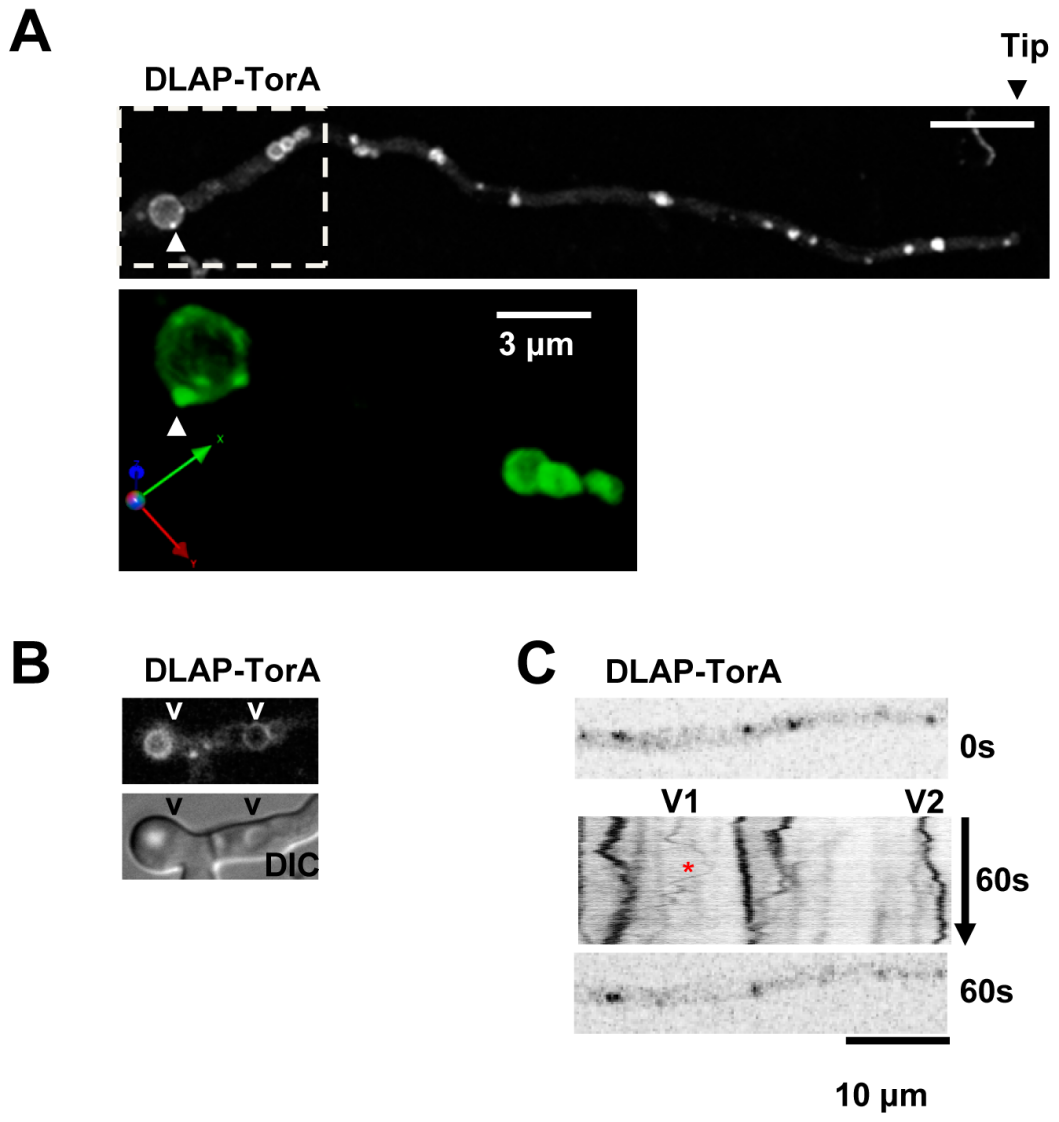
The TorA kinase locates to vacuoles and vesicles. **A** A germling showing the typical pattern of DLAP-TorA location to vesicles along the germtube and vacuoles distal from the cell tip. The lower panel shows a higher magnification of the region shown in the inset rotated in 3D demonstrating bright TorA vesicles (arrowheads) on the outside of the vacuolar membrane. **B** TorA is enriched at circular membranous structures which align with vacuoles (v) visualized by DIC. **C** A kymograph showing bidirectional movement of the TorA vesicles. Data were generated from a single confocal Z slice with images collected every 0.083 sec. Note that compared to larger vesicles (e.g. V2), smaller vesicles move longer bidirectional distances of 2–4 µm (e.g. V1). Bar ∼10 µm.

The DLAP-TorA kinase, predicted to perform multiple functions in the regulation of cell growth, displayed a distinctive localization to vacuolar membranes and vesicles which generally increased in size and abundance in regions distal from the tip (Figure 4A–B). This localization pattern is consistent with TorA locating to the endosomes and vacuoles of the endocytic pathway [78]. In *A. nidulans* endosomes undergo linear bidirectional movements but this motility decreases as endosomes mature [79]. Consistent with this, kymograph representations of time lapse images indicated that large TorA vesicles were relatively immobile whereas smaller vesicles underwent bidirectional movements (Figure 4C). These smaller TorA vesicles moved in one direction for 2–4 µm before stopping and reversing direction resulting in a V-shaped pattern on kymographs that is characteristic of endosomes (Figure 4C) [79]. The rate of movement of these smaller TorA vesicles was in the order of 1.8 µm/sec at 23° which is comparable with the published rate of 2.5 µm/sec at 25° for early endosomes [80]. However, early endosomes often move longer distances of over 15 µm and are more numerous than TorA vesicles suggesting that TorA predominately locates to more mature endosomes. Supporting this, TorA was often apparent at distinct foci in the vicinity of vacuolar membranes and high resolution 3D reconstructions revealed that these foci were adjacent to, or possibly connected to, the outside of the vacuolar membrane (Figure 4A arrowheads). Together these data suggest that at least some TorA vesicles correspond to late endosomes which fuse to the vacuolar membrane to which TorA also locates.

Tor kinases also locate to endomembrane systems in other organisms and their function at these locations is of considerable interest [81]–[86]. In *S. cerevisiae* one of the two Tor kinases, Tor1p, resides at vacuolar membranes [81]. In mammalian cells inactive mTORC1 is recruited to lysosomes, the mammalian equivalent of vacuoles, in response to high levels of amino acids [83]. This facilitates mTor activation at lysosomes which regulates increased protein synthesis when amino acids are abundant. The location of TorA to vesicles and vacuoles in *A. nidulans* suggests that regulation of TorA activity at these endomembranes is likely important for the cellular response to amino acids and other nutrients in filamentous fungi.

### Nuclear Kinases which Disperse throughout the Cell during Mitosis

**Figure 5:**
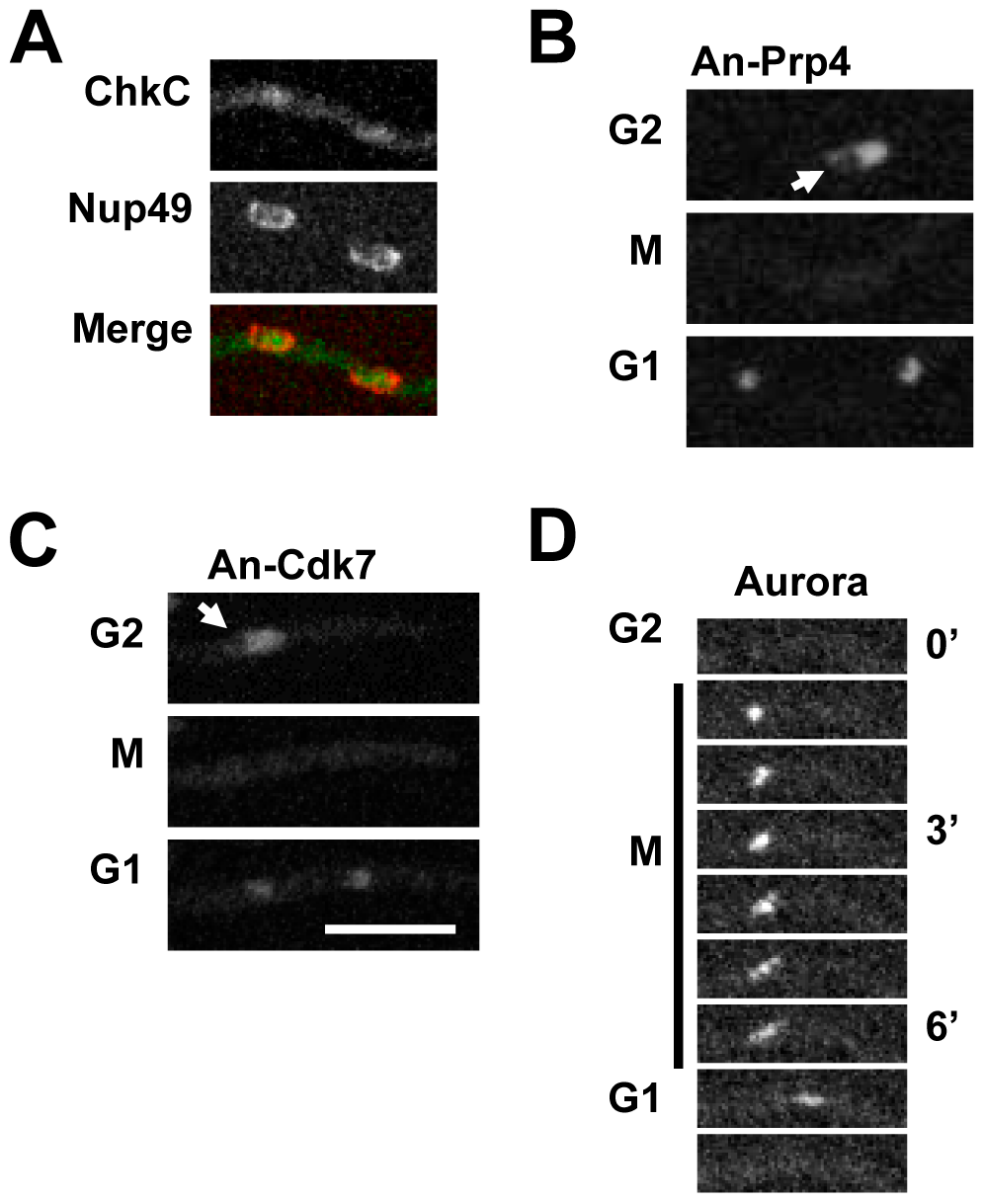
Kinases with a nuclear location. **A** ChkC-DLAP is weakly enriched in nuclei indicated by the Nup49-mCherry nuclear envelope marker. **B and C** An-Cdk7-DLAP and An-Prp4-DLAP are enriched in interphase nuclei but disperse from mitotic nuclei during nuclear division. Arrows indicate nuclear regions with low levels of An-Prp4 and An-Cdk7 that are reminiscent of nucleoli. **D** Aurora-DLAP locates to the mitotic apparatus. Bar ∼10 µm.

Although 8 kinases were enriched in nuclei, time lapse imaging indicated distinct patterns of location during the cell cycle including two kinases which dispersed from nuclei during mitosis. Of these, the essential predicted mRNA processing kinase An-Prp4-DLAP was highly enriched in nuclei but was excluded from a nuclear sub-compartment which most likely represents the nucleolus (Figure 5B). To our knowledge this is the first time an endogenously tagged version of the Prp4 kinase has been localized as global localization studies failed to localize *S. pombe* Prp4 whereas *S. cerevisiae* lacks a Prp4 orthologue [73]. Nuclear localization of An-Prp4 was observed throughout the cell cycle apart from during mitosis when An-Prp4 dispersed from nuclei for 7.5+/−0.6 min (n = 4) due to the inactivation of nuclear cytoplasmic transport during mitosis when NPCs partially disassemble [59]. Mitotic dispersal of An-Prp4 also indicates it does not bind to mitotic nuclear structures. Given this, An-Prp4 is unlikely to have a mitotic nuclear function in *A. nidulans* although in HeLa cells Prp4 locates to kinetochores where it has been implicated in mitotic SAC regulation [55].

The An-Cdk7-DLAP kinase, which is essential in *A. nidulans*, was moderately enriched in interphase nuclei in a similar manner to An-Prp4 although its apparent exclusion from the nucleolus was less obvious (Figure 5C). During mitosis An-Cdk7-DLAP dispersed from nuclei indicating it does not bind to mitotic nuclear structures. In *S. pombe* the Mcs6 kinase is orthologous to An-Cdk7 and is also excluded from nucleoli although it is additionally present in cytoplasmic dots [73]. Mcs6 has dual functions in regulating CDK activation as well as RNA pol II transcription [87]. The orthologous *S. cerevisiae* kinase, Kin28, locates to nuclei where it regulates RNA pol II transcription although it is not involved in CDK activation [68].

Contrasting An-Cdk7 and An-Prp4, other nuclear kinases remained associated with nuclear structures during mitosis strongly suggesting they have functions at these mitotic structures.

### Kinases which Locate to Mitotic Nuclear Structures

Seven kinases located to nuclear structures during mitosis including ChkC, a Chk2 related kinase specific to the filamentous ascomycetes whose localization has not previously been reported in any organism. ChkC functions in the cellular response to replicative stress in *A. nidulans* although the orthologous Mus-59 kinase of *N. crassa* functions in response to DNA double strand breaks [5], [88]. Consistent with the role of ChkC/Mus-59 kinases in response to genotoxic stress ChkC-DLAP was weakly enriched in nuclei (Figure 5A). In addition, ChkC displayed a complicated mitotic localization pattern which will be described elsewhere (CDS, SAO, manuscript in preparation). As expected the essential Aurora mitotic kinase displayed a complex pattern of location to the mitotic apparatus which will be described, along with its functional analysis, elsewhere (Figure 5D; CDS, SAO, manuscript in preparation).

#### SldA^Bub1/R1^ associates with SldB^Bub3^ and locates to mitotic kinetochores

**Figure 6:**
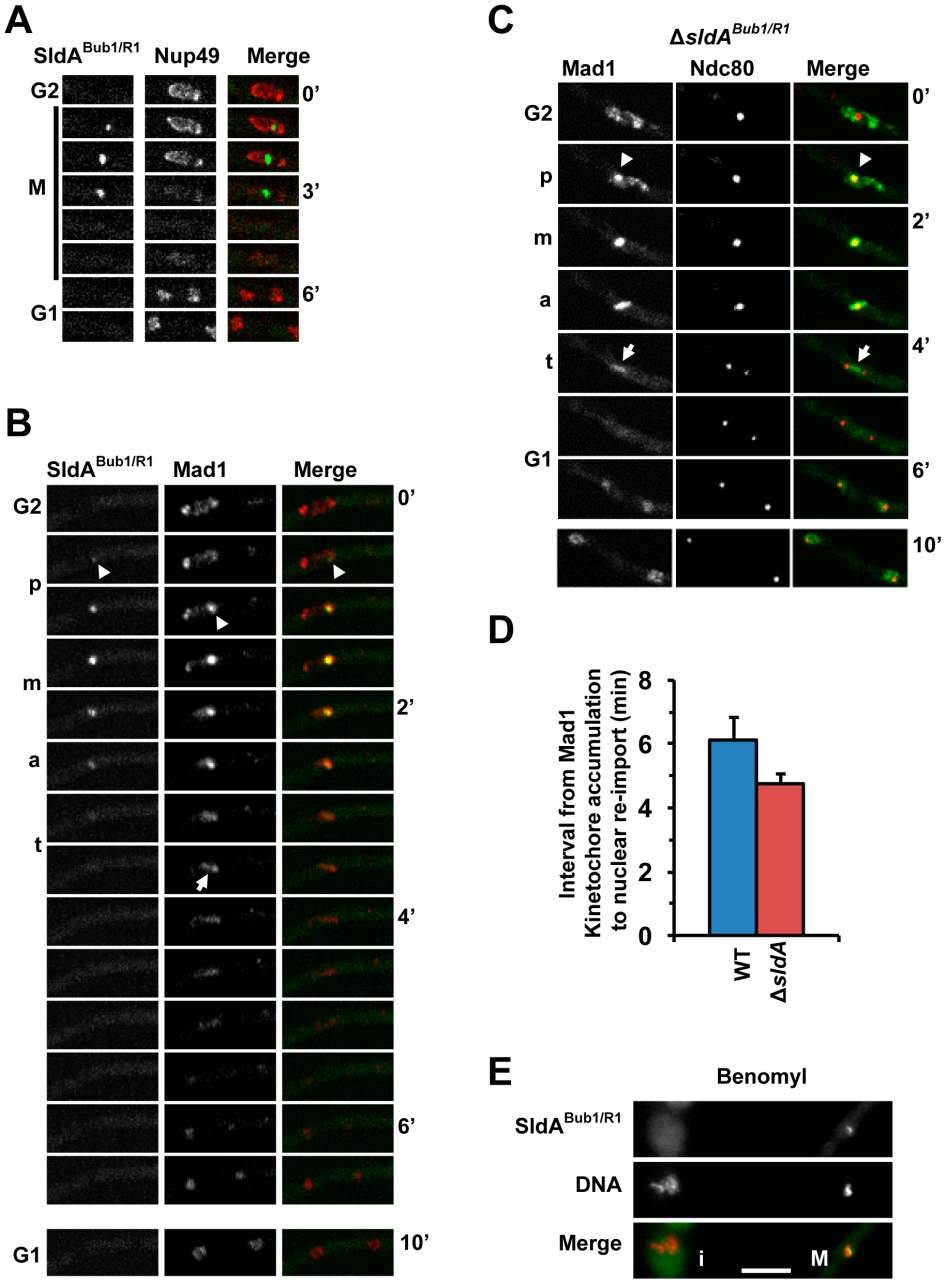
SldA^Bub1/R1^ appears at kinetochores before Mad1 but is not required for Mad1 kinetochore location. **A** Time lapse images of SldA^Bub1/R1^-DLAP together with Nup49-mCherry during mitosis. **B** As for A but with Mad1-mCherry showing that SldA^Bub1/R1^ accumulates at kinetochores (arrowheads) while Mad1 is still at its interphase NPC location. Following anaphase onset, SldA^Bub1/R1^ disperses from kinetochores but Mad1 remains prominent at its spindle matrix location (arrow). p = prophase, m = metaphase, a = anaphase, t = telophase. **C** In a Δ*sldA* ^Bub1/R1^ mutant Mad1-GFP still co-locates with Ndc80-mCherry at kinetochores (arrowhead) during early stages of mitosis and persists at the spindle matrix until telophase (arrow). **D** Graph showing that mitotic duration is shorter in Δ*sldA* ^Bub1/R1^ mutants than in wild type cells. **E** SldA^Bub1/R1^ locates to kinetochores during a mitotic SAC arrest in the presence of benomyl (M) but not in an interphase cell (i). Bar ∼10 µm.

Although SAC function is highly conserved and most organisms encode two related SAC proteins, Bub1 and BubR1/Mad3, many filamentous fungi only encode a single version which is called SldA^Bub1/R1^ in *A. nidulans* [5], [66], [89]. This suggests that SldA^Bub1/R1^ carries out all the functions of the Bub1 and BubR1/Mad3 family members. As shown previously SldA^Bub1/R1^ localizes to kinetochores as cells enter mitosis and is maintained at this location when spindle formation is prevented by benomyl treatment (Figure 6A and E) [59]. Although this localization is characteristic of SAC proteins, SldA^Bub1/R1^ surprisingly accumulated at kinetochores 1.4+/−0.4 min (n = 6) before complete Nup49 dispersal suggesting that its kinetochore recruitment is a very early mitotic event. Given this, we determined if SldA^Bub1/R1^ kinetochore recruitment preceded that of the Mad1 SAC protein. This revealed that SldA^Bub1/R1^ initially accumulated at kinetochores while Mad1-mCherry was still present at its interphase NPC residence and before Mad1 transition to kinetochores and the surrounding spindle matrix (Figure 6B) [90]. Both SldA^Bub1/R1^ and Mad1 then remained at kinetochores until the metaphase to anaphase transition at which point SldA^Bub1/R1^ dispersed throughout the cell while Mad1 persisted at its mitotic spindle matrix location.

The finding that SldA^Bub1/R1^ accumulates at kinetochores earlier than Mad1 suggests that it behaves like mammalian Bub1 which appears at kinetochores very early in prophase and whose activity is required for Mad1 kinetochore recruitment [53]. Given this, we determined if SldA^Bub1/R1^ is required for Mad1 kinetochore location using a recently generated *sldA^Bub1/R1^* deletion mutant [5]. This revealed that Mad1 localized normally to both kinetochores and the spindle matrix in the absence of SldA^Bub1/R1^ (Figure 6C). Interestingly however, the duration of mitosis in *sldA^Bub1/R1^* mutants was significantly shorter than for wild type cells (Figure 6D), as is also the case for *mad1* and *mad2* SAC mutants [90]. Thus, the requirement for SldA^Bub1/R1^ in mitotic timing and SAC function is independent of the kinetochore localization of Mad1.

In addition to 22 SldA^Bub1/R1^ peptides representing 32% sequence coverage, LC-MS/MS analysis of purified SldA^Bub1/R1^-DLAP also identified 6 peptides representing 22% sequence coverage of the SldB^Bub3^ (AN2439) SAC protein. Although SldA^Bub1/R1^ and SldB^Bub3^ were both identified in a genetic screen for mutants synthetically lethal without dynein function [66] and are predicted to interact based on studies in other organisms [91], to our knowledge this is the first demonstration that they co-purify in filamentous fungi. Moreover, supporting the sensitivity of this approach, SldA^Bub1/R1^ is a low abundance kinase and only a weakly silver staining band corresponding to the size of SldB^Bub3^ was present in the SldA^Bub1/R1^ purification (Figure 2).

#### The NimX^Cdk1^ kinase locates to nuclei and mitotic SPBs

**Figure 7:**
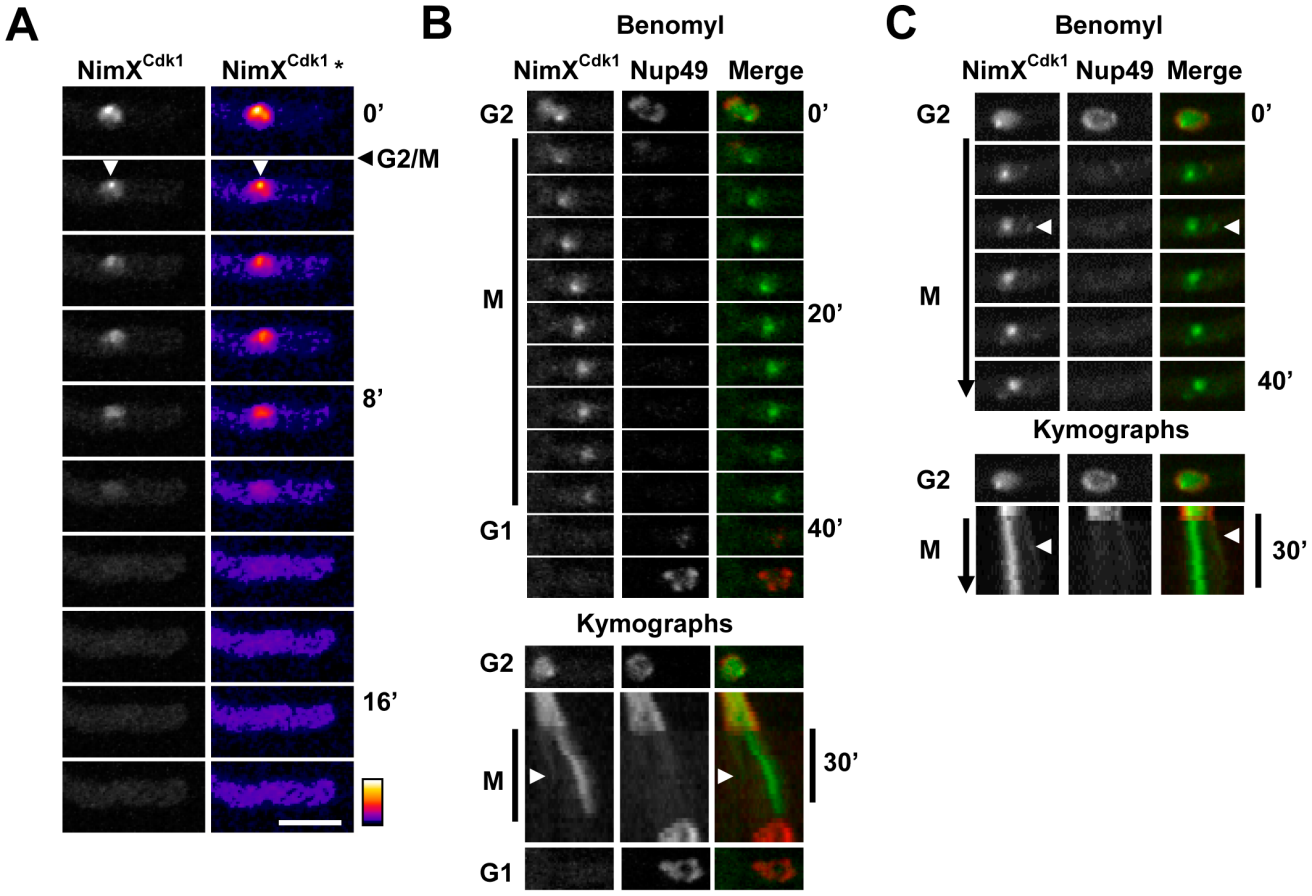
The NimX^Cdk1^ kinase in enriched in nuclei and at SPBs. **A** NimX^Cdk1^-DLAP concentrates to nuclei and SPBs during G2 but partially disperses from nuclei during mitotic entry. False coloring (NimX^Cdk1^ *) more clearly reveals NimX^Cdk1^ location to SPBs (arrowhead) and shows that NimX^Cdk1^ accumulates in the cytoplasm as it is released from nuclei during mitosis. **B and C** Time lapse images and kymographs showing nuclei entering a mitotic SAC arrest in the presence of benomyl. NimX^Cdk1^ remains concentrated around SPBs during SAC arrest. The arrowheads indicate an additional focus of NimX^Cdk1^ present during SAC arrest which appears to be in the vicinity of the nuclear envelope. As shown in B, NimX^Cdk1^ disperses during SIME prior to Nup49 reassembly at NPCs in the nuclear envelope. Bar ∼10 µm.

The NimX^Cdk1^-DLAP cell cycle kinase located as previously reported during interphase and mitosis [92], [93]. During early G1 NimX^Cdk1^ was predominantly cytoplasmic but gradually accumulated in nuclei as cells progressed through interphase. This is because the nuclear location of NimX^Cdk1^ is dependent on its binding partner Cyclin B which is synthesized during interphase but is degraded following anaphase onset [59], [92]–[94]. Also similar to Cyclin B, NimX^Cdk1^ became prominent at the SPBs during G2 (Figure 7A). When cells entered mitosis NimX^Cdk1^ was partially released from nuclei although a pool remained concentrated around the SPBs. As cells progressed through mitosis nuclear levels of NimX^Cdk1^ decreased concomitant with an increase in its cytoplasmic levels as can more clearly be seen in the false colored images (Figure 7A). Interestingly however, although Cyclin B is degraded following metaphase, a recent study indicates that NimX^Cdk1^ dispersal occurs while Cyclin B is still present within nuclei suggesting that NimX^Cdk1^ surprisingly dissociates from Cyclin B during mitosis [92].

We next determined if NimX^Cdk1^ is maintained at SPBs during a mitotic SAC arrest induced by the microtubule poison benomyl. Under these conditions the SAC arrests cells in a prometaphase-like state with unseparated SPBs for ∼45 min before the SAC is inactivated allowing cells to return to interphase without nuclear division in a process termed spindle independent mitotic exit (SIME) [59]. During entry into mitotic SAC arrest NimX^Cdk1^ partially dispersed from nuclei although a pool remained concentrated around the SPBs, as occurs during normal mitotic entry (Figure 7B and C). This SPB associated pool of NimX^Cdk1^ persisted during the mitotic SAC arrest but dispersed during SIME prior to cells returning to interphase as indicated by Nup49 reassembly to the nuclear envelope (Figure 7B). In addition to SPBs, Cyclin B is also present at other foci associated with the nuclear periphery during SAC arrest [59]. Importantly, NimX^Cdk1^ also located to similar foci at the nuclear periphery during SAC arrest suggesting that these foci represent active pools of NimX^Cdk1^/Cyclin B (Figure 7B and C arrowheads). As NimX^Cdk1^/Cyclin B is a major mitotic regulator it will be of interest to determine the nature of these apparently active pools of NimX^Cdk1^/Cyclin B associated with the nuclear envelope when cells are arrested in mitosis. One interesting possibility is that they coincide with clusters of partially disassembled NPCs present during SAC arrest [12].

As expected, LC-MS/MS analysis of affinity purified NimX^Cdk1^-DLAP identified NimE^Cyclin B^ (AN3648) with 9 peptides representing 21% sequence coverage as well as 71% sequence coverage for NimX^Cdk1^ (Table 1). Although partially obscured by the 67 kD NimX^Cdk1^-DLAP chimera, a band likely corresponding to the 55 kD NimE^Cyclin B^ protein was present in the NimX^Cdk1^ purification (Figure 2). Interestingly, in addition to NimE^Cyclin B^, the AN3795 putative B-type cyclin was present in the NimX^Cdk1^ purification but was not in the other 9 kinase purifications. Although sequence coverage was low, AN3795 was identified in two independent NimX^Cdk1^-DLAP purifications, indicating that this interaction is reproducible. We have called AN3795 PucA as it is related to the fission yeast Puc1 cyclin which associates with Cdk1 to regulate the G1/S transition [95]. Together, this suggests the PucA is a second cyclin which associates with NimX^Cdk1^ possibly to regulate the G1/S transition.

#### The An-Cdc7 kinase locates to interphase nuclei and mitotic SPBs

**Figure 8:**
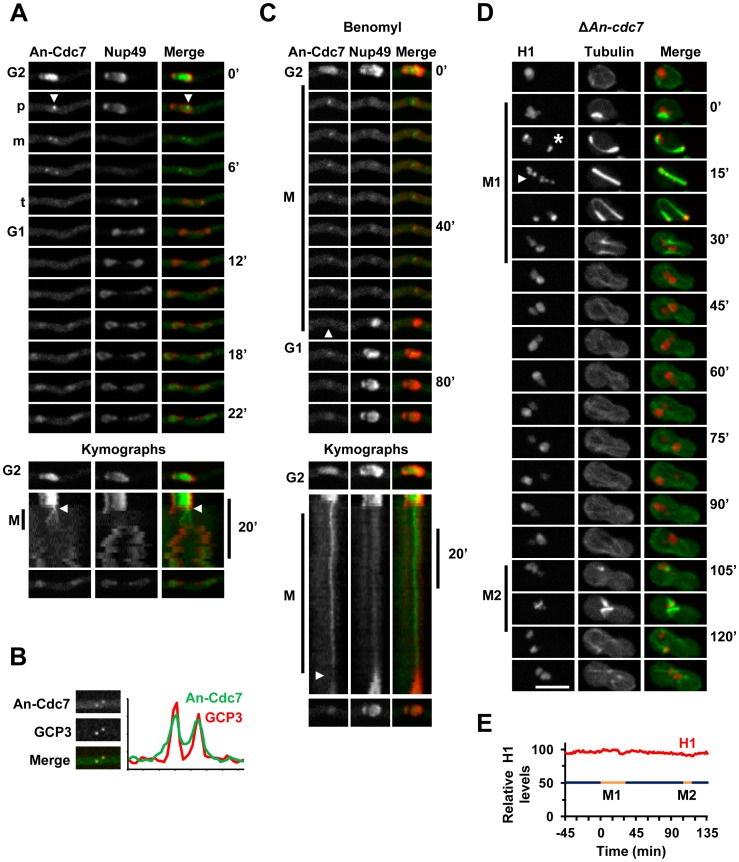
The An-Cdc7 kinase is predominantly nuclear during interphase and locates to mitotic SPBs. **A** Time lapse images and kymographs of the nucleus shown in Video S1 showing that An-Cdc7-DLAP is enriched in interphase nuclei defined by Nup49-mCherry. As Nup49 disassembles from the nuclear envelope in prophase (p) nuclear An-Cdc7 disperses apart from a small focus (arrowhead) which segregates during metaphase (m) before dispersing prior to Nup49 reassembly to the daughter nuclei in telophase (t). As shown in the kymographs An-Cdc7 begins to accumulate in nuclei 8 min after Nup49 reassembly although some remains in the cytoplasm. **B** Images and pixel line intensity profile showing An-Cdc7 co-localization with GCP3-mCherry at SPBs during metaphase. **C** Transit through a mitotic SAC arrest in the presence of benomyl. An-Cdc7 remains at SPBs during mitotic SAC arrest but disperses (arrowheads) during SIME. **D** Time lapse images of the cell in Video S2 showing histone H1-mCherry and GFP-tubulin in a cell lacking An-Cdc7. Uninucleate conidiospores were germinated at 32° from a heterokaryon in selective media which only allows *ΔAn-cdc7* cells to form germtubes. Although *ΔAn-cdc7* cells do not undergo DNA replication, this cell attempts mitosis twice (M1 and M2) separated by an intervening period of interphase. Note that during M1 (see Figure S3E in Supplementary File S1 for all time points) up to 8 distinct Histone H1 foci (15 min arrowhead), likely representing the unreplicated chromosomes, are apparent along the spindle. This leads to a prolonged mitotic arrest before cells return to interphase forming two uneven masses of DNA. During M2 two spindles form before the cell returns to interphase after a transient mitotic arrest. Bar ∼10 µm. **E** Graph showing the relative levels of histone H1 fluorescence for the cell shown in D. The periods of mitotic arrest (gold) and interphase (blue) are indicated.

Live cell imaging of An-Cdc7-DLAP (called Hsk1 in *S. pombe*) indicated that it was enriched in interphase nuclei, consistent with the well established function of the Cdc7/Dbf4 complex, called DDK for Dbf4/Drf1-dependent kinase, during DNA replication (Figure 8A) [5], [96], [97]. As cells entered mitosis the majority of An-Cdc7 dispersed from nuclei although a small pool surprisingly localized to a single nuclear focus (Figure 8A, arrowheads; Video S1) which segregated into two foci in a manner identical to mitotic SPBs. Confirming this, An-Cdc7 co-localized with the GCP3 SPB marker [98] as the spindle poles separated (Figure 8B and Figure S3A in Supplementary File S1). By telophase, when Nup49 began to reassemble to the nuclear envelope, An-Cdc7 had dispersed from the SPBs (Figure 8A; Video S1). Although *A. nidulans* SPBs and kinetochores cluster together during interphase, the SPB location of An-Cdc7 during mitosis was distinct from segregating kinetochores visualized by the Ndc80-mCherry kinetochore marker (Figure S3B in Supplementary File S1). In *S. cerevisiae* Cdc7p has recently been shown to locate to kinetochores from telophase to early G1 [99]. Our analysis indicates An-Cdc7 does not appear to locate to kinetochores during their mitotic segregation. However, because kinetochores cluster at SPBs during interphase and then at telophase we cannot exclude the possibility that An-Cdc7 has some more direct interactions with kinetochores/centromeres during the early and late stages of mitosis.

Following mitosis, An-Cdc7 began to reaccumulate in G1 nuclei 6.8+/−0.7 min (n = 12) after NPC reassembly which reestablishes nuclear transport (Figure 8A). However during early G1 An-Cdc7 had not fully accumulated in nuclei and some was still present in the cytoplasm (Figure 8A). This initial delay and then slow nuclear accumulation of An-Cdc7 in G1 indicates that its post-mitotic nuclear import has an additional level of regulation. In *S. cerevisiae* Cdc7p nuclear accumulation requires re-synthesis of its binding partner Dbf4p which is degraded following mitosis [100], [101]. Given this it is likely that a similar level of regulation exists in *A. nidulans* whereby post-mitotic synthesis of NimO^Dbf4^ would be required for An-Cdc7 nuclear import.

We next determined if An-Cdc7 mitotic location was dependent upon microtubules by treating cells with benomyl. Microtubule depolymerization did not affect An-Cdc7 location to interphase nuclei and when these cells entered mitosis a pool of An-Cdc7 persisted at the SPBs (Figure 8C). Importantly, An-Cdc7 remained associated with the unseparated SPBs throughout the mitotic SAC arrest before dispersing during SIME (Figure 8C). Thus An-Cdc7 associates with SPBs independently of microtubules and SAC activation maintains An-Cdc7 location at SPBs.

Although *An-cdc7* is essential we have recently utilized heterokaryon rescue to study the phenotype of the null allele [5]. As shown for DDK mutants in other systems and for NimO^Dbf4^ in *A. nidulans* [96], [97], *An-cdc7* mutants do not undergo DNA replication but still attempt mitosis due to a failure to activate the checkpoint which prevents mitotic entry when DNA replication is incomplete [5]. In this scenario, SAC activation would be expected upon mitotic entry as the absence of sister chromatids would prevent biorientation of chromosomes. It is known that in the absence of spindle function *A. nidulans* cells still undergo multiple cell cycles, each consisting of a round of DNA replication and a delayed failed mitosis [59]. Given this we were interested if in *An-cdc7* mutants multiple cell cycles could occur in the absence of both DNA replication and successful mitosis. To determine this we utilized a heterokaryon containing both wild type and *ΔAn-cdc7* nuclei as well as endogenously tagged GFP-tubulin and histone-H1-mCherry [5]. Uninucleate *ΔAn-cdc7* spores generated from this heterokaryon were identified by their ability to form germtubes on selective media whereas *An-cdc7* wild type cells from the same heterokaryon did not form germtubes. During interphase histone H1-mCherry fluorescence levels increased in wild type cells but not in *ΔAn-cdc7* cells, consistent with the expected failure in DNA replication in cells lacking *An-cdc7* function (Figure S3C in Supplementary File S1). Unlike wild type cells which transited mitosis normally, *ΔAn-cdc7* cells often displayed highly abnormal curved or bent spindles during a mitosis which was on average over 4 times the length of wild type mitosis (Figure S3D in Supplementary File S1; Video S2). This is consistent with the SAC delaying mitotic exit before being inactivated allowing cells to return to interphase via SIME. Following one cell cycle in which both DNA replication and mitosis failed, *ΔAn-cdc7* cells continued through the next cell cycle once again failing both DNA replication and mitosis (Figure 8D and E). Notably however, although DNA replication failed in these *ΔAn-cdc7* cell cycles, *ΔAn-cdc7* cells still apparently underwent SPB duplication as multiple spindles formed during the second attempted mitosis (Figure 8D).

Interestingly during mitotic SAC arrest in *ΔAn-cdc7* cells chromosomes often moved along the spindle in between the two spindle poles. For example in the cell shown in Figures 8D and Video S2 two uneven masses of chromosomes are present at the spindle poles early in the mitotic arrest (asterisk) but later in the arrest what appear to be the 8 unreplicated chromosomes spread along the spindle appear (arrowhead). This cell subsequently fails mitosis and returns to interphase generating two unequal masses of chromosomes with different amounts of histone H1 suggesting that random segregation of unreplicated chromosomes had occurred (Figure 8D). In other instances *ΔAn-cdc7* cells transited the mitotic arrest and returned to interphase generating a single mass of chromosomes (Figure S3F in Supplementary File S1).

To our knowledge Cdc7/Hsk1 orthologues have not previously been reported at mitotic SPBs or centrosomes although a global analysis of protein localization in *S. pombe* identified a pool of the Hsk1 binding partner Dfp1 at SPBs [73]. Our finding that An-Cdc7 is maintained at mitotic SPBs suggests that it has a function at this locale in addition to its well established functions during DNA replication. It is noteworthy that other proteins previously thought to function only during DNA replication, including mammalian ORCs (origin replication complex) and MCMs (mini-chromosome maintenance), locate to centrosomes [102]–[104]. Moreover, Orc1 has been implicated in restricting centrosome duplication to one round each cell cycle [104]. Given this it is interesting that loss of *A. nidulans* NimQ^MCM2^ function leads to extranumerary SPBs [105] and that our findings indicate that in *ΔAn-cdc7* mutants SPB duplication is also apparently uncoupled from DNA replication.

The location of An-Cdc7 to SPBs might also be related to the function of the DDK complex in regulating centromeric cohesion [99], [106], [107]. In *S. pombe* DDK phosphorylates Swi6 (also called HP1 for heterochromatin protein 1) which helps establish centromeric cohesion [107]. In this regard it is interesting that a pool of *S. pombe* Swi6/HP1 locates to SPBs [73] and that the orthologous *A. nidulans* HepA^HP1^ (AN1905) also locates to SPBs (SAO, K-FS unpublished) [108]. Thus one interesting model is that An-Cdc7 regulates HepA^HP1^ at SPBs to help maintain centromeric cohesion until anaphase.

Another potential substrate of An-Cdc7, the PlkA^Polo^ kinase, also locates to mitotic SPBs in *A. nidulans* [109]. In *S. cerevisiae* DDK binds to the Cdc5p Polo kinase inhibiting the ability of Polo to promote mitotic exit [110]. It is therefore possible that An-Cdc7 also regulates PlkA^Polo^ at SPBs during mitosis in *A. nidulans*.

#### CkiA^Hrr25^ locates to mitotic spindle pole bodies and septa

**Figure 9:**
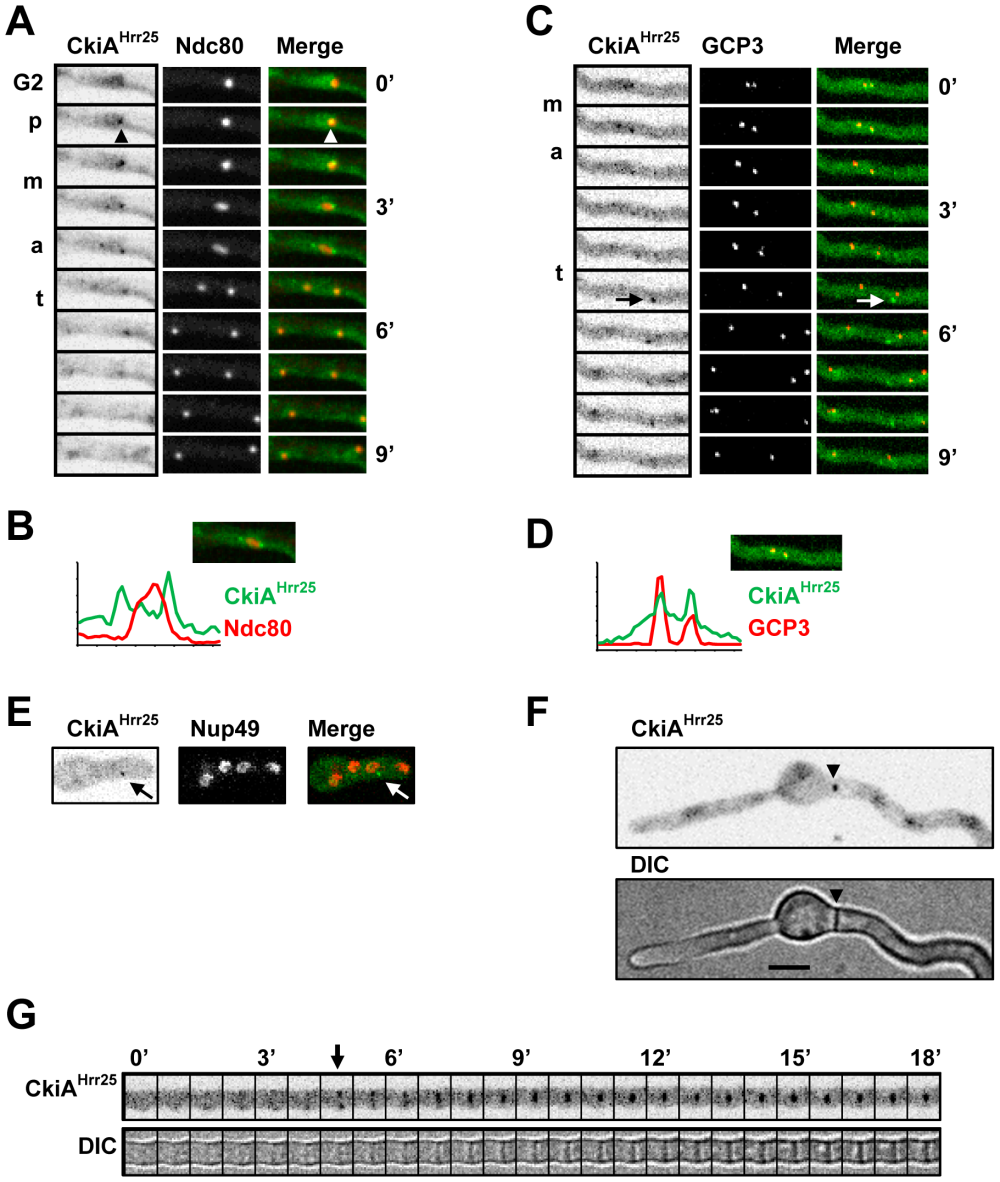
CkiA^Hrr25^ locates to mitotic SPBs and septa. **A** Time lapse images showing CkiA^Hrr25^-DLAP location in comparison with the Ndc80-mCherry kinetochore marker. Grayscale CkiA^Hrr25^ images have been inverted to more clearly show its location. CkiA^Hrr25^ is weakly enriched in the G2 nucleus but concentrates to a single nuclear focus in the region of the kinetochores during prophase (p, arrowhead). Although CkiA^Hrr25^ segregates into two foci during metaphase (m) and anaphase (a) this location is distinct from the kinetochores. **B** Pixel line intensity profile showing that CkiA^Hrr25^ location is distinct from the Ndc80 kinetochores during anaphase. **C** Time lapse images showing that CkiA^Hrr25^ segregates with the GCP3-mCherry SPB marker. The arrow indicates a non-SPB focus which appears during mitosis in some cells and persists into interphase. **D** Pixel line intensity profile showing that CkiA^Hrr25^ co-locates with GCP3 at SPBs. **E** A telophase cell showing a cytoplasmic CkiA^Hrr25^ focus. **F** CkiA^Hrr25^ locates to septa (arrowhead). **G** Time lapse images every 45 s showing that CkiA^Hrr25^ first appears near the cell wall (arrow) as the septum becomes visible by DIC before contracting to a single focus in the center of the septum. Bar ∼10 µm.

The essential CkiA^Hrr25^-DLAP casein kinase 1 (CK1) located to multiple cell structures consistent with the diverse functions of this kinase family including roles in trafficking amino acid transporters to the plasma membrane in *A. nidulans* [5], [111]–[114]. As recently reported, CkiA^Hrr25^ was weakly enriched in interphase nuclei [111] but high resolution confocal time lapse imaging revealed other cell cycle specific locations (Figure 9). As cells entered mitosis CkiA^Hrr25^ concentrated to a single nuclear focus which subsequently segregated into two foci (Figure 9A–D). Comparison with Ndc80-mCherry and GCP3-mCherry indicated that CkiA^Hrr25^ segregated with mitotic SPBs and was not present at segregating kinetochores (Figure 9A–D). In longer asynchronous populations of germlings CkiA^Hrr25^ was also present at 89.4% (n = 121) of septa (Figure 9F). CkiA^Hrr25^ first appeared at forming septa approximately 20 min following mitosis in the same period when the septal cell wall first became visible (Figure 9G). At this time CkiA^Hrr25^ was transiently present near the plasma membrane or as a band in the vicinity of the septal wall before concentrating to a single focus in the septal pore region where it remained for an extended period (Figure 9G). These data suggest that CkiA^Hrr25^ initially locates in the region of the forming septal wall before concentrating at the septal pore. As discussed below, CkiA^Hrr25^ was also occasionally observed at undefined cytoplasmic foci which generally appeared during mitosis or early interphase (Figure 9C and E arrows).

**Figure 10:**
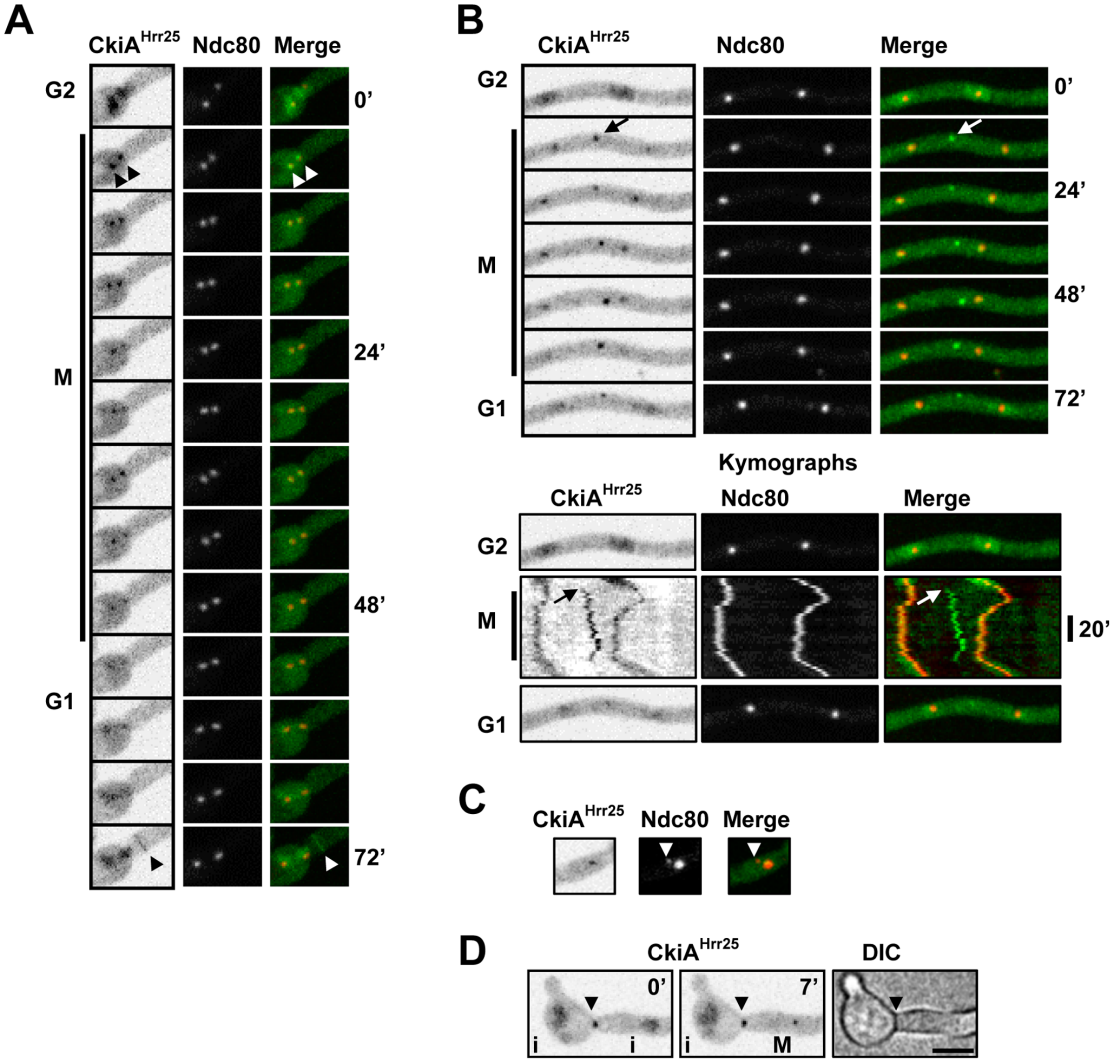
CkiA^Hrr25^ locates to mitotic SPBs and to septa independently of microtubules in benomyl treated cells. **A** Time lapse images showing that CkiA^Hrr25^-DLAP locates to the SPB/kinetochore region (arrowheads) independently of microtubules. Following transit through the mitotic state CkiA^Hrr25^ locates to a forming septum (arrowhead 72′). **B** Time lapse images and kymographs from Video S3 showing a non-SPB CkiA^Hrr25^ focus (arrows) which appears during the SAC arrest and persists into interphase. **C** A nucleus during SAC arrest in which an individual kinetochore (arrowhead) is detached from the SPB/kinetochore cluster. CkiA^Hrr25^ is present at the SPB/kinetochore cluster but not at the unattached kinetochore. **D** A germling in the presence of benomyl with CkiA^Hrr25^ locating to a mature septum (arrowhead). At 0′ the cells on either side of the septum are in interphase (i) and CkiA^Hrr25^ is enriched in nuclei. CkiA^Hrr25^ remains at the septum at 7′ when the cell on the right has entered a mitotic SAC arrest (M) indicated by CkiA^Hrr25^ SPB location. Bar ∼10 µm.

We next examined CkiA^Hrr25^ localization in the presence of benomyl to determine if microtubules were required for its location to SPBs and/or septa, structures which both act as microtubule organizing centers in *A. nidulans* [115]. During mitotic entry without microtubules CkiA^Hrr25^ concentrated at SPBs in a manner similar to a normal mitosis and then remained at the unsegregated SPBs during the ensuing mitotic SAC arrest (Figure 10A and B). Similarly microtubule depolymerization did not obviously affect CkiA^Hrr25^ location to either forming septa or septal pores during either interphase or mitotic arrest (Figure 10A and D). Thus, CkiA^Hrr25^ locates to mitotic SPBs and septa independently of microtubules and is maintained at SPBs during a mitotic SAC arrest. Notably, undefined cytoplasmic CkiA^Hrr25^ foci similar to those observed during normal mitosis were often particularly prominent during mitotic SAC arrest (Figure 10B arrows, Video S3).

In human cells human CK1δ locates to centrosomes and has been implicated in mediating neurite outgrowth in neural cells [116] and centrosome positioning during T-cell activation by regulating microtubule dynamics [117]. Given this the location of CkiA^Hrr25^ to SPBs and septa, might reflect its potential involvement in regulating microtubule dynamics at these structures. In the yeasts *S. pombe* and *S. cerevisiae* CkiA^Hrr25^ orthologues also locate to SPBs and septa/bud neck regions [112], [114], [118]. Notably the two *S. pombe* casein 1 kinases, Hhp1 and Hhp2, are maintained at SPBs during SAC arrest in a manner similar to CkiA^Hrr25^ and have recently been shown to function in a checkpoint which delays cytokinesis in response to mitotic stress [114]. Given this the temporal location of CkiA^Hrr25^ to mitotic SPBs and then to forming septa might similarly help coordinate the completion of mitosis with septation in *A. nidulans*. A potential function for CkiA^Hrr25^ in negatively regulating septation might also be important to prevent sealing of the septal wall such that the septal pore can form.

In budding yeast Hrr25p regulates meiosis I when it is enriched at centromeric regions as part of the monopolin protein complex [113]. While our localization studies indicate that CkiA^Hrr25^ is not enriched at mitotic centromeres, it is possible that SPBs act as a launching pad for CkiA^Hrr25^ to transition to kinetochores during meiosis I. Although highly speculative, such a transition might also involve SPB associated An-Cdc7 (Figure 8), because DDK promotes the association of Monopolin with kinetochores during meiosis I in *S. cerevisiae* [119].

Intriguingly, in addition to nuclei, SPBs and septa, CkiA^Hrr25^ also occasionally located to undefined cytoplasmic foci which were most prevalent during mitosis or mitotic arrest but also persisted for a period during the subsequent interphase (Figure 9C and E, arrows, Figure 10B; Video S3). These foci did not correspond to septa, the SPB/kinetochore cluster or to individual kinetochores which occasionally dissociate from the SPB/kinetochore cluster when spindles cannot form (Figure 10B and C). In addition although these cytoplasmic CkiA^Hrr25^ foci were present during the period between mitosis and septation, they did not locate to sites where septa subsequently formed (data not shown). However, as indicated by the location of the type II myosin MyoB, septal precursors can begin to form at one site and then disassemble and reassemble at another site along hyphae [120]. Given this, and the potential that CkiA^Hrr25^ negatively regulates septation, one possibility is that cytoplasmic CkiA^Hrr25^ foci mark sites of septation which are subsequently aborted, perhaps due to the activity of CkiA^Hrr25^.

#### SepH concentrates in the basal region of apical cells where it displays a biphasic location to SPBs during mitosis and then during septation

**Figure 11:**
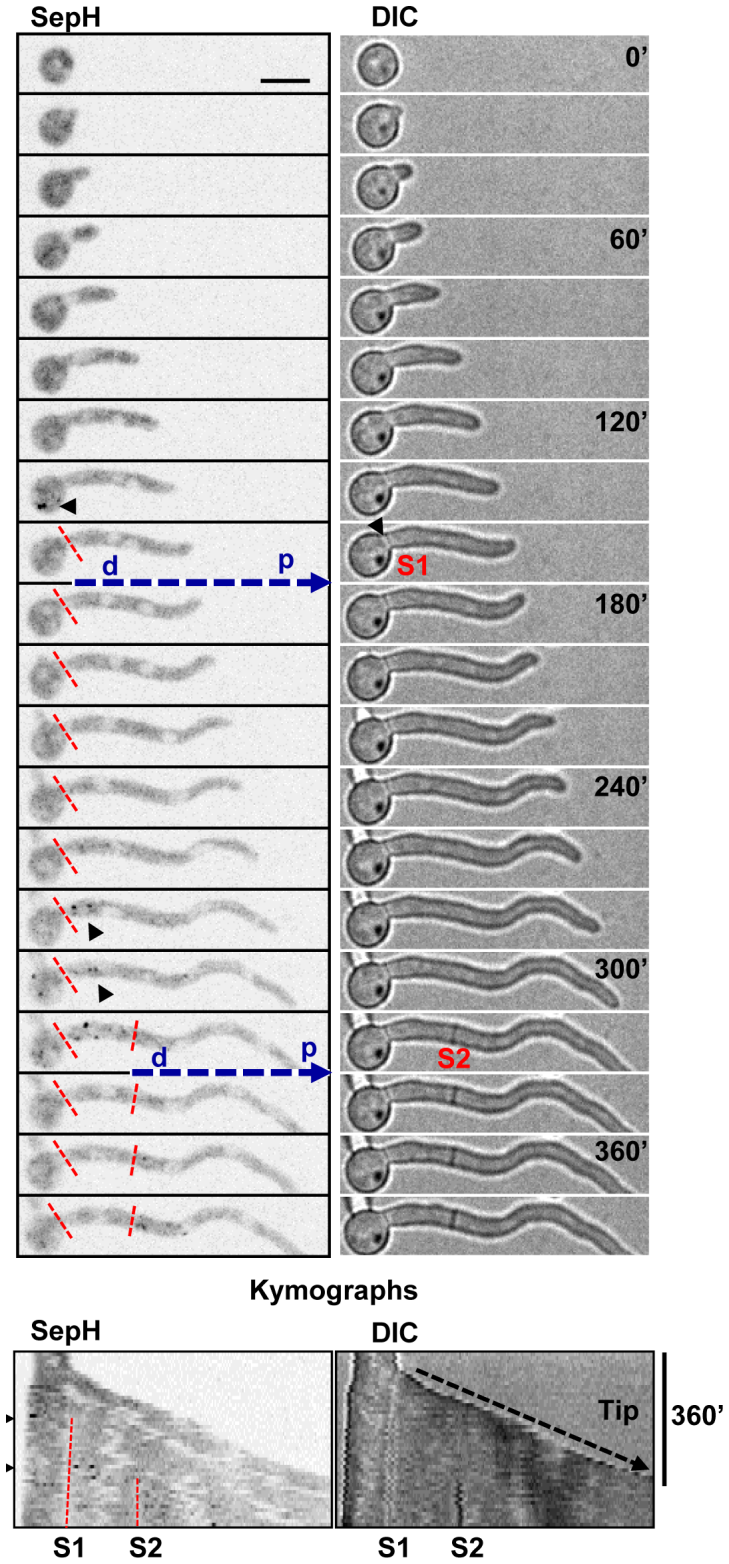
SepH periodically appears at foci distal from the cell tip during growth. Time lapse images and kymographs showing SepH-DLAP and DIC to indicate septa formation. The grayscale has been inverted to more clearly visualize the SepH foci (arrowheads) which are prominent in the periods preceding septa formation (S1 and S2). Septa are indicated by broken red lines in the SepH images. The broken blue arrow indicates the distal (d) and proximal (p) end of the new apical cell formed following septation. The kymographs generated from images captured every 10 min depict polarized cell growth, septum formation and the periodic appearance of SepH at foci. Following septation, SepH foci are rarely present in the subapical cell but are present in the basal regions of the apical cell. Bar ∼10 µm.

The SepH-DLAP septation kinase was difficult to detect in short cells but periodically became prominent at foci as cells grew longer. These SepH foci were unevenly distributed along germtubes typically concentrating in regions near the spore bulb of unseptated cells and/or near mature septa (Figure 11). This resulted in an asymmetric distribution of SepH foci concentrated in basal regions distal from the cell tip of longer cells. Time lapse imaging indicated that SepH foci were particularly prominent during periods preceding septation (Figure 11, arrowheads). Following septation, SepH foci periodically concentrated in the basal region of the newly formed apical cell. This results in a shift in the concentration of SepH foci along germtubes as cells grow and septate (Figure 11). These experiments also revealed that the number and intensity of SepH foci varied as cells progressed through the cell cycle.

**Figure 12:**
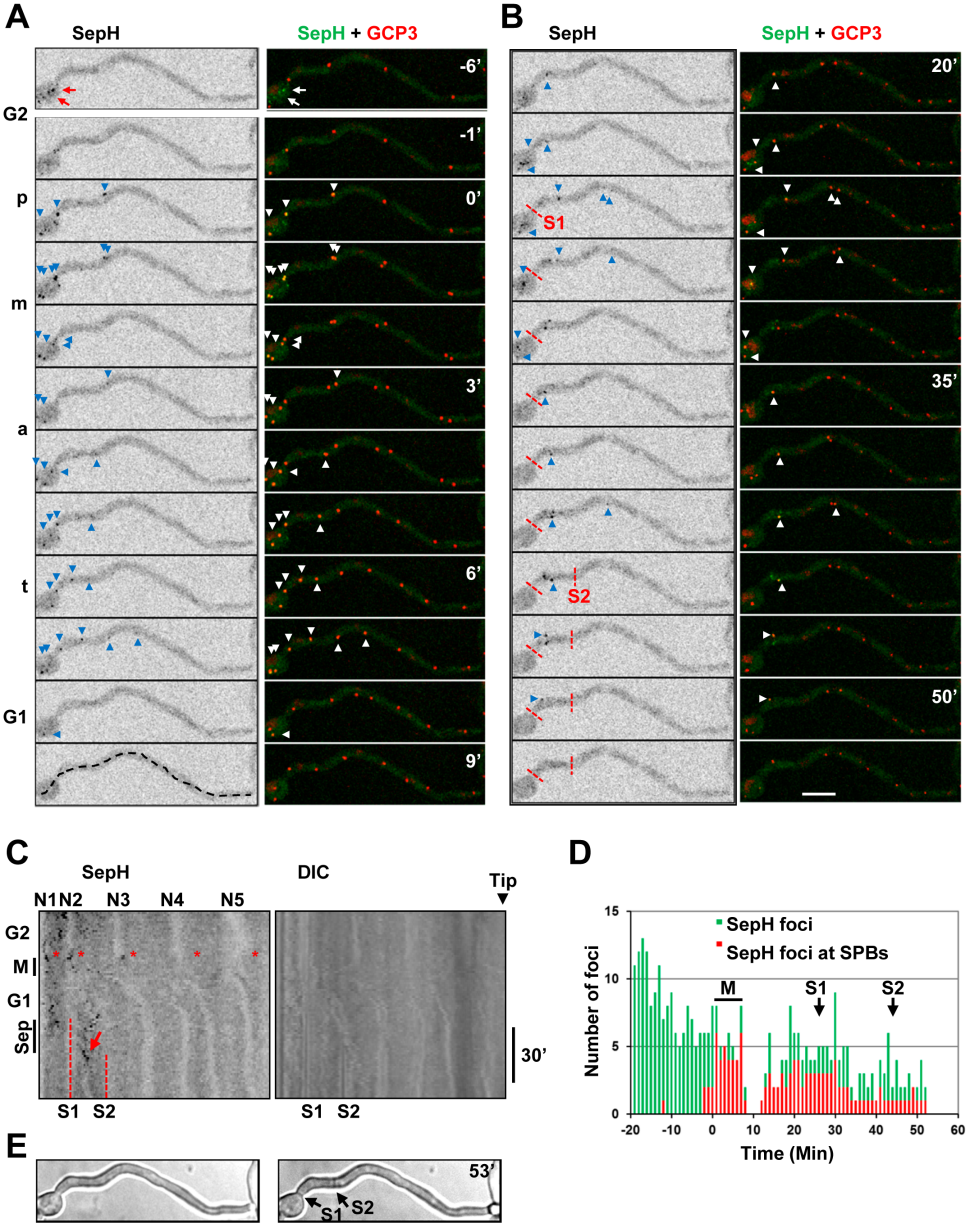
SepH displays a biphasic location to SPBs distal from the cell tip during mitosis and septation. **A** Time lapse images of a germling transiting mitosis for the cell shown in Video S4. Shown is the location of SepH-DLAP together with GCP3-mCherry. SepH positive SPBs are indicated by arrowheads. In G2 over 10 non-SPB SepH foci are present in the region most distal from the cell tip (e.g. arrows, time −6′). As the cell enters prophase (p) SepH associates with tip distal SPBs and is present at SPBs in this region during metaphase (m), anaphase (a) and telophase (t). In early G1 SepH rarely locates to SPBs. The segmented line at 9 min was used to generate the kymographs in C. **B** The same cell in A later in G1 when 2 septa form showing that SepH again associates with SPBs distal from the cell tip in the period preceding formation of the first septa (S1) and the second septa (S2). **C** Kymographs generated from images captured every 90 sec from the same experiment in A and B. The position of nuclei (N1–5) is apparent from the shadows formed by the nuclear exclusion of SepH. The asterisks indicate mitotic entry when SepH enters nuclei and associates with SPBs distal from the cell tip. In early G1 few SepH foci are apparent but SepH again associates with tip distal SPBs preceding the period of septation (Sep). Note that SepH preferentially locates to SPBs on the tip distal side of where the septum subsequently forms (e.g. arrow). **D** Graph generated from the same experiment showing the total number of SepH foci present at each time point (green) and also indicating which of these foci correspond to SPBs (red). **E** DIC images from the start and end of the time course with the position of the septa indicated. Bar ∼10 µm.

SepH is orthologous to the *S. pombe* Cdc7 septation kinase (distinct from *S. cerevisiae* Cdc7p or An-Cdc7) which is the most upstream of the three septation initiation network (SIN) kinases, orthologues of which are required for septation in *A. nidulans* [5], [25]–[27]. Given that *S. pombe* Cdc7 locates to mitotic SPBs [121], [122] we determined if any SepH foci co-localized with the SPB marker GCP3. In these experiments mitosis could be defined by the separation of SPBs. Live cell analysis of polarized germlings with at least 4 nuclei revealed that SepH foci where generally most prominent during early G2 when 89% (n = 28) of cells contained multiple SepH foci in the tip distal region which only rarely associated with SPBs (Figure S4 in Supplementary File S1). As cells progressed into late G2 the number of SepH foci decreased and SepH prominently associated with some SPBs as cells entered mitosis. Notably, the association of SepH with SPBs was not uniform with SepH preferentially associating with the SPBs of mitotic nuclei which were distal from the cell tip in 29 of 31 (93.5%) cells examined (Figure 12A, Video S4). Although the intensity in individual nuclei varied, SepH remained associated with SPBs distal from the cell tip at all stages of mitosis. Following mitosis, the number and intensity of SepH foci decreased during early G1 prior to septation which occurred ∼20 min following mitosis (Figure 12). However as cells continued through G1 SepH foci again became prominent in the tip distal region during the period of septation. Comparison with GCP3 indicated that SepH was prominent at SPBs during the period preceding septation in 38 of 39 (97.4%) cells examined (Figure 12B). As occurs during mitosis, SepH only located to SPBs which were distal from the cell tip during the period of septation. This bias in SepH location to SPBs and other foci in regions distal from the cell tip during G2, mitosis and septation is clearly apparent in the kymographs shown in Figure 12C. These kymographs also reveal that SepH is excluded from interphase nuclei (N1–5) as evidenced by shadows which divide from one to two during mitosis allowing nuclei to be tracked and defining foci associated with these nuclei as SPBs. Thus SepH locates to SPBs in a biphasic manner during the cell cycle, first during mitosis and again during the period of septation. In both cases the location of SepH to SPBs is biased such that it only associates with SPBs of nuclei which are distal from the cell tip and is absent from SPBs of nuclei nearer the cell tip.

**Figure 13:**
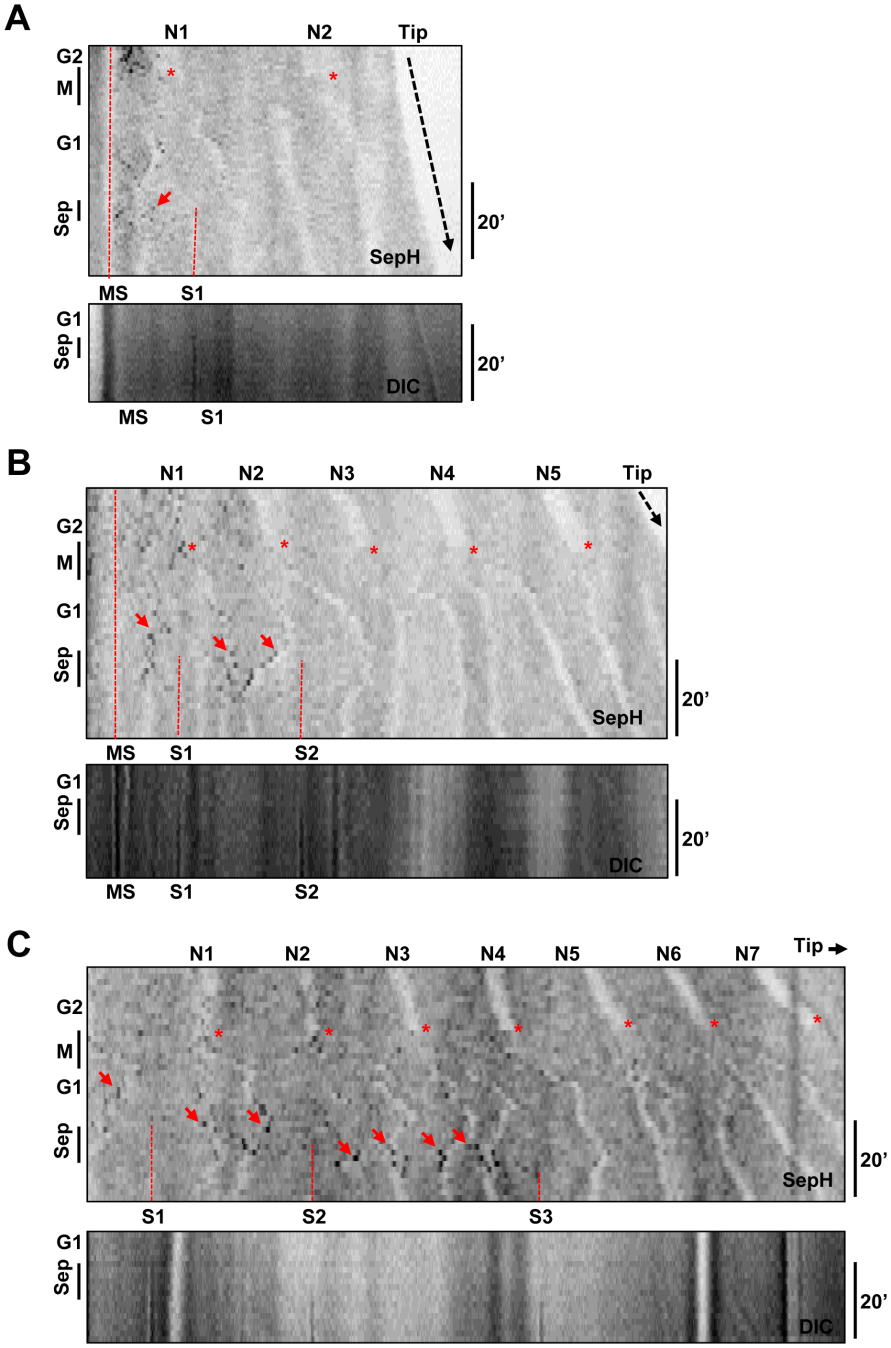
Gradients of SepH location to G1 SPBs shift along germtubes during the formation of multiple septa. Shown are kymographs generated from germlings undergoing mitosis (M) and septation (Sep) with images captured every 1 min. The position of the nuclei (N1–7) is apparent from the shadows formed by the nuclear exclusion of SepH-DLAP. Broken red lines indicate mature septa (MS) or newly forming septa (S1, 2 or 3) which can be seen on the DIC kymographs. The asterisks indicate mitotic entry when SepH enters nuclei but only associates with the SPBs of nuclei distal from the cell tip. Few SepH foci are apparent during early G1 but SepH associates with tip distal SPBs (arrows) prior to the formation of septa. **A** A cell which only forms a single septum. **B** A cell which forms 2 septa. **C** A cell which forms 3 septa. Note that during septation SepH preferentially locates to SPBs (arrows) on the tip distal side of where each septum forms. Following the formation of the last septum SepH disappears from all SPBs.

Although *S. pombe* Cdc7 initially associates with both SPBs during metaphase, during late anaphase and telophase it concentrates to only one SPB and this asymmetric distribution is important for SIN regulation [121]–[125]. We therefore analyzed the localization of SepH and SPBs during the period of septation relative to the position where the septum subsequently formed. During the period preceding septum formation, SepH preferentially concentrated to SPBs on the tip distal side of where the septum subsequently formed in 19 of 20 (95%) cells examined. When cells formed only a single septum SepH dispersed from all SPBs within several minutes of septum formation (n = 9; Figure 13A). Contrasting this, when cells sequentially formed multiple septa, SepH progressively associated with SPBs along the germtube as each septum formed (Figure 12C; Figure 13B and C). For example the cell in Figure 12 sequentially forms 2 septa following mitosis and preceding the formation of each septum SepH concentrates on SPBs on the tip distal side of where each septum subsequently forms. As apparent in the kymograph, this results in a shift in the concentration of SepH following the formation of the first septum such that SepH concentrates in the region between the first septum and where the second septum subsequently forms (Figure 12C arrow). This pattern of SepH localization also occurred when 3 septa formed sequentially along the germtubes of long cells as shown in Figure 13C. In all cases, following formation of the final septum SepH dispersed from all SPBs. Thus during the period of septation SepH concentrates asymmetrically to SPBs on the tip distal side of where each septum subsequently forms and loss of SepH from SPBs correlates with SIN inactivation.

**Figure 14:**
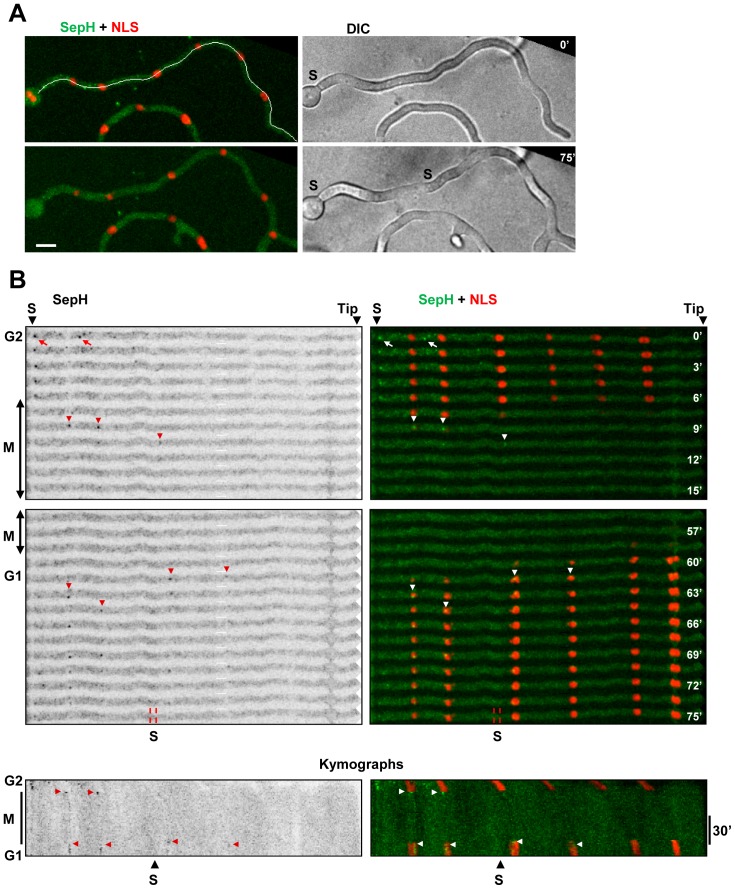
SepH locates to SPBs in a microtubule independent manner and does not spread to tip proximal SPBs when mitotic progression is delayed. **A** A germling before (0′) and after (75′) transit through the mitotic state in the presence of benomyl. A mature septum (MS) is present at the start of the time course while a second septum is beginning to from at the end. SepH-DLAP is shown in green while nuclei are visualized by the NLS-DsRed nuclear transport marker. The cell was digitally straightened along the line in show at 0′ to generate the images shown in B. **B** Time lapse images and kymographs showing mitotic entry, indicated by mitotic NLS-DsRed dispersal, and SIME indicated by re-import of NLS-DsRed. During G2 SepH is at non-SPB foci (arrows) but associates with some SPBs (arrowheads) distal from the cell tip during mitotic entry even though microtubules are depolymerized. During SAC arrest SepH disperses from tip distal SPBs but does not spread to tip proximal SPBs. During SIME SepH again associates with some SPBs that are distal from the cell tip and is apparent at these SPBs until the time of septation. Bar ∼10 µm.

The preferential location of SepH to SPBs distal from the cell tip could be due to the affinity of these SPBs for SepH or potentially because SepH does not have time to spread from tip distal SPBs and foci to tip proximal SPBs during the rapid parasynchronous mitoses of *A. nidulans* germlings. To examine this we followed SepH location during entry into a prolonged mitotic SAC arrest in the absence of microtubules and followed mitotic entry and nuclear position using an NLS-DsRed (nuclear location sequence) reporter construct which disperses from nuclei when cells enter mitosis [71], [126]. Following microtubule depolymerization SepH located to non-SPB foci during G2 as normal (Figure 14B, arrows). As cells entered mitosis SepH re-located to SPBs indicating that the recruitment of SepH to SPBs is independent of microtubules. However, as during normal mitotic entry, SepH only located to SPBs that were distal from the cell tip and did not spread to SPBs proximal to the cell tip even when mitotic progression was delayed (Figure 14B). This asymmetric location of SepH to SPBs distal from the cell tip during entry into such prolonged mitosis occurred in 14 out of 15 germlings examined containing 4 or more nuclei. Thus SepH does not spread to tip proximal SPBs even when mitotic progression is delayed suggesting that tip proximal SPBs have a low affinity for SepH.

**Figure 15:**
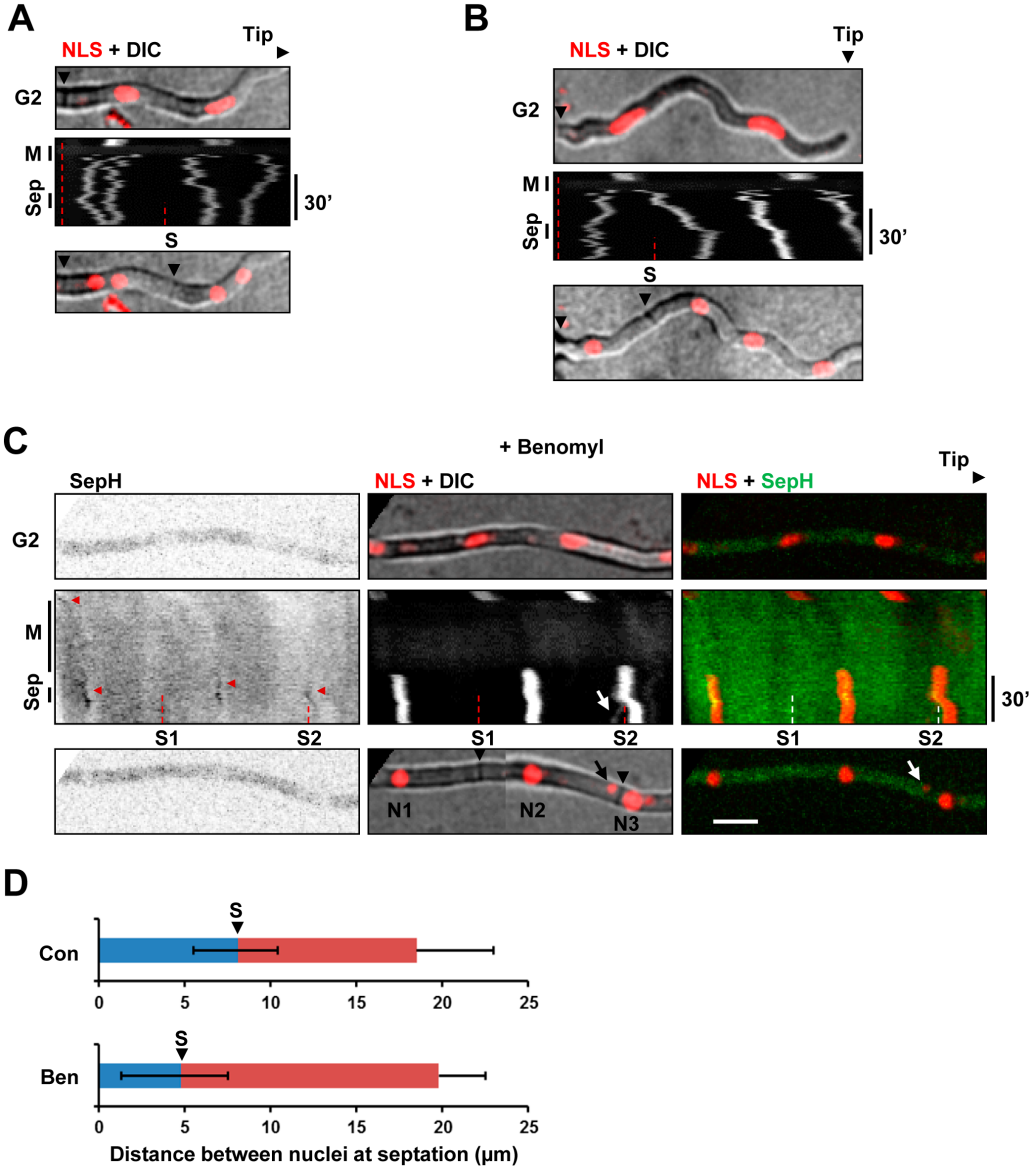
Septal positioning following SIME is abnormal and can sever nuclei. **A** A kymograph showing the NLS-DsRed nuclear transport marker during mitosis and septation (sep). Images of the cell before mitosis (G2) and after mitosis are also shown. A septum (S) forms in between G1 nuclei originating from different parental nuclei. **B** As for A but with the septum forming in the middle of two daughter nuclei generated from the same parental nucleus. **C** Images and kymographs showing transit through the mitotic state without microtubules in a cell which forms two post-mitotic septa. Nuclei are indicated (N1–3). The first septum (S1) forms closer to N2 than N1 whereas the second septum (S2) appears to form directly through N3 resulting in a “cut” phenotype with the septum separating the main nucleus from a smaller nuclear fragment (arrows). As septa formed on different focal planes, the NLS+DIC panel following septation is generated using DIC images from 2 different Z planes each merged with the maximum intensity profile for the NLS. **D** Graphs showing the average distance between nuclei divided by new septa following a normal mitosis (n = 12) and following SIME (n = 11). The distance from the septum to the closest nucleus is shown in blue. The distance from the septum to the furthest nucleus is shown in red. The position of the septum (S) relative to the nuclei it forms in between is indicated. Error bars indicate the standard deviation. Bar ∼10 µm.

The above benomyl experiments also revealed that, unlike NIMX^cdk1^, An-Cdc7 and CkiA^Hrr25^, SepH is not maintained at mitotic SPBs during a mitotic SAC arrest. Notably however, during SIME after the SAC is inactivated, SepH again appeared at SPBs as cells returned to interphase (Figure 14B). Moreover, in 9 out of 10 cells examined SepH was present at SPBs from SIME until septum formation (Figure 14B and 15C). For example in the cell shown in Figure 14B SepH is present at G1 SPBs for at least 15 min at which point the first visible signs of septum formation are apparent. This contrasts what occurs following a normal mitosis when SepH is removed from SPBs in early G1 before re-associating with SPBs during the period of septation (Figure 12).

While performing these experiments we noticed that septa were often aberrantly positioned following SIME often forming adjacent to nuclei. We thus compared the position of septa relative to G1 nuclei following normal mitosis and SIME. Following normal mitosis septa formed between post mitotic nuclei which were 18.5+/−4.5 µm apart (n = 12). In 71% of cells (n = 14) septa formed between daughter nuclei originating from the same parental nucleus (Figure 15B) as occurs in *S. pombe* and has been suggested to be the case in *A. nidulans* [24]. Notably however in 29% of cells septa formed between G1 nuclei originating from different parental nuclei (Figure 15A). In addition, there was no correlation between the position of the mitotic spindle and the position where septa formed in G1. Together these data argue against models proposed for septal positioning based on the position of the mitotic spindle [24], [127]. Following SIME, septa formed between nuclei which were 19.8+/−2.7 µm apart (n = 11), a similar distance to a normal mitosis. Contrasting the situation following a normal mitosis however, following SIME, septa frequently formed significantly closer to one nucleus than the other (Figure 15D). Surprisingly, examination of kymograph representations of nuclear position following SIME indicated that in 4 out of 17 instances septa formed close enough to a nucleus that it caused that nucleus to move. Even more strikingly in two instances a septum appeared to form through a nucleus severing the main part of the nucleus away from a smaller region of the nucleus resulting in a “cut” phenotype (Figure 15C S2). Thus although septal positioning is aberrant in the absence of microtubules septation can still occur.

Septation is best understood in *S. pombe* in which SIN activation can be visualized by Cdc7 association with SPBs [121], [128]. Given this the location of the orthologous SepH kinase to a subset of SPBs during septation provides new insights into how septation is restricted to the basal region of apical cells in *A. nidulans*. During late mitosis *S. pombe* Cdc7 locates to only the new SPB indicating differential SIN activity between the two SPBs [125]. Our data indicate that during septation in *A. nidulans* SepH locates to SPBs distal from the cell tip and suggest that the resulting gradient of SIN activity along the germ tube is important for septal positioning. Supporting this, when cells formed multiple septa the gradient of SIN activity (indicated by the SPB association of SepH) shifted along the germ tube as each septum forms. Therefore although still manifested at SPBs the asymmetric nature of SIN activity in *A. nidulans* occurs along the length of germ tubes and not necessarily between the SPBs of daughter nuclei. Thus the location of SepH during septation potentially explains how septation is restricted to the basal region of apical cells, as well as how such cells can form multiple septa in one cell cycle and how septa can form in between G1 nuclei originating from different parental nuclei.

There are also interesting differences between the locations of *S. pombe* Cdc7 and SepH at other stages in the cell cycle. Unlike *S. pombe* Cdc7, SepH locates to multiple non-SPB foci which are most prominent during early G2 in regions distal from the cell tip. Although the nature of these foci remains to be determined one possibility is that they help concentrate SepH in basal regions of apical cells. It is also notable that SepH prominently associates with a subset of SPBs distal from the cell tip during in early mitosis. It is likely that high NimX^Cdk1^/Cyclin B activity at mitotic SPBs inhibits septation during mitosis in a manner similar to what occurs in *S. pombe* in which Cdc7 first associates with SPBs in metaphase [128], [129]. However, the biphasic location of SepH to SPBs during mitosis and then again during septation suggests that SepH potentially has a mitotic function independent of septation. Further, this mitotic function is likely restricted to early mitosis as SepH is not maintained at mitotic SPBs during a pro-metaphase like SAC arrest. Interestingly SIN kinases are part of networks with other mitotic kinases such as the NIMA related kinase Fin1 in *S. pombe* [125], [130]. Thus we suggest that SepH contributes to early mitotic regulation at SPBs in the basal regions of apical cells in addition to regulating where septation occurs along hyphae during G1.

### Conclusions

We have extended and complemented previous functional analysis of the *A. nidulans* kinome by completing proteomic analysis of 17 *A. nidulans* kinases. The basis for this analysis is the DLAP tag comprised of GFP in tandem with the S-tag affinity purification peptide and developed here for endogenous protein tagging using standard primers and methods. Using this tag we confirmed 3 known or predicted kinase complexes by LC-MS/MS analysis of DLAP tagged kinases purified using a single step affinity purification procedure. Live cell imaging confirmed localizations for 4 previously studied *A. nidulans* kinases and experimentally verified the predicted location of 13 kinases for the first time in filamentous fungi. In addition the An-Cdc7 DNA replication kinase was unexpectedly located to mitotic SPBs suggesting an unknown mitotic function for this kinase. Finally the SepH kinase displayed a biphasic location to SPBs distal from the cell tip during mitosis and then again during septation providing novel insights into how septation is potentially regulated along the length of filamentous fungal hyphae. Collectively the data demonstrate the utility of the DLAP tag for proteomic and localization analysis in *A. nidulans*.

## Materials and Methods

### Generation of Endogenously DLAP Tagged Kinases

Both the N and C-terminal DLAP tags contained 10 amino acid spacers which separated the GFP and S-tag as well as the GFP and the tagged protein. The C-terminal DLAP (GFP-S-tag::*pyrG* ^Af^) cassette was amplified from plasmid pCDS65 (Supplemental File S2) using the standard *A. nidulans* C-terminal tagging primers HP116 and FN01-pyrG [16],[22]. Full length C-terminal tagging constructs were generated by 3 way fusion PCR [6], [22] using primers listed in Supplemental file S4. To endogenously N-terminally tag proteins, a S-tag-GFP DLAP cassette was amplified from plasmid pCDS67 (Supplemental File S3) using primers CDS335 and CDS346, and a *pyrG* ^Af^ cassette amplified from plasmid pCDS60 [5] using primers CDS337 and FN01-*pyrG*. Full length N-terminal tagging constructs were generated by 5 way fusion pcr using primers designed to endogenously express the DLAP-kinase and land the *pyrG* ^Af^ marker between the kinase promoter and the upstream gene. Prior to transformation primers and polymerase were removed using a Qiagen PCR purification kit. The Expand Long Template PCR kit with the buffer 3 system (Roche) was used for fragment amplification, fusion PCR and diagnostic PCR [22].

Tagging constructs were transformed into strain SO451 (*pyrG*89; *wA*3; *argB*2*; ΔnkuA^ku70^::argB pyroA*4; *sE*15 *nirA*14 *chaA*1 *fwA*1) in which the deletion of the *nkuA^ku70^* gene facilitates a high frequency of homologous recombination [7]. Transformed protoplasts were plated onto YAG media lacking uridine and uracil for *pyrG* ^Af^ marker selection and containing 1 M sucrose to maintain osmotic stability. Transformants were streaked to single colony and tested for site specific integration by diagnostic PCR using primers situated external to the targeting sequence for each tagging construct. External primers for C-terminally tagged kinases were the same as those used to confirm correct integration of kinase deletion constructs [5]. Strains used in this study are listed in Supplemental file S5.

### Live Cell Imaging Analysis

Conidiospores were inoculated in 35 mm glass-bottom microwell dishes (MatTech) containing minimal media with glucose as the carbon source and urea as the nitrogen source. Imaging was carried out at room temperature using an Ultraview ERS spinning disk confocal system (Perkin-Elmer) fitted with an Orca-AG camera (Hamamatsu) on a TE2000-U inverted microscope (Nikon) with a 60× 1.40 NA Plan Apochromatic objective (Nikon). For some experiments, imaging was with an Ultraview Vox spinning disk confocal system (Perkin-Elmer) fitted with dual C9100-13 cameras (Hamamatsu) run by Volocity software (Perkin-Elmer). Data are displayed as maximal intensity profiles. Benomyl (Sigma) was used at a concentration of 2.4 µg/ml which is sufficient to depolymerize all microtubules [131]. Image analysis, quantification, pixel line intensity profiles and kymograph generation was carried out using ImageJ freeware (Rasband, WS, ImageJ, US National Institutes of Health, Bethesda, MD, USA, http://rsb.info.nih.gov/ij/, 1997–2008). To quantify histone H1 fluorescence levels the average pixel intensity less the background was determined for an identical sized area for each cell. Each time series was aligned for mitotic entry and values normalized relative to the last frame in G2 before mitotic entry which was set to 100%. Data points represent the mean +/− standard deviation.

### Mass Spectrometry Analysis

Endogenously DLAP tagged kinases were purified from lysates generated from lyophilized mycelia with the following modifications to methodologies previously described [16]. To obtain biomass in a variety of developmental stages, mycelia were harvested from 50 ml large petri dish cultures inoculated with 1×10^8^ conidiospores and grown until asexual spore production was apparent, typically 28–32 hr. 10 dishes grown in this manner typically generated ∼0.6 mg lyophilized dry weight and ∼80–100 mg of total protein extract. A control DLAP construct expressed from the *pyrG* promoter in place of *pyrG* served as a control. For lowly abundant kinases, as estimated by kinase-DLAP fluorescence levels, the volume of S-protein agarose beads (Novagen) was decreased two to four fold from the standard 150 µl packed bead volume/100 mg total protein. To minimize protein degradation, extracts were incubated with S-protein agarose for 15 min at 4° in the presence of protease inhibitors [16]. After extensive washing, purified proteins were eluted from the S-protein agarose beads using SDS-PAGE sample buffer as described [16]. 5–10% of each purified sample was run on analytical SDS-PAGE gels for silver staining or western blotting with an anti-GFP antibody (Clontech). The remaining sample was run on preparative SDS-PAGE gels until proteins had just entered the separating gel and proteins excised as single gel slice [16] which was submitted for LC-MS/MS analysis at the Ohio State University Campus Chemical Instrument Center Mass Spectrometry and Proteomics Facility.

## Supporting Information

File S1 **Supplemental Figures S1–S4, Supplemental table S1.**
(PDF)

File S2 **Plasmid pCDS65 for C-terminal DLAP tagging.**
(PDF)

File S3 **Plasmid pCDS67 for N-terminal DLAP tagging.**
(PDF)

File S4 **Primer List.**
(PDF)

File S5 **Strain List.**
(PDF)

Video S1 **An-Cdc7 locates to mitotic SPBs.** The cell in Figure 8A transiting mitosis showing An-Cdc7-DLAP location together with Nup49-mCherry. Time is in min.
(AVI)

Video S2 **Cells lacking An-Cdc7 undergo multiple cell cycles.** The movie used to generate Figure 8D showing a germinating Δ*An-Cdc7* spore containing GFP-Tubulin and Histone H1-mCherry. Although DNA replication does not occur due to the lack of An-Cdc7 function, this cell attempts mitosis twice separated by an intervening period of interphase. Time is in min.
(AVI)

Video S3 **CkiA^Hrr25^ locates to SPBs and a cytoplasmic focus during SAC arrest.** The movie used to generate Figure 10B showing CkiA^Hrr25^ -DLAP together with Ndc80-mCherry in a cell transiting the mitotic state without microtubules. Time is in min.
(AVI)

Video S4 **SepH displays a biphasic location to SPBs distal from the cell tip during mitosis and septation.** The movie used to generate Figure 12 showing SepH-DLAP, GCP3-mCherry and DIC. The appearance of septa (S1 and S2) is indicated. Time is in min.
(AVI)

## References

[1] Borkovich KA, Ebbole DJ, editors (2010) Cellular and Molecular Biology of Filamentous Fungi: ASM.

[2] Goldman GH, Osmani SA, editors (2008) The Aspergilli: Genomics, Medical Aspects, Biotechnology, and Research Methods: CRC Press.

[3] Arnaud MB, Chibucos MC, Costanzo MC, Crabtree J, Inglis DO, et al (2009) The Aspergillus Genome Database, a curated comparative genomics resource for gene, protein and sequence information for the Aspergillus research community. Nucleic Acids Research 38: D420–D427. 19773420 10.1093/nar/gkp751 PMC2808984

[4] Galagan JE, Calvo SE, Cuomo C, Ma LJ, Wortman JR, et al (2005) Sequencing of *Aspergillus nidulans* and comparative analysis with *A. fumigatus* and *A. oryzae* . Nature 438: 1105–1115. 16372000 10.1038/nature04341

[5] De Souza CP, Hashmi SB, Osmani AH, Andrews P, Ringelberg CS, et al (2013) Functional analysis of the *Aspergillus nidulans* kinome. PLoS One 8: e58008. 23505451 10.1371/journal.pone.0058008 PMC3591445

[6] Szewczyk E, Nayak T, Oakley CE, Edgerton H, Xiong Y, et al (2006) Fusion PCR and gene targeting in *Aspergillus nidulans* . Nat Protoc 1: 3111–3120. 17406574 10.1038/nprot.2006.405

[7] Nayak T, Szewczyk E, Oakley CE, Osmani A, Ukil L, et al (2006) A versatile and efficient gene targeting system for *Aspergillus nidulans* . Genetics 172: 1557–1566. 16387870 10.1534/genetics.105.052563 PMC1456264

[8] Andersen MR, Vongsangnak W, Panagiotou G, Salazar MP, Lehmann L, et al (2008) A trispecies Aspergillus microarray: comparative transcriptomics of three Aspergillus species. Proc Natl Acad Sci U S A 105: 4387–4392. 18332432 10.1073/pnas.0709964105 PMC2393752

[9] Sibthorp C, Wu H, Cowley G, Wong PW, Palaima P, et al (2013) Transcriptome analysis of the filamentous fungus *Aspergillus nidulans* directed to the global identification of promoters. BMC Genomics 14: 847. 24299161 10.1186/1471-2164-14-847 PMC4046813

[10] Bayram O, Krappmann S, Ni M, Bok JW, Helmstaedt K, et al (2008) VelB/VeA/LaeA complex coordinates light signal with fungal development and secondary metabolism. Science 320: 1504–1506. 18556559 10.1126/science.1155888

[11] Wong KH, Todd RB, Oakley BR, Oakley CE, Hynes MJ, et al (2008) Sumoylation in *Aspergillus nidulans*: sumO inactivation, overexpression and live-cell imaging. Fungal Genet Biol 45: 728–737. 18262811 10.1016/j.fgb.2007.12.009 PMC4220683

[12] Liu HL, De Souza CP, Osmani AH, Osmani SA (2009) The Three Fungal Transmembrane Nuclear Pore Complex Proteins of *Aspergillus nidulans* are Dispensable in the Presence of an Intact An-Nup84–120 Complex. Mol Biol Cell 20: 616–630. 19019988 10.1091/mbc.E08-06-0628 PMC2626566

[13] Osmani AH, Davies J, Liu HL, Nile A, Osmani SA (2006) Systematic deletion and mitotic localization of the nuclear pore complex proteins of *Aspergillus nidulans* . Mol Biol Cell 17: 4946–4961. 16987955 10.1091/mbc.E06-07-0657 PMC1679664

[14] Egan MJ, Tan K, Reck-Peterson SL (2012) Lis1 is an initiation factor for dynein-driven organelle transport. J Cell Biol. 10.1083/jcb.201112101 PMC3384415 22711696

[15] Zhang J, Yao X, Fischer L, Abenza JF, Penalva MA, et al (2011) The p25 subunit of the dynactin complex is required for dynein-early endosome interaction. J Cell Biol 193: 1245–1255. 21708978 10.1083/jcb.201011022 PMC3216330

[16] Liu HL, Osmani AH, Ukil L, Son S, Markossian S, et al (2010) Single step affinity purification for fungal proteomics. Eukaryot Cell 9: 831–833. 20363899 10.1128/EC.00032-10 PMC2863959

[17] Hernandez-Rodriguez Y, Hastings S, Momany M (2012) The septin AspB in *Aspergillus nidulans* forms bars and filaments and plays roles in growth emergence and conidiation. Eukaryot Cell 11: 311–323. 22247265 10.1128/EC.05164-11 PMC3294440

[18] Morozov IY, Jones MG, Spiller DG, Rigden DJ, Dattenböck C, et al (2010) Distinct roles for Caf1, Ccr4, Edc3 and CutA in the co-ordination of transcript deadenylation, decapping and P-body formation in *Aspergillus nidulans* . Mol Microbiol 76: 503–516. 20233300 10.1111/j.1365-2958.2010.07118.x

[19] Arratia-Quijada J, Sanchez O, Scazzocchio C, Aguirre J (2012) FlbD, a Myb Transcription Factor of *Aspergillus nidulans*, is Uniquely Involved in both Asexual and Sexual Differentiation. Eukaryot Cell 11: 1132–1142. 22798393 10.1128/EC.00101-12 PMC3445977

[20] Lara-Rojas F, Sanchez O, Kawasaki L, Aguirre J (2011) *Aspergillus nidulans* transcription factor AtfA interacts with the MAPK SakA to regulate general stress responses, development and spore functions. Mol Microbiol 80: 436–454. 21320182 10.1111/j.1365-2958.2011.07581.x PMC3108070

[21] Shen KF, Osmani SA (2013) Regulation of mitosis by the NIMA kinase involves TINA and its newly discovered partner An-WDR8 at spindle pole bodies. Mol Biol Cell 24: 3842–3856. 24152731 10.1091/mbc.E13-07-0422 PMC3861081

[22] Yang L, Ukil L, Osmani A, Nahm F, Davies J, et al (2004) Rapid production of gene replacement constructs and generation of a green fluorescent protein-tagged centromeric marker in *Aspergillus nidulans* . Eukaryot Cell 3: 1359–1362. 15470263 10.1128/EC.3.5.1359-1362.2004 PMC522605

[23] Clutterbuck AJ (1970) Synchronous nuclear division and septation in *Aspergillus nidulans* . J Gen Micro 60: 133–135. 10.1099/00221287-60-1-133 5488461

[24] Harris SD (2001) Septum formation in *Aspergillus nidulans* Curr Opin Microbiol. 4: 736–739. 10.1016/s1369-5274(01)00276-4 11731327

[25] Bruno KS, Morrell JL, Hamer JE, Staiger CJ (2001) SEPH, a Cdc7p orthologue from *Aspergillus nidulans*, functions upstream of actin ring formation during cytokinesis. Mol Microbiol 42: 3–12. 11679062 10.1046/j.1365-2958.2001.02605.x

[26] Kim JM, Lu L, Shao R, Chin J, Liu B (2006) Isolation of mutations that bypass the requirement of the septation initiation network for septum formation and conidiation in *Aspergillus nidulans* . Genetics 173: 685–696. 16624915 10.1534/genetics.105.054304 PMC1526526

[27] Harris SD, Morrell JL, Hamer JE (1994) Identification and characterization of *Aspergillus nidulans* mutants defective in cytokinesis. Genetics 136: 517–532. 8150280 10.1093/genetics/136.2.517 PMC1205805

[28] Kim JM, Zeng CJ, Nayak T, Shao R, Huang AC, et al (2009) Timely septation requires SNAD-dependent spindle pole body localization of the septation initiation network components in the filamentous fungus *Aspergillus nidulans* . Mol Biol Cell 20: 2874–2884. 19386763 10.1091/mbc.E08-12-1177 PMC2695795

[29] Morris NR (1976) Mitotic mutants of *Aspergillus nidulans* . Genet Res Camb 26: 237–254. 10.1017/s0016672300016049 773766

[30] Westfall PJ, Momany M (2002) *Aspergillus nidulans* septin AspB plays pre- and postmitotic roles in septum, branch, and conidiophore development. Mol Biol Cell 13: 110–118. 11809826 10.1091/mbc.01-06-0312 PMC65076

[31] Takeshita N, Vienken K, Rolbetzki A, Fischer R (2007) The *Aspergillus nidulans* putative kinase, KfsA (kinase for septation), plays a role in septation and is required for efficient asexual spore formation. Fungal Genet Biol 44: 1205–1214. 17500016 10.1016/j.fgb.2007.03.006

[32] Si H, Justa-Schuch D, Seiler S, Harris SD (2010) Regulation of Septum Formation by the Bud3-Rho4 GTPase Module in *Aspergillus nidulans* . Genetics 185: 165–176. 20176976 10.1534/genetics.110.114165 PMC2870952

[33] Manning G, Whyte DB, Martinez R, Hunter T, Sudarsanam S (2002) The protein kinase complement of the human genome. Science 298: 1912–1934. 12471243 10.1126/science.1075762

[34] Bettencourt-Dias M, Giet R, Sinka R, Mazumdar A, Lock WG, et al (2004) Genome-wide survey of protein kinases required for cell cycle progression. Nature 432: 980–987. 15616552 10.1038/nature03160

[35] Wang C, Zhang S, Hou R, Zhao Z, Zheng Q, et al (2011) Functional analysis of the kinome of the wheat scab fungus *Fusarium graminearum* . PLoS Pathogens 7: e1002460. 22216007 10.1371/journal.ppat.1002460 PMC3245316

[36] Judelson HS, Ah-Fong AMV (2010) The kinome of *Phytophthora infestans* reveals oomycete-specific innovations and links to other taxonomic groups. BMC Genomics 11: 700. 21143935 10.1186/1471-2164-11-700 PMC3019232

[37] Park G, Servin JA, Turner GE, Altamirano L, Colot HV, et al (2011) Global analysis of serine-threonine protein kinase genes in *Neurospora crassa* . Eukaryot Cell 10: 1553–1564. 21965514 10.1128/EC.05140-11 PMC3209061

[38] Breitkreutz A, Choi H, Sharom JR, Boucher L, Neduva V, et al (2010) A global protein kinase and phosphatase interaction network in yeast. Science 328: 1043–1046. 20489023 10.1126/science.1176495 PMC3983991

[39] Plowman GD, Sudarsanam S, Bingham J, Whyte D, Hunter T (1999) The protein kinases of *Caenorhabditis elegans*: a model for signal transduction in multicellular organisms. Proc Natl Acad Sci U S A 96: 13603–13610. 10570119 10.1073/pnas.96.24.13603 PMC24111

[40] Carmena M, Wheelock M, Funabiki H, Earnshaw WC (2012) The chromosomal passenger complex (CPC): from easy rider to the godfather of mitosis. Nat Rev Mol Cell Biol 13: 789–803. 23175282 10.1038/nrm3474 PMC3729939

[41] Archambault V, Glover DM (2009) Polo-like kinases: conservation and divergence in their functions and regulation. Nat Rev Mol Cell Biol 10: 265–275. 19305416 10.1038/nrm2653

[42] Liu X, Winey M (2012) The MPS1 Family of Protein Kinases. Annu Rev Biochem 81: 561–585. 22482908 10.1146/annurev-biochem-061611-090435 PMC4026297

[43] Stracker TH, Usui T, Petrini JH (2009) Taking the time to make important decisions: the checkpoint effector kinases Chk1 and Chk2 and the DNA damage response. DNA Repair 8: 1047–1054. 19473886 10.1016/j.dnarep.2009.04.012 PMC2725228

[44] Taylor SS, Zhang P, Steichen JM, Keshwani MM, Kornev AP (2013) PKA: lessons learned after twenty years. Biochim Biophys Acta 1834: 1271–1278. 23535202 10.1016/j.bbapap.2013.03.007 PMC3763834

[45] Osmani SA, Pu RT, Morris NR (1988) Mitotic induction and maintenance by overexpression of a G2- specific gene that encodes a potential protein kinase. Cell 53: 237–244. 3359487 10.1016/0092-8674(88)90385-6

[46] Osmani SA, May GS, Morris NR (1987) Regulation of the mRNA levels of *nimA*, a gene required for the G2-M transition in *Aspergillus nidulans* . J Cell Biol 104: 1495–1504. 3294854 10.1083/jcb.104.6.1495 PMC2114495

[47] Veneault-Fourrey C, Barooah M, Egan M, Wakley G, Talbot NJ (2006) Autophagic fungal cell death is necessary for infection by the rice blast fungus. Science 312: 580–583. 16645096 10.1126/science.1124550

[48] Fry AM, O’Regan L, Sabir SR, Bayliss R (2012) Cell cycle regulation by the NEK family of protein kinases. J Cell Sci 125: 4423–4433. 23132929 10.1242/jcs.111195 PMC3500863

[49] Laurell E, Beck K, Krupina K, Theerthagiri G, Bodenmiller B, et al (2011) Phosphorylation of Nup98 by Multiple Kinases Is Crucial for NPC Disassembly during Mitotic Entry. Cell 144: 539–550. 21335236 10.1016/j.cell.2011.01.012

[50] Agromayor M, Martin-Serrano J (2013) Knowing when to cut and run: mechanisms that control cytokinetic abscission. Trends Cell Biol 23: 433–441. 23706391 10.1016/j.tcb.2013.04.006

[51] van der Waal MS, Hengeveld RCC, van der Horst A, Lens SMA (2012) Cell division control by the Chromosomal Passenger Complex. Exp Cell Res 318: 1407–1420. 22472345 10.1016/j.yexcr.2012.03.015

[52] Fisk HA, Mattison CP, Winey M (2003) Human Mps1 protein kinase is required for centrosome duplication and normal mitotic progression. Proc Natl Acad Sci U S A 100: 14875–14880. 14657364 10.1073/pnas.2434156100 PMC299837

[53] Lara-Gonzalez P, Westhorpe Frederick G, Taylor Stephen S (2012) The Spindle Assembly Checkpoint. Curr Biol 22: R966–R980. 23174302 10.1016/j.cub.2012.10.006

[54] Musacchio A, Salmon ED (2007) The spindle-assembly checkpoint in space and time. Nat Rev Mol Cell Biol 8: 379–393. 17426725 10.1038/nrm2163

[55] Montembault E, Dutertre S, Prigent C, Giet R (2007) PRP4 is a spindle assembly checkpoint protein required for MPS1, MAD1, and MAD2 localization to the kinetochores. J Cell Biol 179: 601–609. 17998396 10.1083/jcb.200703133 PMC2080909

[56] Ukil L, Varadaraj A, Govindaraghavan M, Liu HL, Osmani SA (2008) Copy number suppressors of the *Aspergillus nidulans nimA*1 mitotic kinase display distinctive and highly dynamic cell cycle-regulated locations. Eukaryot Cell 7: 2087–2099. 18931041 10.1128/EC.00278-08 PMC2593184

[57] Kim JS, Raines RT (1993) Ribonuclease S-peptide as a carrier in fusion proteins. Protein Sci 2: 348–356. 8453373 10.1002/pro.5560020307 PMC2142386

[58] Richards FM (1955) Titration of amino groups released during the digestion of ribonuclease by subtilisin. C R Trav Lab Carlsberg Chim 29: 322–328. 13353909

[59] De Souza CP, Hashmi SB, Yang X, Osmani SA (2011) Regulated inactivation of the spindle assembly checkpoint without functional mitotic spindles. EMBO J 30: 2648–2661. 21642954 10.1038/emboj.2011.176 PMC3155307

[60] Shi J, Chen W, Liu Q, Chen S, Hu H, et al (2008) Depletion of the MobB and CotA complex in *Aspergillus nidulans* causes defects in polarity maintenance that can be suppressed by the environment stress. Fungal Genet Biol 45: 1570–1581. 18832040 10.1016/j.fgb.2008.08.011

[61] De Souza CP, Ye X, Osmani SA (1999) Checkpoint defects leading to premature mitosis also cause endoreplication of DNA in *Aspergillus nidulans* . Mol Biol Cell 10: 3661–3674. 10564263 10.1091/mbc.10.11.3661 PMC25657

[62] Osmani AH, van Peij N, Mischke M, O’Connell MJ, Osmani SA (1994) A single p34^cdc2^ protein kinase (nimX^cdc2^) is required at G1 and G2 in *Aspergillus nidulans* . J Cell Sci 107: 1519–1528. 7962194 10.1242/jcs.107.6.1519

[63] Johns SA, Leeder AC, Safaie M, Turner G (2006) Depletion of *Aspergillus nidulans cotA* causes a severe polarity defect which is not suppressed by the nuclear migration mutation *nudA*2. Mol Genet Genomics 275: 593–604. 16506053 10.1007/s00438-006-0113-0

[64] Kornstein LB, Gaiso ML, Hammell RL, Bartelt DC (1992) Cloning and sequence determination of a cDNA encoding *Aspergillus nidulans* calmodulin-dependent multifunctional protein kinase. Gene 113: 75–82. 1563634 10.1016/0378-1119(92)90671-b

[65] Anaya P, Evans SC, Dai C, Lozano G, May GS (1998) Isolation of the *Aspergillus nidulans sudD* gene and its human homologue. Gene 211: 323–329. 9602165 10.1016/s0378-1119(98)00115-2

[66] Efimov VP, Morris NR (1998) A screen for dynein synthetic lethals in *Aspergillus nidulans* identifies spindle assembly checkpoint genes and other genes involved in mitosis. Genetics 149: 101–116. 9584089 10.1093/genetics/149.1.101 PMC1460152

[67] Hofmann AF, Harris SD (2000) The *Aspergillus nidulans uvsB* gene encodes an ATM-related kinase required for multiple facets of the DNA damage response. Genetics 154: 1577–1586. 10747054 10.1093/genetics/154.4.1577 PMC1461047

[68] Huh WK, Falvo JV, Gerke LC, Carroll AS, Howson RW, et al (2003) Global analysis of protein localization in budding yeast. Nature 425: 686–691. 14562095 10.1038/nature02026

[69] Tibbetts RS (2000) Functional interactions between BRCA1 and the checkpoint kinase ATR during genotoxic stress. Genes Dev 14: 2989–3002. 11114888 10.1101/gad.851000 PMC317107

[70] Tkach JM, Yimit A, Lee AY, Riffle M, Costanzo M, et al (2012) Dissecting DNA damage response pathways by analysing protein localization and abundance changes during DNA replication stress. Nat Cell Biol 14: 966–976. 22842922 10.1038/ncb2549 PMC3434236

[71] De Souza CP, Osmani AH, Hashmi SB, Osmani SA (2004) Partial nuclear pore complex disassembly during closed mitosis in *Aspergillus nidulans* . Curr Biol 14: 1973–1984. 15556859 10.1016/j.cub.2004.10.050

[72] Ovechkina Y, Maddox P, Oakley CE, Xiang X, Osmani SA, et al (2003) Spindle formation in Aspergillus is coupled to tubulin movement into the nucleus. Mol Biol Cell 14: 2192–2200. 12802085 10.1091/mbc.E02-10-0641 PMC165107

[73] Matsuyama A, Arai R, Yashiroda Y, Shirai A, Kamata A, et al (2006) ORFeome cloning and global analysis of protein localization in the fission yeast *Schizosaccharomyces pombe* . Nat Biotechnol 24: 841–847. 16823372 10.1038/nbt1222

[74] Vanrobays E, Gleizes PE, Bousquet-Antonelli C, Noaillac-Depeyre J, Caizergues-Ferrer M, et al (2001) Processing of 20S pre-rRNA to 18S ribosomal RNA in yeast requires Rrp10p, an essential non-ribosomal cytoplasmic protein. EMBO J 20: 4204–4213. 11483523 10.1093/emboj/20.15.4204 PMC149176

[75] Madrid M, Soto T, Khong HK, Franco A, Vicente J, et al (2006) Stress-induced response, localization, and regulation of the Pmk1 cell integrity pathway in *Schizosaccharomyces pombe* . J Biol Chem 281: 2033–2043. 16291757 10.1074/jbc.M506467200

[76] Kimata Y, Kohno K (2011) Endoplasmic reticulum stress-sensing mechanisms in yeast and mammalian cells. Curr Opin Cell Biol 23: 135–142. 21093243 10.1016/j.ceb.2010.10.008

[77] Markina-Inarrairaegui A, Pantazopoulou A, Espeso EA, Penalva MA (2013) The *Aspergillus nidulans* peripheral ER: disorganization by ER stress and persistence during mitosis. PLoS One 8: e67154. 23826221 10.1371/journal.pone.0067154 PMC3691152

[78] Abenza JF, Galindo A, Pinar M, Pantazopoulou A, de los Rios V, et al (2012) Endosomal maturation by Rab conversion in *Aspergillus nidulans* is coupled to dynein-mediated basipetal movement. Mol Biol Cell 23: 1889–1901. 22456509 10.1091/mbc.E11-11-0925 PMC3350553

[79] Penalva MA, Galindo A, Abenza JF, Pinar M, Calcagno-Pizarelli AM, et al (2012) Searching for gold beyond mitosis: Mining intracellular membrane traffic in *Aspergillus nidulans* . Cell Logist 2: 2–14. 22645705 10.4161/cl.19304 PMC3355971

[80] Abenza JF, Pantazopoulou A, Rodríguez JM, Galindo A, Peñalva MA (2009) Long-Distance Movement of *Aspergillus nidulans* Early Endosomes on Microtubule Tracks. Traffic 10: 57–75. 19000168 10.1111/j.1600-0854.2008.00848.x

[81] Sturgill TW, Cohen A, Diefenbacher M, Trautwein M, Martin DE, et al (2008) TOR1 and TOR2 have distinct locations in live cells. Eukaryot Cell 7: 1819–1830. 18723607 10.1128/EC.00088-08 PMC2568074

[82] Zoncu R, Efeyan A, Sabatini DM (2011) mTOR: from growth signal integration to cancer, diabetes and ageing. Nat Rev Mol Cell Biol 12: 21–35. 21157483 10.1038/nrm3025 PMC3390257

[83] Sancak Y, Bar-Peled L, Zoncu R, Markhard AL, Nada S, et al (2010) Ragulator-Rag complex targets mTORC1 to the lysosomal surface and is necessary for its activation by amino acids. Cell 141: 290–303. 20381137 10.1016/j.cell.2010.02.024 PMC3024592

[84] Flinn RJ, Yan Y, Goswami S, Parker PJ, Backer JM (2010) The Late Endosome is Essential for mTORC1 Signaling. Mol Biol Cell 21: 833–841. 20053679 10.1091/mbc.E09-09-0756 PMC2828969

[85] Valbuena N, Guan KL, Moreno S (2012) The Vam6 and Gtr1-Gtr2 pathway activates TORC1 in response to amino acids in fission yeast. J Cell Sci 125: 1920–1928. 22344254 10.1242/jcs.094219 PMC3656621

[86] Betz C, Hall MN (2013) Where is mTOR and what is it doing there? J Cell Biol 203: 563–574. 24385483 10.1083/jcb.201306041 PMC3840941

[87] Pei Y, Du H, Singer J, St. Amour C, Granitto S, et al (2006) Cyclin-Dependent Kinase 9 (Cdk9) of Fission Yeast Is Activated by the CDK-Activating Kinase Csk1, Overlaps Functionally with the TFIIH-Associated Kinase Mcs6, and Associates with the mRNA Cap Methyltransferase Pcm1 In Vivo. Mol Cell Biol 26: 777–788. 16428435 10.1128/MCB.26.3.777-788.2006 PMC1347026

[88] Wakabayashi M, Ishii C, Hatakeyama S, Inoue H, Tanaka S (2010) ATM and ATR homologes of *Neurospora crassa* are essential for normal cell growth and maintenance of chromosome integrity. Fungal Genet Biol 47: 809–817. 20553930 10.1016/j.fgb.2010.05.010

[89] Suijkerbuijk Saskia JE, van Dam Teunis JP, Karagöz GE, von Castelmur E, Hubner Nina C, et al (2012) The Vertebrate Mitotic Checkpoint Protein BUBR1 Is an Unusual Pseudokinase. Dev Cell 22: 1321–1329. 22698286 10.1016/j.devcel.2012.03.009

[90] De Souza CP, Hashmi SB, Nayak T, Oakley B, Osmani SA (2009) Mlp1 acts as a mitotic scaffold to spatially regulate spindle assembly checkpoint proteins in *Aspergillus nidulans* . Mol Biol Cell 20: 2146–2159. 19225157 10.1091/mbc.E08-08-0878 PMC2669023

[91] Wang X, Babu JR, Harden JM, Jablonski SA, Gazi MH, et al (2001) The Mitotic Checkpoint Protein hBUB3 and the mRNA Export Factor hRAE1 Interact with GLE2p-binding Sequence (GLEBS)-containing Proteins. J Biol Chem 276: 26559–26567. 11352911 10.1074/jbc.M101083200

[92] Nayak T, Edgerton-Morgan H, Horio T, Xiong Y, De Souza CP, et al (2010) Gamma-tubulin regulates the anaphase-promoting complex/cyclosome during interphase. J Cell Biol 190: 317–330. 20679430 10.1083/jcb.201002105 PMC2922653

[93] Wu L, Osmani SA, Mirabito PM (1998) A role for NIMA in the nuclear localization of cyclin B in *Aspergillus nidulans* . J Cell Biol 141: 1575–1587. 9647650 10.1083/jcb.141.7.1575 PMC2133011

[94] Ye XS, Xu G, Pu PT, Fincher RR, McGuire SL, et al (1995) The NIMA protein kinase is hyperphosphorylated and activated downstream of p34^cdc2^/cyclin B: coordination of two mitosis promoting kinases. EMBO J 14: 986–994. 7889944 10.1002/j.1460-2075.1995.tb07079.x PMC398170

[95] Martin-Castellanos C, Blanco MA, de Prada JM, Moreno S (2000) The puc1 cyclin regulates the G1 phase of the fission yeast cell cycle in response to cell size. Mol Biol Cell 11: 543–554. 10679013 10.1091/mbc.11.2.543 PMC14792

[96] James SW, Bullock KA, Gygax SE, Kraynack BA, Matura RA, et al (1999) *nimO*, an Aspergillus gene related to budding yeast *dbf4*, is required for DNA synthesis and mitotic checkpoint control. J Cell Sci 112: 1313–1324. 10194410 10.1242/jcs.112.9.1313

[97] Masai H, Arai K (2002) Cdc7 kinase complex: a key regulator in the initiation of DNA replication. J Cell Physiol 190: 287–296. 11857444 10.1002/jcp.10070

[98] Xiong Y, Oakley BR (2009) *In vivo* analysis of the functions of gamma-tubulin complex proteins. J Cell Sci. 10.1242/jcs.059196 PMC2776505 19861490

[99] Natsume T, Muller CA, Katou Y, Retkute R, Gierlinski M, et al (2013) Kinetochores coordinate pericentromeric cohesion and early DNA replication by Cdc7-Dbf4 kinase recruitment. Mol Cell 50: 661–674. 23746350 10.1016/j.molcel.2013.05.011 PMC3679449

[100] Weinreich M, Stillman B (1999) Cdc7p-Dbf4p kinase binds to chromatin during S phase and is regulated by both the APC and the RAD53 checkpoint pathway. EMBO J 18: 5334–5346. 10508166 10.1093/emboj/18.19.5334 PMC1171603

[101] Ferreira MF, Santocanale C, Drury LS, Diffley JF (2000) Dbf4p, an essential S phase-promoting factor, is targeted for degradation by the anaphase-promoting complex. Mol Cell Biol 20: 242–248. 10594027 10.1128/mcb.20.1.242-248.2000 PMC85080

[102] Knockleby J, Lee H (2010) Same partners, different dance: Involvement of DNA replication proteins in centrosome regulation. Cell Cycle 9: 4487–4491. 21088489 10.4161/cc.9.22.14047

[103] Hossain M, Stillman B (2012) Meier-Gorlin syndrome mutations disrupt an Orc1 CDK inhibitory domain and cause centrosome reduplication. Genes Dev 26: 1797–1810. 22855792 10.1101/gad.197178.112 PMC3426759

[104] Hemerly AS, Prasanth SG, Siddiqui K, Stillman B (2009) Orc1 controls centriole and centrosome copy number in human cells. Science 323: 789–793. 19197067 10.1126/science.1166745 PMC2653626

[105] Ye XS, Fincher RR, Tang A, McNeal KK, Gygax SE, et al (1997) Proteolysis and tyrosine phosphorylation of p34^cdc2^/Cyclin B; the role of MCM2 and initiation of DNA replication to allow tyrosine phosphorylation of p34^cdc2^ . J Biol Chem 272: 33384–33393. 9407133 10.1074/jbc.272.52.33384

[106] Takahashi TS, Basu A, Bermudez V, Hurwitz J, Walter JC (2008) Cdc7-Drf1 kinase links chromosome cohesion to the initiation of DNA replication in Xenopus egg extracts. Genes Dev 22: 1894–1905. 18628396 10.1101/gad.1683308 PMC2492736

[107] Bailis JM, Bernard P, Antonelli R, Allshire RC, Forsburg SL (2003) Hsk1–Dfp1 is required for heterochromatin-mediated cohesion at centromeres. Nat Cell Biol 5: 1111–1116. 14625560 10.1038/ncb1069

[108] Reyes-Dominguez Y, Bok JW, Berger H, Shwab EK, Basheer A, et al (2010) Heterochromatic marks are associated with the repression of secondary metabolism clusters in *Aspergillus nidulans* . Mol Microbiol 76: 1376–1386. 20132440 10.1111/j.1365-2958.2010.07051.x PMC2904488

[109] Bachewich C, Masker K, Osmani S (2005) The polo-like kinase PLKA is required for initiation and progression through mitosis in the filamentous fungus *Aspergillus nidulans* . Mol Microbiol 55: 572–587. 15659171 10.1111/j.1365-2958.2004.04404.x

[110] Miller CT, Gabrielse C, Chen Y-C, Weinreich M (2009) Cdc7p-Dbf4p Regulates Mitotic Exit by Inhibiting Polo Kinase. PLoS Genetics 5: e1000498. 19478884 10.1371/journal.pgen.1000498 PMC2682205

[111] Apostolaki A, Harispe L, Calcagno-Pizarelli AM, Vangelatos I, Sophianopoulou V, et al (2012) *Aspergillus nidulans* CkiA is an essential casein kinase I required for delivery of amino acid transporters to the plasma membrane. Mol Micro 84: 530–549. 10.1111/j.1365-2958.2012.08042.x PMC3491690 22489878

[112] Lusk CP, Waller DD, Makhnevych T, Dienemann A, Whiteway M, et al (2007) Nup53p is a target of two mitotic kinases, Cdk1p and Hrr25p. Traffic 8: 647–660. 17461799 10.1111/j.1600-0854.2007.00559.x

[113] Petronczki M, Matos J, Mori S, Gregan J, Bogdanova A, et al (2006) Monopolar attachment of sister kinetochores at meiosis I requires casein kinase 1. Cell 126: 1049–1064. 16990132 10.1016/j.cell.2006.07.029

[114] Johnson AE, Chen JS, Gould KL (2013) CK1 Is Required for a Mitotic Checkpoint that Delays Cytokinesis. Curr Biol 23: 1920–1926. 24055157 10.1016/j.cub.2013.07.077 PMC4078987

[115] Konzack S, Rischitor PE, Enke C, Fischer R (2005) The role of the kinesin motor KipA in microtubule organization and polarized growth of *Aspergillus nidulans* . Mol Biol Cell 16: 497–506. 15563609 10.1091/mbc.E04-02-0083 PMC545884

[116] Greer YE, Rubin JS (2011) The role of centrosomal casein kinase 1 delta in neurite outgrowth and beyond. Cell Cycle 10: 2605–2606. 21785261 10.4161/cc.10.16.16386

[117] Zyss D, Ebrahimi H, Gergely F (2011) Casein kinase I delta controls centrosome positioning during T cell activation. J Cell Biol 195: 781–797. 22123863 10.1083/jcb.201106025 PMC3257584

[118] Kafadar KA (2003) Negative regulation of calcineurin signaling by Hrr25p, a yeast homolog of casein kinase I. Genes Dev. 17: 2698–2708. 10.1101/gad.1140603 PMC280619 14597664

[119] Matos J, Lipp JJ, Bogdanova A, Guillot S, Okaz E, et al (2008) Dbf4-dependent CDC7 kinase links DNA replication to the segregation of homologous chromosomes in meiosis I. Cell. 135: 662–678. 10.1016/j.cell.2008.10.026 19013276

[120] Taheri-Talesh N, Xiong Y, Oakley BR (2012) The Functions of Myosin II and Myosin V Homologs in Tip Growth and Septation in *Aspergillus nidulans* . PLoS One 7: e31218. 22359575 10.1371/journal.pone.0031218 PMC3281053

[121] Garcia-Cortes JC, McCollum D (2009) Proper timing of cytokinesis is regulated by *Schizosaccharomyces pombe* Etd1. J Cell Biol 186: 739–753. 19736319 10.1083/jcb.200902116 PMC2742193

[122] Sohrmann M, Schmidt S, Hagan I, Simanis V (1998) Asymmetric segregation on spindle poles of the *Schizosaccharomyces pombe* septum-inducing protein kinase Cdc7p. Genes Dev 12: 84–94. 9420333 10.1101/gad.12.1.84 PMC316397

[123] Goyal A, Takaine M, Simanis V, Nakano K (2011) Dividing the spoils of growth and the cell cycle: The fission yeast as a model for the study of cytokinesis. Cytoskeleton 68: 69–88. 21246752 10.1002/cm.20500 PMC3044818

[124] Feoktistova A, Morrell-Falvey J, Chen JS, Singh NS, Balasubramanian MK, et al (2012) The fission yeast SIN kinase, Sid2, is required for SIN asymmetry and regulates the SIN scaffold, Cdc11. Mol Biol Cell. 10.1091/mbc.E11-09-0792 PMC3338431 22419817

[125] Grallert A, Krapp A, Bagley S, Simanis V, Hagan IM (2004) Recruitment of NIMA kinase shows that maturation of the *S. pombe* spindle-pole body occurs over consecutive cell cycles and reveals a role for NIMA in modulating SIN activity. Genes Dev 18: 1007–1021. 15132994 10.1101/gad.296204 PMC406291

[126] Suelmann R, Sievers N, Fischer R (1997) Nuclear traffic in fungal hyphae: in vivo study of nuclear migration and positioning in *Aspergillus nidulans* . Mol Microbiol 25: 757–769. 9379904 10.1046/j.1365-2958.1997.5131873.x

[127] Seiler S, Justa-Schuch D (2010) Conserved components, but distinct mechanisms for the placement and assembly of the cell division machinery in unicellular and filamentous ascomycetes. Mol Microbiol 78: 1058–1076. 21091496 10.1111/j.1365-2958.2010.07392.x

[128] Johnson AE, McCollum D, Gould KL (2012) Polar opposites: Fine-tuning cytokinesis through SIN asymmetry. Cytoskeleton 69: 686–699. 22786806 10.1002/cm.21044 PMC3478943

[129] Guertin DA, Chang L, Irshad F, Gould KL, McCollum D (2000) The role of the sid1p kinase and cdc14p in regulating the onset of cytokinesis in fission yeast. EMBO J 19: 1803–1815. 10775265 10.1093/emboj/19.8.1803 PMC302011

[130] Grallert A, Connolly Y, Smith DL, Simanis V, Hagan IM (2012) The *S. pombe* cytokinesis NDR kinase Sid2 activates Fin1 NIMA kinase to control mitotic commitment through Pom1/Wee1. Nat Cell Biol 10: 738–745. 10.1038/ncb2514 PMC4284365 22684255

[131] Horio T, Oakley BR (2005) The role of microtubules in rapid hyphal tip growth of *Aspergillus nidulans* . Mol Biol Cell 16: 918–926. 15548594 10.1091/mbc.E04-09-0798 PMC545922

