# Supplementary material for: Application of a New Dual Localization-Affinity Purification Tag Reveals Novel Aspects of Protein Kinase Biology in Aspergillus nidulans
Source: PLoS One. 2014 Mar 5;9(3):e90911. doi: 10.1371/journal.pone.0090911 (PMC3944740; doi:10.1371/journal.pone.0090911)
Supplement: File S1 — Supplemental Figures S1–S4, Supplemental table S1. (PDF) [file pone.0090911.s001.pdf]

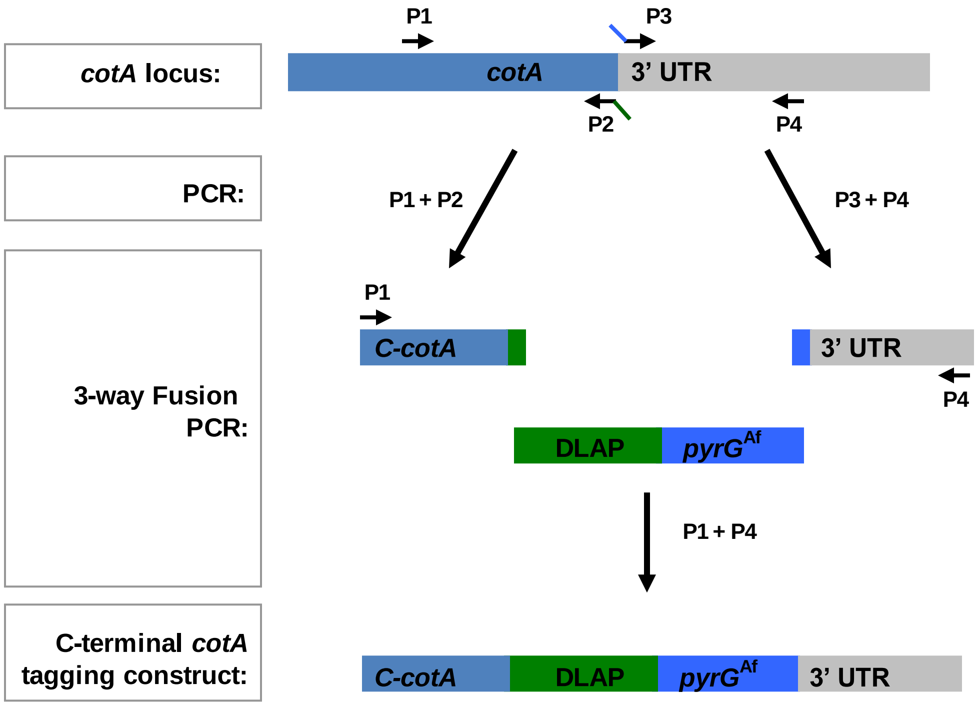

Figure S1. Schematic diagram depicting the generation of a CotA-DLAP construct for endogenous gene replacement. 5' and 3' gene specific fragments designed to insert the DLAP::*pyrG*^{Af} cassette in frame with *cotA* are first PCR amplified from *A. nidulans* genomic DNA. Primers P2 and P3 have 5' extensions complementary to the DLAP::*pyrG*^{Af} cassette amplified from pCDS65. The full length gene replacement construct is then generated by 3-way fusion PCR for transformation.

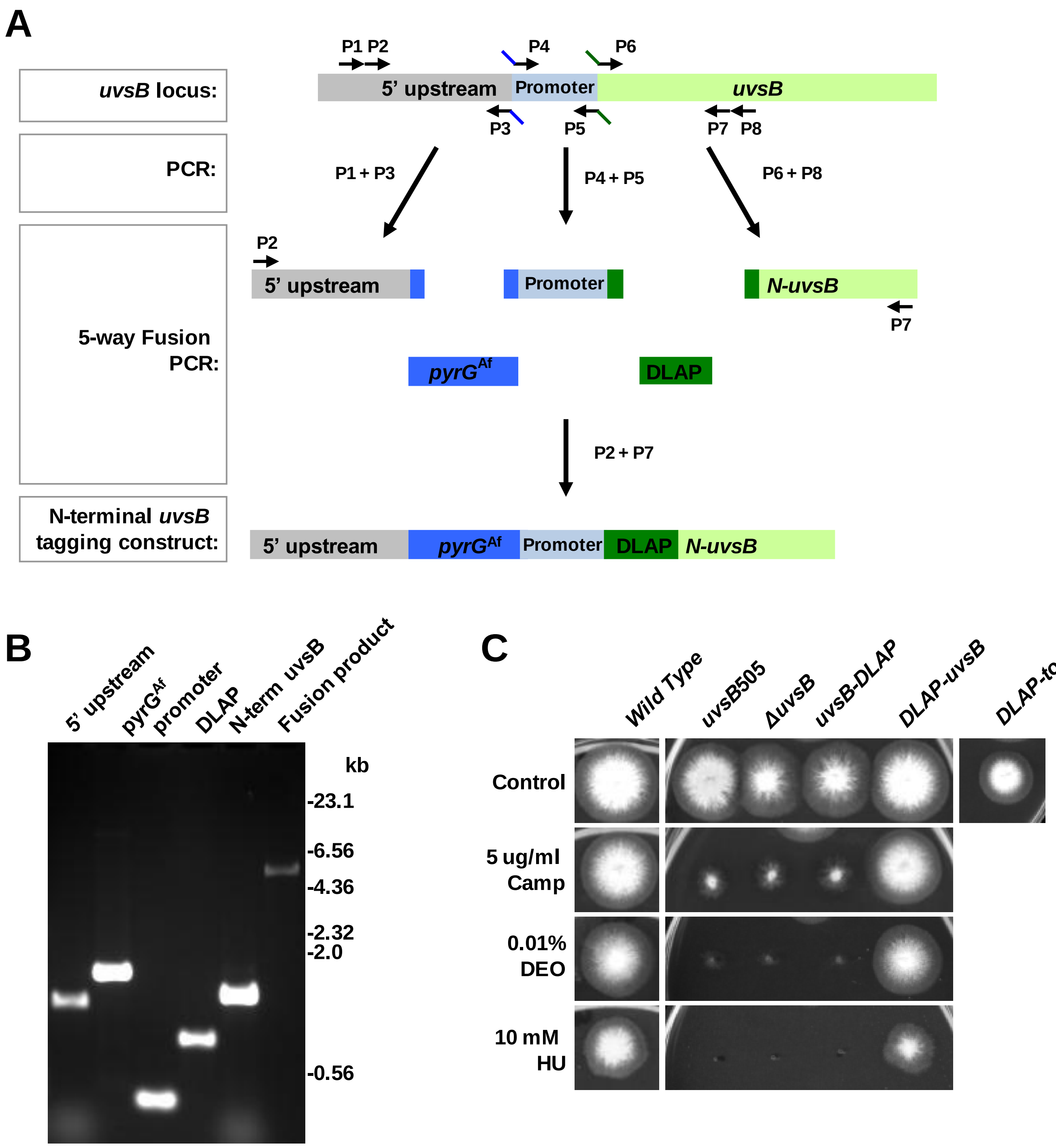

Figure S2. N-terminally DLAP tagged versions of UvsB^{ATR} and TorA are functional. **A** Schematic depicting generation of the DLAP-*uvsB*^{ATR} construct for endogenous gene replacement. DLAP is landed in frame with *uvsB*^{ATR} and expressed using the endogenous *uvsB*^{ATR} promoter. 5' and 3' gene specific fragments and the promoter region are first PCR amplified from *A. nidulans* genomic DNA. Primers P3 and P4 have 5' extensions complementary to the *pyrG*^{Af} cassette while P5 and P6 have 5' extensions complementary to the N-terminal DLAP cassette amplified from pCDS67. The full length gene replacement construct is then generated by 5-way fusion PCR for transformation. **B** Gel showing the 5 individual PCR products and the final 5-way fusion PCR product. **C** Colony growth of the indicated strains. DLAP-UvsB^{ATR} is functional as it does not cause the genotoxic stress sensitivities displayed by the Δ*uvsB*^{ATR} and UvsB^{ATR}-DLAP strains. DLAP-TorA is functional as it does not cause the lethal phenotype of the null allele.

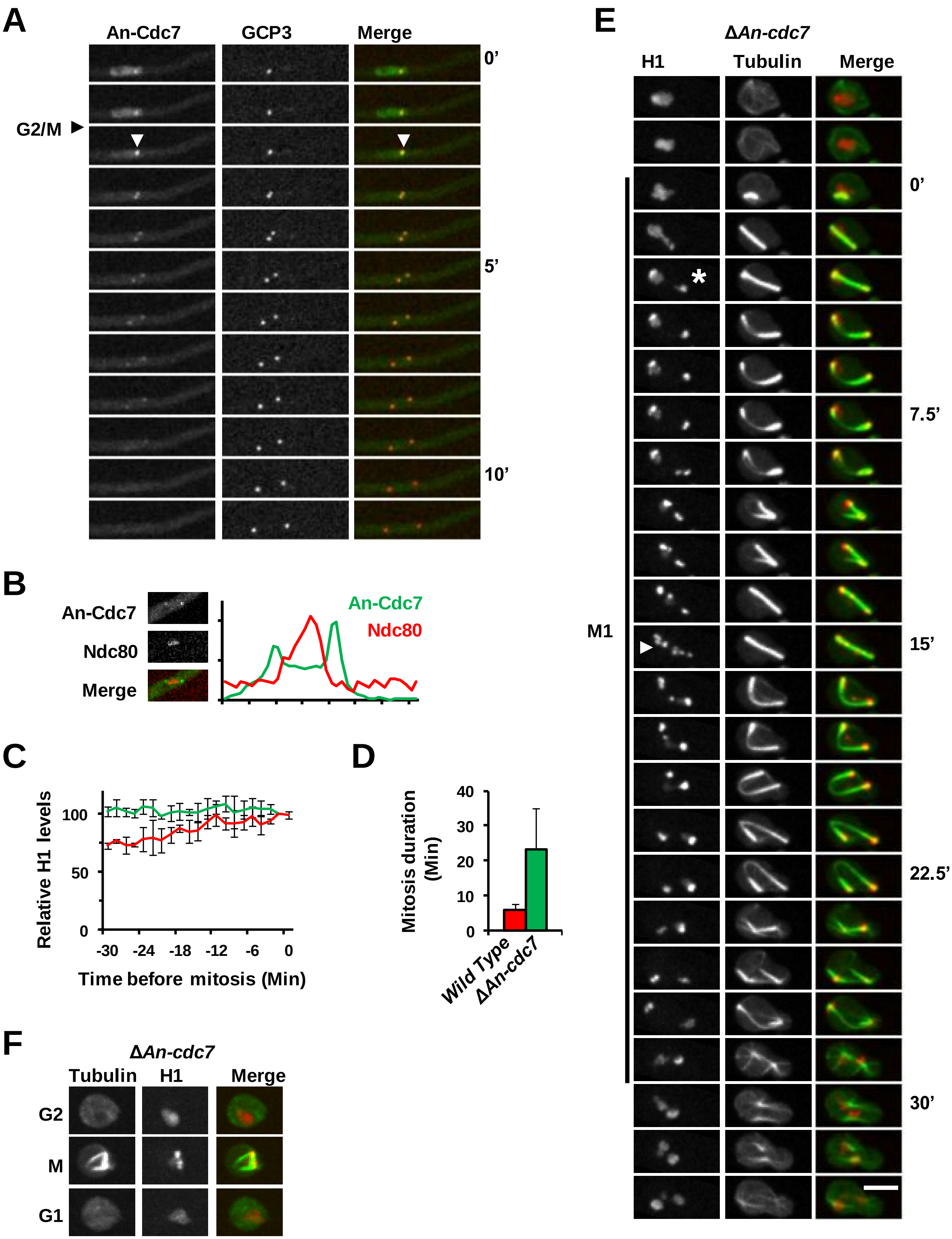

Figure S3. Cells lacking the An-Cdc7 kinase cycle through multiple cell cycles without DNA replication or successful mitosis. **A** Time lapse images of a cell transiting mitosis showing that An-Cdc7-DLAP locates to SPBs indicated by GCP3-mCherry. **B** Images and pixel line intensity profile showing that An-Cdc7 locates to

the spindle poles and is not concentrated on kinetochores visualized by Ndc80-mCherry during metaphase. **C** Graph showing relative levels of histone H1 fluorescence in wild type and *ΔAn-cdc7* cells during the 30 min preceding mitotic entry (n=4, error bars indicate standard deviation). Wild type and *ΔAn-cdc7* cells were imaged together following germination from a heterokaryon at 32°. **D** Graph showing the duration of mitosis for wild type and *ΔAn-cdc7* cells (n=8, error bars indicate standard deviation). **E** Time lapse images showing all time points during the first mitotic arrest shown in Figure 8D and Video S2. Note that early in the mitotic arrest unequal amounts of DNA are present at the spindle poles (asterisk) but then the DNA moves back along the spindle and up to 8 distinct histone H1 foci are apparent along the spindle (15 min arrowhead). **F** A *ΔAn-cdc7* cell which transits a mitotic arrest in which it attempts to segregate its unreplicated DNA before undergoing SIME and forming a single interphase nucleus. Bar ~ 10 µm.

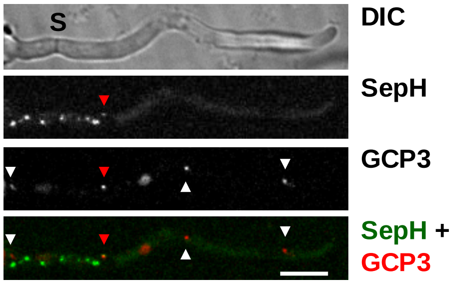

Figure S4. SepH locates to non-SPB foci and a subset of SPBs. A germling with a single septum (S) and with the apical cell in G2 showing SepH-DLAP together with the GCP3-mCherry SPB marker. The majority of SepH foci are distinct from SPBs (arrowheads) but the SPB most distal from the cell tip in the apical cell is SepH positive (red arrowhead). Bar ~ 10 µm.

Table S1 DLAP tagged kinase functionality

Name	Tag location	Phenotype	
		Null allele	DLAP Tagged strain
An-Cak1	SSSKKSTTAV*	Strong growth defect	None
CmkA	EREARERAHS*	Moderate growth defect; Increased pigment production	None
NimX^{Cdk1}	SGRARRNGFH*	Non-viable	None
SepH	LTQFEAERGS*	Strong growth defect, septation deficient	None
CkiA^{Hrr25}	GLGRQWYYEA*	Non-viable	None
CotA	YKAFNAFQAS*	Non-viable	None
An-Aurora	GSGASKDGKV*	Non-viable	None
SudD	LVSSSSRKRK*	Non-viable	None
ChkC	PVRRNAISKE*	HU sensitivity	None
SldA$^{Bub1/R1}$	FAEKKKRLEK*	Benomyl Sensitivity	None
An-IreA	RFKRYFTPLE*	Non-viable	None
BckA	YAKIRPVLEN*	Non-viable	None
An-Prp4	KH PFILRPKA*	Non-viable	None
UvsB^{ATR}	AMYIGWCAFF*	HU and DNA damage sensitivity	HU and DNA damage sensitivity
UvsB^{ATR}	*MGMSDWASVE	HU and DNA damage sensitivity	None
TorA	QHWIGWCSFW*	Non-viable	Non-viable
TorA	*MAQAGPITDV	Non-viable	Moderate growth defect
An-Cdc7	DGDDDEVDMV*	Non-viable	None
An-Cdk7	RQLDFGAIKG*	Non-viable	None
