# Supplementary material for: Application of a New Dual Localization-Affinity Purification Tag Reveals Novel Aspects of Protein Kinase Biology in Aspergillus nidulans
Source: PLoS One. 2014 Mar 5;9(3):e90911. doi: 10.1371/journal.pone.0090911 (PMC3944740; doi:10.1371/journal.pone.0090911)
Supplement: File S2 — Plasmid pCDS65 for C-terminal DLAP tagging. (PDF) [file pone.0090911.s002.pdf]

pCDS65

(For introducing a C-terminal DLAP (-GFP-S-tag) tag with a *pyrG*^{Af} marker)

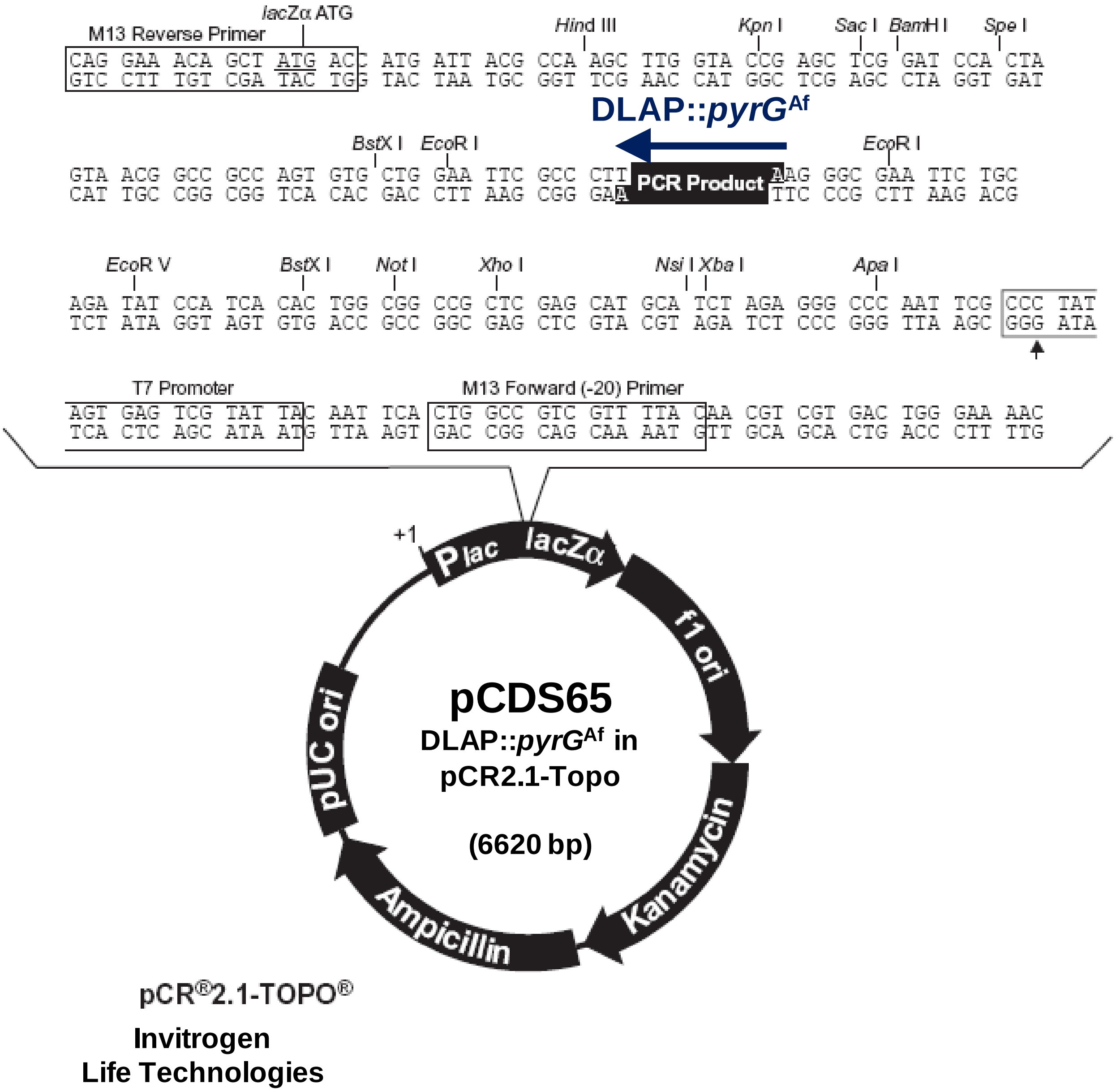

The DLAP (-GFP-S-tag::*pyrG*^{Af}) cassette is amplified using the HP116 + FN01-*pyrG* primers*

HP116	GGAGCTGGTGCAGGCGCTGGAGCC
FN01-*pyrG*	CTGTCTGAGAGGAGGCACTGATGC

-----------HP116------------------------------->
GGAGCTGGTGCAGGCGCTGGAGCCGGTGCCAGTAAAGGAGAAGAACTTTTCACTGGAGTTGTCCCAATTCTTGT
TGAATTAGATGGTGATGTTAATGGGCACAAATTTTCTGTCAGTGGAGAGGGTGAAGGTGATGCAACATACGGAAA
ACTTACCCTTAAATTTATTTGCACTACTGGAAAACTACCTGTTCCATGGCCAACACTTGTCACTACTTTCACCTATG
GTGTTCAATGCTTTTCAAGATACCCAGATCATATGAAGCGGCACGACTTCTTCAAGAGCGCCATGCCTGAGGGAT
ACGTGCAGGAGAGGACCATCTTCTTCAAAGACGACGGGAACTACAAGACACGTGCTGAAGTCAAGTTTGAGGGA
GACACCCTCGTCAACAGGATCGAGCTTAAGGGAATCGATTTCAAGGAGGACGGAAACATCCTCGGCCACAAGTT
GGAATACAACTACAACTCCCACAACGTATACATCATGGCCGACAAGCAAAAGAACGGCATCAAAGCCAACTTCAA
GACCCGCCACAACATCGAAGACGGCGGCGTGCAACTCGCTGATCATTATCAACAAAATACTCCAATTGGCGATG
GCCCTGTCCTTTTACCAGACAACCATTACCTGTCCACACAATCTGCCCTTTCGAAAGATCCCAACGAAAAGAGAG
ACCACATGGTCCTTCTTGAGTTTGTAACAGCTGCTGGGATTACACATGGCATGGATGAACTATACAAAGCTGGAG
CAGGTGCCGCTGGCGCTGGAGCTAAAGAAACCGCTGCTGCTAAATTCGAACGCCAGCACATGGACAGCGGTGC
TTAACGCCTCAAACAATGCTCTTCACCCTCTTCGCGGGTCTGAAATACCCTCACCTGGCAACAGCAATTGGCGCT
TCATGGCTGTTTTTCCGATCTCTCTACTTGTACGGCTATGTGTACTCGGGTAAGCCACAAGGCAAGGGCAGATTG
CTGGGAGGTTTCTTCTGGTTTTCTCAAGGCGCTCTGTGGGCTCTGAGTGTGTTTGGTGTTGCCAGAGACATGATC
TCTTACTGAGAGTTATTCTGTGTCTGACGAAATATGTTGTGTATATATATATATGTACGTTAAAAGTTCCGTGGAGT
TACCAGTGATTGACCAATGTTTTATCTTCTACAGTTCTGCCTGTCTACCCCATTCTAGCTGTACCTGACTACAGAA
TAGTTTAATTGTGGTTGACCCCACAGTCGGAGGCGGAGGAATACAGCACCGATGTGGCCTGTCTCCATCCAGAT
TGGCACGCAATTTTTACACGCGGAAAAGATCGAGATAGAGTACGACTTTAAATTTAGTCCCCGGCGGCTTCTATT
TTAGAATATTTGAGATTTGATTCTCAAGCAGTTGATTTGGTTGGGTCACCCTCAATTGGATAATATACCTCATTGCT
CGGCTACTTCAACTCATCAATCACCGTCATACCCCGCATATAACCCTCCATTCCCACGATGTCGTCCAAGTCGCA
ATTGACTTACGGTGCTCGAGCCAGCAAGCACCCCAATCCTCTGGCAAAGAGACTTTTTGAGATTGCCGAAGCAAA
GAAGACAAACGTTACCGTCTCTGCTGATGTGACGACAACCCGAGAACTCCTGGACCTCGCTGACCGTACGGAAG
CTGTTGGATCCAATACATATGCCGTCCAGCAATGGACTAATCAACTTTTGATGATACAGGTCTCGGTCCCTACATC
GCCGTCATCAAGACACACATCGACATCCTCACCGATTTCAGCGTCGACACTATCAATGGCCTGAATGTGCTGGCT
CAAAAGCACAACTTTTTGATCTTCGAGGACCGCAAATTCATCGACATCGGCAATACCGTCCAGAAGCAGTACCAC
GGCGGTGCTCTGAGGATCTCCGAATGGGCCCACATTATCAACTGCAGCGTTCTCCCTGGCGAGGGCATCGTCG
AGGCTCTGGCCCAGACCGCATCTGCGCAAGACTTCCCCTATGGTCCTGAGAGAGGACTGTTGGTCCTGGCAGA
GATGACCTCCAAAGGATCGCTGGCTACGGGCGAGTATACCAAGGCATCGGTTGACTACGCTCGCAAATACAAGA
ACTTCGTTATGGGTTTCGTGTCGACGCGGGCCCTGACGGAAGTGCAGTCGGATGTGTCTTCAGCCTCGGAGGAT
GAAGATTTCGTGGTCTTCACGACGGGTGTGAACCTCTCTTCCAAAGGAGATAAGCTTGGACAGCAATACCAGACT
CCTGCATCGGCTATTGGACGCGGTGCCGACTTTATCATCGCCGGTCGAGGCATCTACGCTGCTCCCGACCCGGT
TGAAGCTGCACAGCGGTACCAGAAAGAAGGCTGGGAAGCTTATATGGCCAGAGTATGCGGCAAGTCATGATTTC
CTCTTGGAGCAAAAGTGTAGTGCCAGTACGAGTGTTGTGGAGGAAGGCTGCATACATTGTGCCTGTCATTAAACG
ATGAGCTCGTCCGTATTGGCCCCTGTAATGCCATGTTTTCCGCCCCCAATCGTCAAGGTTTTCCCTTTGTTAGATT
CCTACCAGTCATCTAGCAAGTGAGGTAAGCTTTGCCAGAAACGCCAAGGCTTTATCTATGTAGTCGATAAGCAAA
GTGGACTGATAGCTTAATATGGAAGGTCCCTCAGGACAAGTCGACCTGTGCAGAAGAGATAACAGCTTGGCATC
AC***GCATCAGTGCCTCCTCTCAGACAG***AAGGGCGAATTCCAGCACACTGGCGGCCGTTACTAGTGGATCCGAG
<------FN01-*pyrG*-(Rev complement)---

***Note:** For amplification using HP116/FN01-*pyrG* primers, use an annealing and extension temperature of **72°C** to obtain a clean 2764 bp band. At lower annealing temperatures a second band of ~2000 bp may be present because HP116 binds weakly to a second site in the plasmid.

Encoded DLAP tag sequence:

GFP

**GAGAGAGAGASKGEELFTGVVPILVELDGDVNGHKFSVSGEGEGDATYGKLTLKFICTTGKLP
VPWPTLVTTFTYGVQCFSRYPDHMKRHDFFKSAMPEGYVQERTIFFKDDGNYKTRAEVKFEGD
TLVNRIELKGIDFKEDGNILGHKLEYNYNSHNVYIMADKQKNGIKANFKTRHNIEDGGVQLADHY
QQNTPIGDGPVLLPDNHYLSTQSALSKDPNEKRDHMVLLEFVTAAGITHGMDELYKAGAGAAG
AGAKETAAAKFERQHMDSGA***

S-tag
