# Supplementary material for: Application of a New Dual Localization-Affinity Purification Tag Reveals Novel Aspects of Protein Kinase Biology in Aspergillus nidulans
Source: PLoS One. 2014 Mar 5;9(3):e90911. doi: 10.1371/journal.pone.0090911 (PMC3944740; doi:10.1371/journal.pone.0090911)
Supplement: File S3 — Plasmid pCDS67 for N-terminal DLAP tagging. (PDF) [file pone.0090911.s003.pdf]

pCDS67

(For introducing an N-terminal DLAP (S-tag-GFP) tag)

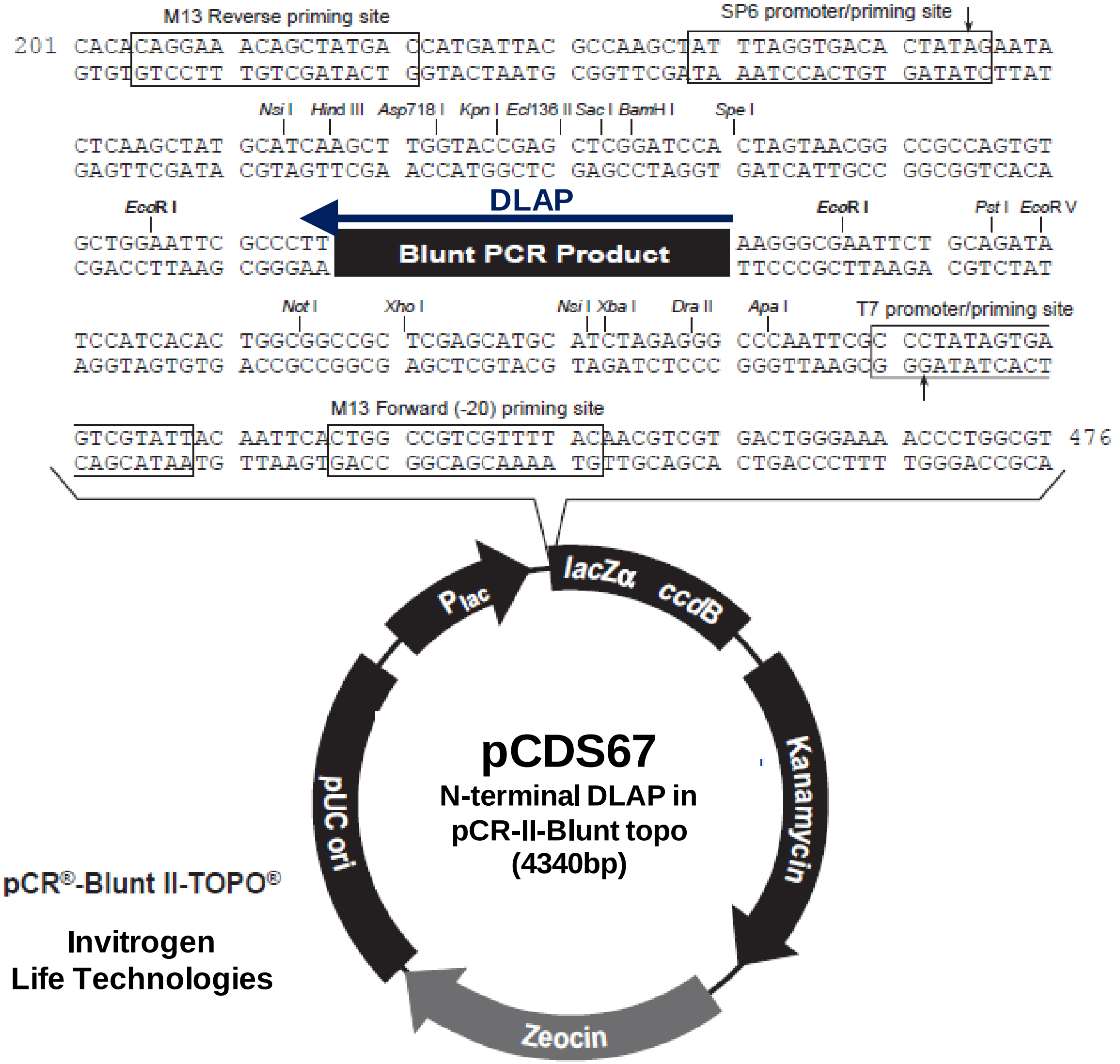

pCR®-Blunt II-TOPO®

Invitrogen
Life Technologies

The DLAP S-tag-GFP cassette is amplified using the CDS335 + CDS346 primers

CDS335 ATGCATAAAGAAACCGCTGCTG
CDS346 TCCTGCACCTGCTCCTACAC

-------CDS335------------------→
ATGCATAAAGAAACCGCTGCTGCTAAATTCGAACGCCAGCATATGGACAGCGGAGCTGGTGCAGGCGCTGGAGCC
GGTGCCAGTAAAGGAGAAGAACTTTTCACTGGAGTTGTCCCAATTCTTGTTGAATTAGATGGTGATGTTAATGGGCAC
AAATTTTCTGTCAGTGGAGAGGGTGAAGGTGATGCAACATACGGAAAACTTACCCTTAAATTTATTTGCACTACTGGA
AAACTACCTGTTCCATGGCCAACACTTGTCACTACTTTCACCTATGGTGTTCAATGCTTTTCAAGATACCCAGATCATA
TGAAGCGGCACGACTTCTTCAAGAGCGCCATGCCTGAGGGATACGTGCAGGAGAGGACCATCTTCTTCAAAGACGA
CGGGAACTACAAGACACGTGCTGAAGTCAAGTTTGAGGGAGACACCCTCGTCAACAGGATCGAGCTTAAGGGAATC
GATTTCAAGGAGGACGGAAACATCCTCGGCCACAAGTTGGAATACAACTACAACTCCCACAACGTATACATCATGGC
CGACAAGCAAAAGAACGGCATCAAAGCCAACTTCAAGACCCGCCACAACATCGAAGACGGCGGCGTGCAACTCGCT
GATCATTATCAACAAAATACTCCAATTGGCGATGGCCCTGTCCTTTTACCAGACAACCATTACCTGTCCACACAATCTG
CCCTTTCGAAAGATCCCAACGAAAAGAGAGACCACATGGTCCTTCTTGAGTTTGTAACAGCTGCTGGGATTACACATG
GCATGGATGAACTATACAAAGCTGGCGCAG***GTGTAGGAGCAGGTGCAGGA***
←CDS346-(Rev Complement)---

<u>Encoded DLAP tag sequence:</u>

S-tag GFP

**MHKETAAAKFERQHMDSGAGAGAGAGASKGEELFTGVVPILVELDGDVNGHKFSVSGEGEGDAT
YGKLTLKFICTTGKLPVPWPTLVTTFTYGVQCFSRYPDHMKRHDFFKSAMPEGYVQERTIFFKDDG
NYKTRAEVKFEGDTLVNRIELKGIDFKEDGNILGHKLEYNYNSHNVYIMADKQKNGIKANFKTRHNI
EDGGVQLADHYQQNTPIGDGPVLLPDNHYLSTQSALSKDPNEKRDHMVLLEFVTAAGITHGMDEL
YKAGAGVGAGAG**

For N-terminal tagging, land the DLAP cassette amplified from pCDS67 in frame and immediately in front of the start codon of the gene to be tagged. Selection can be achieved by targeting a nutritional marker upstream of the promoter of the gene to be tagged taking care not to interfere with the upstream gene. This can be carried out using a 5 way fusion PCR strategy [1] as shown below for N-terminal DLAP tagging of UvsB^{ATR}.

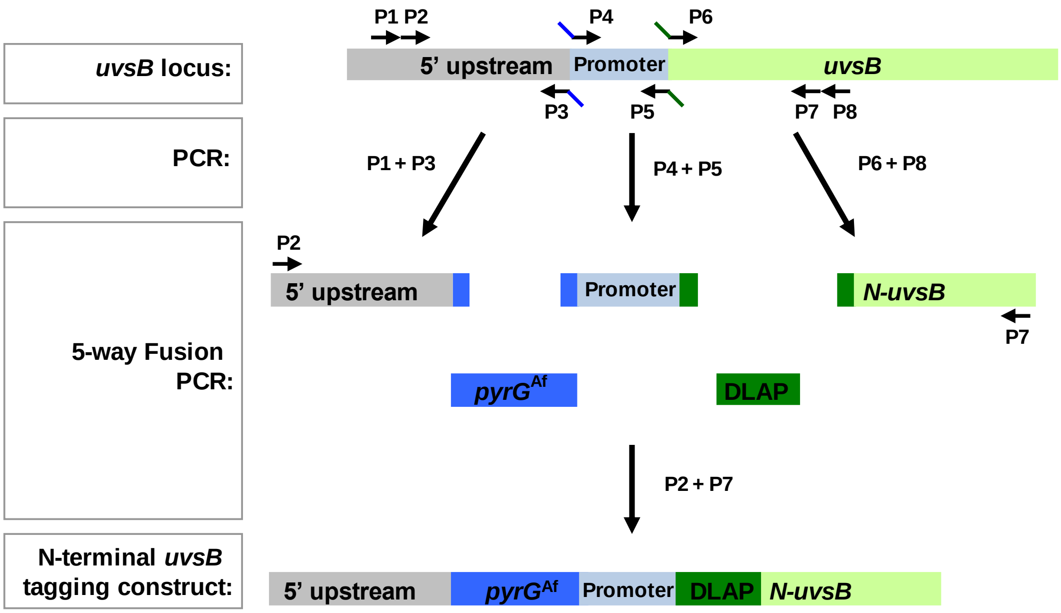

References

1. Wong KH, Todd RB, Oakley BR, Oakley CE, Hynes MJ, et al. (2008) Sumoylation in *Aspergillus nidulans*: sumO inactivation, overexpression and live-cell imaging. Fungal Genetics and Biol 45: 728-737.
