# Supplementary material for: Application of a New Dual Localization-Affinity Purification Tag Reveals Novel Aspects of Protein Kinase Biology in Aspergillus nidulans
Source: PLoS One. 2014 Mar 5;9(3):e90911. doi: 10.1371/journal.pone.0090911 (PMC3944740; doi:10.1371/journal.pone.0090911)
Supplement: File S4 — Primer List. (PDF) [file pone.0090911.s004.pdf]

Primer	Sequence	Gene/plasmid	Utility
**Universal**			
HP116	GGAGCTGGTGCAGGCGCTGGAGCC	pCDS65	GFP-S-tag::pyrG^{Af} Forward
FN01-pyrG	CTGTCTGAGAGGAGGCACTGATGC	pCDS65	GFP-S-tag::pyrG^{Af} Reverse
CDS335	ATGCATAAAGAAACCGCTGCTG	pCDS67	S-tag-GFP Forward
CDS337	CAAAGACATGATCTCTTACTGAGAG	pCDS67	pyrG^{Af} Forward
CDS346	TCCTGCACCTGCTCCTACAC	pCDS67	S-tag-GFP Reverse
**An3946 SldA$^{Bub1/R1}$**			
CDS203	*CAGCGCCTGCACCAGCTCC* CTTCTCCAATCTCTTCTTTTTCTCTGC	An3946 SldA$^{Bub1/R1}$	P2
CDS204	*CATCAGTGCCTCCTCTCAGACAG* CTCCAATTTCAATTATCCTTCCG	An3946 SldA$^{Bub1/R1}$	P3
CDS205	ACTGCCTCATCCGCTTCGAC	An3946 SldA$^{Bub1/R1}$	P1
CDS206	CAACCTGCCTTTCGGACCTC	An3946 SldA$^{Bub1/R1}$	P4
**An4563 CkiA^{Hrr25}**			
CDS208	GTACATCCACGCGAAGTCCTTCATCCAC	An4563 CkiA^{Hrr25}	P1
CDS249	*CAGCGCCTGCACCAGCTCC* GGCTTCATAATACCACTGACGACCG	An4563 CkiA^{Hrr25}	P2
CDS250	*CATCAGTGCCTCCTCTCAGACAG* TAGCTTCCTGTTTTGTGGCATGGTG	An4563 CkiA^{Hrr25}	P3
CDS251	AATGTCGGTGCAATGCGGAGG	An4563 CkiA^{Hrr25}	P4
**An6975 uvsB^{ATR}**			
CDS253	AGTGGTTCGTGGAGATGTTC	An6975 uvsB^{ATR}	5'F Diagnostic
CDS254	CGCTATACTCGGTCATGTGC	An6975 uvsB^{ATR}	P1
CDS256	*CAGCGCCTGCACCAGCTCC* AAAGAAAGCACACCAACCAATGTACATCG	An6975 uvsB^{ATR}	P2
CDS257	*CATCAGTGCCTCCTCTCAGACAG* TAGATTGAGGAAAGAGCACGG	An6975 uvsB^{ATR}	P3
CDS258	CTTCCTGTGAACCATGCTTG	An6975 uvsB^{ATR}	P4
CDS259	AACGGAACCGCACTTGCTGG	An6975 uvsB^{ATR}	3'R Diagnostic
CDS338	CAAGGTCATTACTCGCTCAG	An6975 uvsB^{ATR}	5'F Diagnostic (N-term)
CDS339	TTGGTATTCAGCTCTCGGTG	An6975 uvsB^{ATR}	P1 (N-term)
CDS340	*CTCTCAGTAAGAGATCATGTCTTTG* CCATCCAGGAATTAGTATTGC	An6975 uvsB^{ATR}	P2 (N-term)
CDS341	*CATCAGTGCCTCCTCTCAGACAG* CCTGGATGGTTAGCAAATGC	An6975 uvsB^{ATR}	P3 (N-term)
CDS342	*CAGCAGCGGTTTCTTTATGCAT* CGTAGGATAAGAATGAACCTTC	An6975 uvsB^{ATR}	P4 (N-term)
CDS343	GTGTAGGAGCAGGTGCAGGAATGGGCATGAGCGACTGGGC	An6975 uvsB^{ATR}	P5 (N-term)
CDS344	TCGACGTCAGGAAGTGCGAG	An6975 uvsB^{ATR}	P6 (N-term)
CDS345	AAACCACACGCATTGCCTTAG	An6975 uvsB^{ATR}	3'R Diagnostic (N-term)
**An4385 SepH**			
CDS261	ACATTGTCCAAGGCGAATGC	An4385 SepH	P1
CDS262	*CAGCGCCTGCACCAGCTCC* CGATCCTCGCTCCGCCTCAAAC	An4385 SepH	P2
CDS263	*CATCAGTGCCTCCTCTCAGACAG* TAATCGGTTGGTCCGTAATGG	An4385 SepH	P3
CDS264	CGGCTTGTATGAATTCCACG	An4385 SepH	P4
**An5529 CotA**			
CDS267	ACCTGACAACATCCTTCTCG	An5529 CotA	P1
CDS268	*CAGCGCCTGCACCAGCTCC* ACTGGCCTGGAAGGCGTTGAATG	An5529 CotA	P2
CDS269	*CATCAGTGCCTCCTCTCAGACAG* GGCCAGTTGAGCATGCATTTAAAG	An5529 CotA	P3
CDS270	TTCGTGCTCACTCTGGATAG	An5529 CotA	P4
**An2412 CmkA**			
CDS279	GTTGGATAACCCAGCCGAAG	An2412 CmkA	P1
CDS280	*CAGCGCCTGCACCAGCTCC* TGAGTGAGCCCGCTCGCGAG	An2412 CmkA	P2
CDS281	*CATCAGTGCCTCCTCTCAGACAG* CTCATGAAACCTCTGAATATTGG	An2412 CmkA	P3
CDS282	TGTATAGCGGAGGAAGGAG	An2412 CmkA	P4
**An4812 NIMX^{cdk1}**			
CDS283	ACAGAGTGCTTCACCGTGAC	An4812 NIMX^{cdk1}	P1
CDS284	*CAGCGCCTGCACCAGCTCC* GTGAAAGCCATTGCGGCGAG	An4812 NIMX^{cdk1}	P2
CDS285	*CATCAGTGCCTCCTCTCAGACAG* CACTAATTCGATGACTCATGAG	An4812 NIMX^{cdk1}	P3
CDS286	GTCGAGACTTGAGCTGATGG	An4812 NIMX^{cdk1}	P4
**An5982 TorA**			
CDS288	AGGATCTGTACCGTGTTCTG	An5982 TorA	P1
CDS289	*CAGCGCCTGCACCAGCTCC* CCAGAAACTGCACCATCCAATC	An5982 TorA	P2
CDS290	*CATCAGTGCCTCCTCTCAGACAG* TCTTTTCATTCCCTCCGTGG	An5982 TorA	P3
CDS291	GATACAACTGGACGTGGTCG	An5982 TorA	P4
CDS347	TGTTCGCCGTCCATTCTGTG	An5982 TorA	5'F Diagnostic (N-term)
CDS348	CTCGTACTCTCTCCCGCTTG	An5982 TorA	P1 (N-term)
CDS349	*CTCTCAGTAAGAGATCATGTCTTTG* CCGTCTTGCTTTCGAGTCAC	An5982 TorA	P2 (N-term)
CDS350	*CATCAGTGCCTCCTCTCAGAC* AGAGACGGAAAGGATAACACGC	An5982 TorA	P3 (N-term)
CDS351	*CAGCAGCGGTTTCTTTATGCAT* CTTTGAAACCGCTCCCTAGG	An5982 TorA	P4 (N-term)
CDS352	*GTGTAGGAGCAGGTGCAGGA* ATGGCGCAAGCAGGTCCGATC	An5982 TorA	P5 (N-term)
CDS353	GCTGGGCTTGAAGATACACC	An5982 TorA	P6 (N-term)
CDS354	ACCGCATTTGCAATCTTTCC	An5982 TorA	3'R Diagnostic (N-term)
**An6363 SudD^{Rio1}**			
CDS292	TTGTCACTTGGTCCATGCTG	An6363 SudD^{Rio1}	P1
CDS293	*CAGCGCCTGCACCAGCTCC* CTTGCGCTTCCTAGAGGACG	An6363 SudD^{Rio1}	P2
CDS294	*CATCAGTGCCTCCTCTCAGACAG* TGAGCAACTGGACCTTATCAC	An6363 SudD^{Rio1}	P3
CDS295	GTACACTGCCAATGACAACC	An6363 SudD^{Rio1}	P4
**An7563 ChkC**			
CDS296	TTCCGCAAGCTGGTTCTCTC	An7563 ChkC	P1

CDS297	*CAGCGCCTGCACCAGCTCC* TTCTTTCGAAATGGCGTTGCG	An7563 ChkC	P2
CDS298	*CATCAGTGCCTCCTCTCAGACAG* GTTCGACAAAATCTCAATGATGAG	An7563 ChkC	P3
CDS299	ACAGCAATGGCTAGGTCAGG	An7563 ChkC	P4
**An3450 An-Cdc7**			
CDS305	TACTGCACCGCGATATCAAG	An3450 An-Cdc7	P1
CDS306	*CAGCGCCTGCACCAGCTCC* TACCATATCCACTTCATCGTCG	An3450 An-Cdc7	P2
CDS307	*CATCAGTGCCTCCTCTCAGACAG* GATGTCTTACACGAACGGCC	An3450 An-Cdc7	P3
CDS308	TTGAACTTCGATCGGCGGTG	An3450 An-Cdc7	P4
**An4887 BckA**			
CDS309	TAGCTGCGCTGGACCAAGAG	An4887 BckA	P1
CDS310	*CAGCGCCTGCACCAGCTCC* ATTCTCAAGCACAGGACGGA	An4887 BckA	P2
CDS311	*CATCAGTGCCTCCTCTCAGACAG* AACGCTCATTTTCAACTCATG	An4887 BckA	P3
CDS312	TCGTTGGTCACGGACTCCTC	An4887 BckA	P4
**An8215 An-Cdk7 (kin28)**			
CDS313	ACTTGCTTATTGCCTCGGAC	An8215 An-Cdk7	P1
CDS314	*CAGCGCCTGCACCAGCTCC* CCCTTTTATGGCACCGAAATC	An8215 An-Cdk7	P2
CDS315	*CATCAGTGCCTCCTCTCAGACAG* CACTTTGGAACTCCGAAACAGC	An8215 An-Cdk7	P3
CDS316	TCGAAGCTCGAAGTCTCGTG	An8215 An-Cdk7	P4
**An0235 IreA**			
CDS323	CGTCAACGGTATGTCCCAAC	An0235 IreA	P1
CDS324	*CAGCGCCTGCACCAGCTCC* CTCCAATGGCGTAAAATATCTC	An0235 IreA	P2
CDS325	*CATCAGTGCCTCCTCTCAGACAG* TAGGTTGTTGCGTACTGGTTC	An0235 IreA	P3
CDS326	AAGGTAATACGCGTTGCTGG	An0235 IreA	P4
**An4936 An-Prp4**			
CDS327	ACGGAGCTTCGAGCACAAGG	An4936 An-Prp4	P1
CDS328	*CAGCGCCTGCACCAGCTCC* CGCCTTTGGGCGTAAAATAAACG	An4936 An-Prp4	P2
CDS329	*CATCAGTGCCTCCTCTCAGACAG* GTGAGTGTGCATCTAAGTGC	An4936 An-Prp4	P3
CDS330	CTATAGGAGGATGGCGGAGG	An4936 An-Prp4	P4
**An0699 An-Cak1**			
CDS331	GTGAGGCAGCTAACGAGCAC	An0699 An-Cak1	P1
CDS332	*CAGCGCCTGCACCAGCTCC* GACAGCCGTTGTAGATTTTTTCG	An0699 An-Cak1	P2
CDS333	*CATCAGTGCCTCCTCTCAGACAG* AGGCTTCTGCCGACACATACAC	An0699 An-Cak1	P3
CDS334	ACAGCCGCATTTCCCTTTAG	An0699 An-Cak1	P4
**An5815 Aurora**			
Nat3	GCGCAACGTCTTCAACATCC	An5815 Aurora	P4
Nat5	GGCTCCAGCGCCTGCACCAGCTCCTACTTTGCCATCCTTCGACG	An5815 Aurora	P2
Nat6a	GCATCAGTGCCTCCTCTCAGACAGTGTGCATTTGCATAGCAAGG	An5815 Aurora	P3
Nat8	CAATGCCATGAACGCGAACG	An5815 Aurora	P1
**GFP-S-tag control**			
CDS151	TCCATCCACCAATTGCCCAC	An6157 pyrG	P1 GFP-S-tag at pyrG
CDS152	TGGTGAGGATAGGAATTGCC	An6157 pyrG	P1 GFP-S-tag at pyrG
CDS300	CAGCGCCTGCACCAGCTCCCATGATGGCGGTTCTCCAATG	An6157 pyrG	P1 GFP-S-tag at pyrG
CDS301	CATCAGTGCCTCCTCTCAGACAGGAGTGTGAGTGGAAATGTGTAAC	An6157 pyrG	P1 GFP-S-tag at pyrG
