# Supplementary material for: Application of a New Dual Localization-Affinity Purification Tag Reveals Novel Aspects of Protein Kinase Biology in Aspergillus nidulans
Source: PLoS One. 2014 Mar 5;9(3):e90911. doi: 10.1371/journal.pone.0090911 (PMC3944740; doi:10.1371/journal.pone.0090911)
Supplement: File S5 — Strain List. (PDF) [file pone.0090911.s005.pdf]

Strain	Genotype	Origin
CDS1027	*sldA*$^{Bub1/R1}$-DLAP::*pyrG*^{Af} (AN3946); Δ*nkuA*^{Ku70}::*argB*; *pyroA* 4; *argB* 2; *pyrG* 89; *sE* 15; *nirA* 14; *wA* 3; *fwA* 1; *chaA* 1	SO451 transformant
CDS1028	*aurora*-DLAP:*pyrG*^{Af} (AN5815); Δ*nkuA*^{Ku70}::*argB*; *pyroA* 4; *argB* 2; *pyrG* 89; *sE* 15; *nirA* 14; *wA* 3; *fwA* 1; *chaA* 1	SO451 transformant
CDS1038	*sldA*$^{Bub1/R1}$-DLAP::*pyrG*^{Af} (AN3946); *nup49*-mCherry::*pyroA*^{Af}; *pyrG* 89; *pyroA* 4; *argB* 2; *fwA* 1; *wA* 3 (*nirA* 14[1] *chaA* 1[1])	CDS1027xCDS660
CDS1044	*nup82*-DLAP::*pyrG*^{Af} (AN6143); Δ*nkuA*^{Ku70}::*argB*; *pyrG* 89; *pyroA* 4; *argB* 2; *sE* 15; *nirA* 14; *wA* 3; *fwA* 1; *chaA* 1	SO451 transformant
CDS1048	*cmkA*-DLAP::*pyrG*^{Af} (AN2412); Δ*nkuA*^{Ku70}::*argB*; *pyroA* 4; *argB* 2; *pyrG* 89; *sE* 15; *nirA* 14; *wA* 3; *fwA* 1; *chaA* 1	SO451 transformant
CDS1049	*nimX*^{Cdk1}-DLAP::*pyrG*^{Af} (AN4812); Δ*nkuA*^{Ku70}::*argB*; *pyroA* 4; *argB* 2; *pyrG* 89; *sE* 15; *nirA* 14; *wA* 3; *fwA* 1; *chaA* 1	SO451 transformant
CDS1050	*cotA*-DLAP::*pyrG*^{Af} (AN5529); Δ*nkuA*^{Ku70}::*argB*; *pyroA* 4; *argB* 2; *pyrG* 89; *sE* 15; *nirA* 14; *wA* 3; *fwA* 1; *chaA* 1	SO451 transformant
CDS1051	*sudD*-DLAP::*pyrG*^{Af} (AN6363); Δ*nkuA*^{Ku70}::*argB*; *pyroA* 4; *argB* 2; *pyrG* 89; *sE* 15; *nirA* 14; *wA* 3; *fwA* 1; *chaA* 1	SO451 transformant
CDS1052	*sepH*-DLAP::*pyrG*^{Af} (AN4385); Δ*nkuA*^{Ku70}::*argB*; *pyroA* 4; *argB* 2; *pyrG* 89; *sE* 15; *nirA* 14; *wA* 3; *fwA* 1; *chaA* 1	SO451 transformant
CDS1053	*ckiA*^{Hrr25}-DLAP::*pyrG*^{Af} (AN4563); Δ*nkuA*^{Ku70}::*argB*; *pyroA* 4; *argB* 2; *pyrG* 89; *sE* 15; *nirA* 14; *wA* 3; *fwA* 1; *chaA* 1	SO451 transformant
CDS1060	*An-cak* 1-DLAP::*pyrG*^{Af} (AN0699); Δ*nkuA*^{Ku70}::*argB*; *pyroA* 4; *argB* 2; *pyrG* 89; *sE* 15; *nirA* 14; *wA* 3; *fwA* 1; *chaA* 1	SO451 transformant
CDS1061	*chkC*-DLAP::*pyrG*^{Af} (AN7563); Δ*nkuA*^{Ku70}::*argB*; *pyroA* 4; *argB* 2; *pyrG* 89; *sE* 15; *nirA* 14; *wA* 3; *fwA* 1; *chaA* 1	SO451 transformant
CDS1062	*An-cdc7*-DLAP::*pyrG*^{Af} (AN3450); Δ*nkuA*^{Ku70}::*argB*; *pyroA* 4; *argB* 2; *pyrG* 89; *sE* 15; *nirA* 14; *wA* 3; *fwA* 1; *chaA* 1	SO451 transformant
CDS1063	*bckA*-DLAP::*pyrG*^{Af} (AN4887); Δ*nkuA*^{Ku70}::*argB*; *pyroA* 4; *argB* 2; *pyrG* 89; *sE* 15; *nirA* 14; *wA* 3; *fwA* 1; *chaA* 1	SO451 transformant
CDS1064	*An-prp* 4-DLAP::*pyrG*^{Af} (AN4936); Δ*nkuA*^{Ku70}::*argB*; *pyroA* 4; *argB* 2; *pyrG* 89; *sE* 15; *nirA* 14; *wA* 3; *fwA* 1; *chaA* 1	SO451 transformant
CDS1065	*An-cdk* 7-DLAP::*pyrG*^{Af} (AN8285); Δ*nkuA*^{Ku70}::*argB*; *pyroA* 4; *argB* 2; *pyrG* 89; *sE* 15; *nirA* 14; *wA* 3; *fwA* 1; *chaA* 1	SO451 transformant
CDS1066	*ireA*-DLAP::*pyrG*^{Af} (AN0235); Δ*nkuA*^{Ku70}::*argB*; *pyroA* 4; *argB* 2; *pyrG* 89; *sE* 15; *nirA* 14; *wA* 3; *fwA* 1; *chaA* 1	SO451 transformant
CDS1068	*pyrG*::DLAP::*pyrG*^{Af}; Δ*nkuA*^{Ku70}::*argB*; *pyroA* 4; *argB* 2; *pyrG* 89; *sE* 15; *nirA* 14; *wA* 3; *fwA* 1; *chaA* 1	SO451 transformant
CDS1073	*pyrG*^{Af}::*uvsB* promoter::DLAP-*uvsB*^{ATR} (AN6975); Δ*nkuA*^{Ku70}::*argB*; *pyroA* 4; *argB* 2; *pyrG* 89; *sE* 15; *nirA* 14; *wA* 3; *fwA* 1; *chaA* 1	SO451 transformant
CDS1074	*pyrG*^{Af}::*torA* promoter::DLAP-*torA* (AN5982); Δ*nkuA*^{Ku70}::*argB*; *pyroA* 4; *argB* 2; *pyrG* 89; *sE* 15; *nirA* 14; *wA* 3; *fwA* 1; *chaA* 1	SO451 transformant
CDS1075	*uvsB*^{ATR}-GFP-S-tag::*pyrG*^{Af} (AN6975); Δ*nkuA*$^{K u70}$::*argB*; *pyroA* 4; *argB* 2; *pyrG* 89; *sE* 15; *nirA* 14; *wA* 3; *fwA* 1; *chaA* 1	SO451 transformant
CDS1079	*An-cdc7*-DLAP::*pyrG*^{Af} (AN3450); *nup49*-mCherry::*pyroA*^{Af}; *pyrG* 89; *pyroA* 4; *argB* 2; *wA* 3; *fwA* 1 (*chaA* 1[1], *nirA* 14[1])	CDS1062xCDS660
CDS1080	*ckiA*^{Hrr25}-DLAP::*pyrG*^{Af} (AN4563); *nup49*-mCherry::*pyroA*^{Af}; *pyrG* 89; *pyroA* 4; *argB* 2; *fwA* 1; *wA* 3 (*chaA* 1[1] *nirA* 14[1])	CDS1053xCDS660
CDS1081	*ckiA*^{Hrr25}-DLAP::*pyrG*^{Af} (AN4563); *gcp* 3-mCherry::*riboB*^{Af}; *pyrG* 89; *argB* 2; *fwA* 1; *wA* 3 (*nirA* 14[1] *chaA* 1[1] *riboB* 2[1])	CDS1053 x CDS655
CDS1082	*An-cdc7*-DLAP::*pyrG*^{Af} (AN3450); *gcp* 3-mCherry::*riboB*^{Af}; *pyrG* 89; *argB* 2; *fwA* 1; *wA* 3 (*nirA* 14[1] *chaA* 1[1] *riboB* 2[1])	CDS1062xCDS655
CDS1085	*nimX*^{Cdk1}-DLAP::*pyrG*^{Af} (AN4812); *nup49*-mCherry::*pyroA*^{Af}; *pyrG* 89; *pyroA* 4; *argB* 2; *fwA* 1; *wA* 3 (*nirA* 14[1] *chaA* 1[1])	CDS1049xCDS660
CDS1097	*sepH*-DLAP::*pyrG*^{Af} (AN4385); *gcp* 3-mCherry::*riboB*^{Af}; *pyrG* 89; *argB* 2; *fwA* 1; *wA* 3 (*riboB* 2[1] *sE* 14[1] *nirA* 14[1] *chaA* 1[1])	CDS1052xCDS1092
CDS1158	*nup120*-mCherry::*pyrG*^{Af}; GFP-*tubA*; *pyrG* 89; *argB* 2; *wA* 3; *fwA* 1; *chaA* 1 (*sE* 15[1] *nirA* 14[1])	CDS1112xCDS527
CDS1161	Δ*uvsB*::*pyrG*^{Af}; *pyrG* 89; *wA* 3 (*fwA* 1[1] *yA* 2[1])	CDS1135xFN45
CDS1164	*An-cdc7*-DLAP::*pyrG*^{Af} (AN3450); *ndc80*-mCherry::*pyroA*^{Af}; *pyrG* 89; *pyroA* 4; *argB* 2; *fwA* 1; *wA* 3 *fwA* 1 (*chaA* 1[1], *nirA* 14[1])	CDS1079xCDS643
CDS1165	*ckiA*^{Hrr25}-DLAP::*pyrG*^{Af} (AN4563); *ndc80*-mCherry::*pyroA*^{Af}; *pyrG* 89; *pyroA* 4; *argB* 2; *fwA* 1; *chaA* 1; *wA* 3 (*chaA* 1[1] *sE* 15[1] *nirA* 14[1])	CDS1080xCDS643
CDS1168	Δ*sldA*::*pyrG*^{Af}; *mad1*-GFP::*pyroA*^{Af}; *ndc80*-CR::*pyroA*^{Af}; *pyrG* 89; *pyroA* 4; *argB* 2; *chaA* 1; *wA* 3 (*sE* 15[1] *nirA* 14[1] *fwA* [1])	KID104xCDS830
CDS1170	*mad1*-CR::*pyrG*^{Af}; *sldA*$^{Bub1/R1}$-DLAP::*pyrG*^{Af} (AN3946); *argB* 2; *chaA* 1; *wA* 3 (*nirA* 14[1] *sE* 15[1] *fwA* 1[1])	CDS675xCDS1027
CDS1176	*sepH*-DLAP::*pyrG*^{Af} (AN4385); *pyrG* 89; *argB* 2::*gpdp*::*stuA C-term*-DsRedT4-*argB* (NLS-DsRed); *wA* 3 (*yA* 1[1]; *fwA* 1[1] *chaA* 1[1] *sE* 15[1] *nirA* 14[1])	CDS1052 x CDS396
KID85	*ΔuvsB*::*pyrG*^{Af}; *ΔnkuA*^{Ku70}*::argB; pyroA4; argB2; pyrG89; sE15; nirA14; wA3; fwA1; chaA1*	
SO451	Δ*nkuA*^{Ku70}::*argB*; *pyroA* 4; *argB* 2; *pyrG* 89; *sE* 15; *nirA* 14; *wA* 3; *fwA* 1; *chaA* 1	
SO779	*nup82*-Stag::*pyrG*^{Af} (AN6143); Δ*nkuA*^{Ku70}::*argB*; *pyroA* 4; *argB* 2; *pyrG* 89; *sE* 15; *nirA* 14; *wA* 3; *fwA* 1; *chaA* 1	SO451 transformant
HA365 ΔAn-cdc7	*ΔAn-cdc7::pyrG*^{Af} *(AN3450); pyrG* 89*; pyroA* 4 *ΔnkuA::argB*; *argB/argB2*; *Histone H1-mCherry::pyroA*^{Af}; *GFP-tubA//pyrG89; pyroA4 ΔnkuA::argB; argB/argB2; Histone H1-mCherry::pyroA*^{Af}*; GFP-tubA*	HA365 transformant

[1] nutritional or color markers which could be covered by, or be to recessive to, other markers in the strain

^{Af} genes from *Aspergillus fumigatus* used for complementation of the corresponding *A. nidulans* nutritional mutations
